# Safety of a silicone elastomer vaginal ring as potential microbicide delivery method in African women: A Phase 1 randomized trial

**DOI:** 10.1371/journal.pone.0196904

**Published:** 2018-05-29

**Authors:** Annaléne Nel, Janine Martins, Linda-Gail Bekker, Gita Ramjee, Gileard Masenga, Helen Rees, Neliëtte van Niekerk

**Affiliations:** 1 International Partnership for Microbicides, Silver Spring, Maryland, United States of America; 2 Desmond Tutu HIV Centre, University of Cape Town, Cape Town, South Africa; 3 South African Medical Research Council, Durban, South Africa; 4 Kilimanjaro Christian Medical Centre, Moshi, Tanzania; 5 Reproductive Health and HIV Research Unit, Johannesburg, South Africa; Burnet Institute, AUSTRALIA

## Abstract

**Background:**

Women in sub-Saharan Africa are in urgent need of female-initiated human immunodeficiency virus (HIV) preventative methods. Vaginal rings are one dosage form in development for delivery of HIV microbicides. However, African women have limited experience with vaginal rings.

**Objectives:**

This Phase I, randomized, crossover trial assessed and compared the safety, acceptability and adherence of a silicone elastomer placebo vaginal ring, intended as a microbicide delivery method, inserted for a 12-week period in healthy, HIV-negative, sexually active women in South Africa and Tanzania.

**Methods:**

170 women, aged 18 to 35 years were enrolled with 88 women randomized to Group A, using a placebo vaginal ring for 12 weeks followed by a 12-week safety observation period. 82 women were randomized to Group B and observed for safety first, followed by a placebo vaginal ring for 12 weeks. Safety was assessed by clinical laboratory assessments, pelvic/colposcopy examinations and adverse events. Possible carry-over effect was addressed by ensuring no signs or symptoms of genital irritation at crossover.

**Results:**

No safety concerns were identified for any safety variables assessed during the trial. No serious adverse events were reported considered related to the placebo vaginal ring. Vaginal candidiasis was the most common adverse event occurring in 11% of participants during each trial period. Vaginal discharge (2%), vaginal odour (2%), and bacterial vaginitis (2%) were assessed as possibly or probably related to the vaginal ring. Thirty-four percent of participants had sexually transmitted infections (STIs) at screening, compared to 12% of participants who tested positive for STIs at crossover and the final trial visit. Three participants (2%) tested HIV positive during the trial.

**Conclusions:**

The silicone elastomer vaginal ring had no safety concerns, demonstrating a profile favorable for further development for topical release of antiretroviral-based microbicides.

## Introduction

Women-initiated human immunodeficiency virus (HIV) prevention methods are urgently needed in sub-Saharan Africa where women account for 56% of new HIV infections [1]. Most microbicides currently in development for the prevention of HIV infection through male-to-female intercourse are antiretroviral-based vaginally applied products such as gels and vaginal rings [2]. Vaginal rings have been marketed for the treatment of menopausal symptoms since the mid 1990s, and for birth control since 2002 in the United States of America (USA) and Europe. In Africa however, women have limited experience with the use of vaginal rings. NuvaRing® is marketed for contraception in Egypt and Morocco, and Estring® for menopausal symptoms in South Africa. A study conducted among female sex workers and their clients in Nairobi, Kenya, revealed that this population was receptive to the idea of using a vaginal ring as a potential HIV prevention method, but highlighted the importance of cultural attitudes and practices in personal healthcare choices [3].

Vaginal rings containing dapivirine, a non-nucleoside reverse transcriptase inhibitor (NNRTI), in matrix and reservoir designs, and with a base composition similar to that of Femring®, have been tested for safety and pharmacokinetics in European women since 2004 by International Partnership for Microbicides (IPM) [4–8]. Results from these trials have shown that the dapivirine-containing rings, when used continuously for 28 days, were found to be safe and well tolerated, and were considered acceptable by both the women and their male partners [5,6]. Efficacy results demonstrates up to 31% reduction in HIV risk in these trials [9,10].

The trial described herein, IPM 011, was the first vaginal ring trial conducted in Africa, prior to the publication of the Phase III efficacy results [9,10], and was designed to assess the safety and acceptability of a silicone elastomer placebo ring, intended as a microbicide delivery method in women for the prevention of HIV infection through male-to-female intercourse. Qualitative and quantitative measures that report on vaginal ring acceptability, participant adherence, ring expulsions, ring removals, and participant product use during the trial, are presented in two separately published papers [11,12]. Here we report on the safety outcomes of the placebo vaginal ring and participant tolerability of the ring when used continuously over a 3-month period.

## Materials and methods

### Trial design

**Fig 1:**
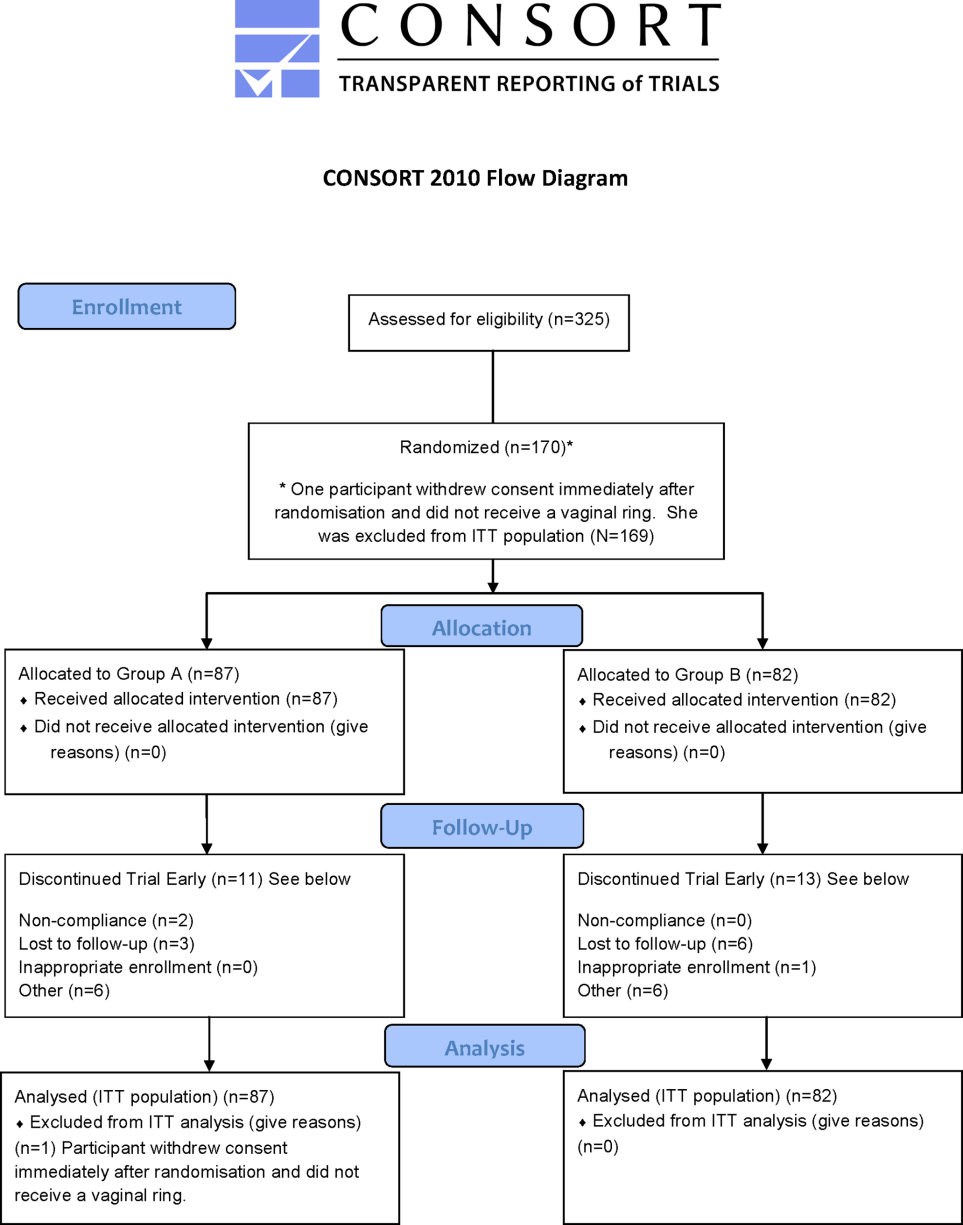
Participant flowchart.

This was a Phase I, open-label, crossover, randomized trial conducted among 170 healthy, HIV-negative, sexually active women (Fig 1).

**Fig 2:**
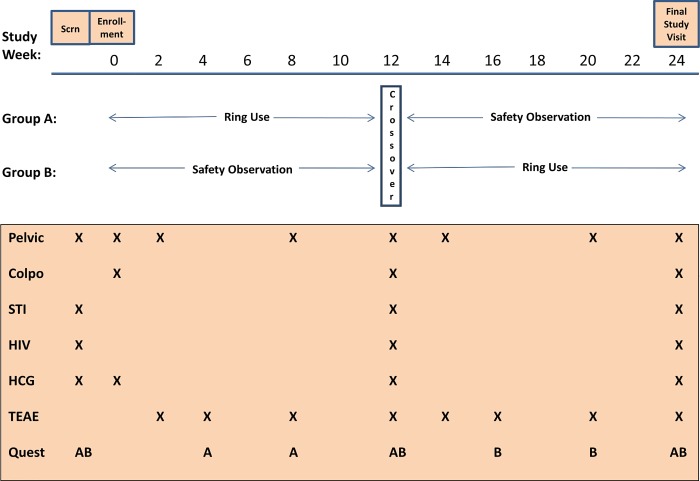
Trial design and overview of safety assessments. Group A: Participants in this group participated in a placebo vaginal ring regimen for the first 12 weeks of the trial where after they crossed over to a 12-week observational period without a ring. Group B: Participants in this group participated in an observational period for the first 12 weeks of the trial where after they crossed over to a 12-week placebo vaginal ring period. A = Group A; B = Group B, Colpo = colposcopic examination; HCG = human chorionic gonadotropin; HIV = human immunodeficiency virus; Pelvic = pelvic examination; Quest = questionnaire; Scrn = screening; STI = sexually transmitted infection; TEAE = treatment emergent adverse event.

Recruitment for the trial started on 19 April 2007 and the last participant follow-up visit was conducted on 15 March 2010. Participants were enrolled at three research centres in South Africa (Johannesburg, Durban and Cape Town) and one research centre in Moshi, Tanzania. Since this was an open-label trial and participants used a placebo ring, a crossover trial design was employed to evaluate safety (each woman served as her own control). In the crossover design, participants were randomly assigned in a 1:1 ratio to one of two groups, Group A (n = 88) or Group B (n = 82). Both groups participated in two regimens, namely a ring regimen and an observational safety regimen (with no ring use); each regimen had a duration of 12 weeks. Women in Group A were assigned to participate in the ring use regimen first, followed by a 12-week observational safety period; whereas women in Group B were assigned to participate in the observational safety regimen first followed by the 12-week ring regimen (Fig 2).

Participants were deemed eligible for enrollment if they were healthy, HIV negative, self-reported sexually active (defined as one penetrative vaginal coital act per month for the last 3 months prior to enrolment), not pregnant or breastfeeding, willing to use a stable form of contraception (oral contraceptives, long-acting injectable progestins, intrauterine devices or were surgically sterilized), had a regular menstrual cycle, had a normal appearing cervix and vagina, had normal Pap test results, had no recent gynecological surgery, and were asymptomatic for genital and sexually transmitted infections (STIs) (diagnosed STIs were treated for at least 72 hours prior to enrollment).

HIV/STI risk-reduction counseling, including the dispensing of male condoms, as well as contraceptive counseling was provided to participants at each scheduled visit. Women were instructed to refrain from using concomitant vaginal products or other objects, including female condoms for the duration of the trial. Pre- and post-HIV test counseling was provided during the screening, crossover and final trial visits when scheduled HIV-1 rapid testing was performed.

During the ring use regimen, eligible participants were dispensed with one vaginal ring which they self-inserted under supervision of the Investigator who then verified its proper placement. Participants were provided with vaginal ring adherence counseling during the ring use period and were counseled to refrain from removing the ring. Research staff instructed participants on how to clean and re-insert the vaginal ring in the case of accidental expulsion. If the ring was expelled in such a manner that the participant was unwilling to re-insert it, *e*.*g*. during urination or a bowel movement, the participant had to return the ring to the clinic, and depending on the availability of investigational product and evaluation by the Investigator, another ring was in most cases dispensed to the participant. Participants were requested to continue ring use during menses. Participant vaginal ring acceptability was assessed via interviewer-administered questionnaires at the enrolment visit and after 4, 8, and 12 weeks of ring use. Results of these assessments are presented in separate publications [11,12].

As part of the trial’s primary objective to assess the safety of the placebo vaginal ring, the trial was designed to compare gynaecological adverse events (AEs) that occurred with the placebo ring in place to AEs that occurred during the observational regimen (no ring use period). This design improved the trial’s ability to evaluate safety in each participant, since vaginal problems (*e*.*g*. bacterial vaginosis, yeast infections, urinary tract infections, and vaginal symptoms) occur very commonly in “healthy” populations, even in the absence of a vaginal delivery device. To avoid a potential carry-over effect, the participant had to be asymptomatic for genital infections and negative for findings upon pelvic/speculum and colposcopic examination at the time of crossover between trial regimens. Participants who presented with symptoms of genital infections at the crossover visit were prescribed treatment for the infections and were assessed by the Investigator prior to commencing with the next trial regimen.

### Safety assessments

An overview of the clinical safety assessments performed during the trial is presented in Fig 2. Safety assessments included pelvic/speculum and colposcopy examinations, a general physical examination, testing for pregnancy, HIV-1 and STIs that included trichomonas, gonorrhoea, and chlamydia. Women were also assessed for the presence of bacterial vaginosis using Amsel criteria. The monitoring of AEs was performed throughout the trial.

Participants with a positive HIV or pregnancy test result were not enrolled but were referred to local health facilities for social support or other medical (including prenatal) services as clinically indicated.

AEs and pelvic/speculum and colposcopy examination findings were graded by the Investigator according to the National Institutes of Health (NIH) Division of AIDS (DAIDS) Table for Grading the Severity of Adult and Pediatric Adverse Events [13]. Causality of AEs was assessed by the Investigator as related to the investigational product (defined as “definitely related”, “probably related” or “possibly related”) or not related (defined as “probably not related” or “not related”).

### Ethics

Written informed consent was obtained from each participant prior to any trial-related procedures. Ethics and regulatory approvals were obtained from the appropriate ethics committees of each research center and the national regulatory authorities of the respective countries. The individual research centers initiated participant recruitment after obtaining approval from the relevant ethics committees. The following ethics committees provided approval for the research centers in South Africa: the University of the Witwatersrand Human Subjects Research Ethics Committee on 21 March 2007, the University of KwaZulu-Natal Biomedical Research Ethics Committee on 30 July 2007, and the University of Cape Town Health Sciences Faculty Research Ethics Committee on 22 April 2008. The trial was approved in Tanzania by the Harvard School of Public Health Human Subjects Committee on 21 March 2007, the Kilimanjaro Christian Medical Centre Research and Ethics Committee on 13 March 2007, and the National Institute for Medical Research on 15 March 2007. The trial was registered in the ClinicalTrials.gov database (NCT00469170) and was conducted in full compliance with the ICH-GCP guidelines. Registration of the trial was inadvertently done 14 days after the start of participant recruitment. As there was no active product involved in the trial, initial registration was not required by the medical council but advised once ethics approval was received. The authors confirm that all ongoing and related trials for this vaginal ring are registered.

### Investigational product

The vaginal ring used in this trial contained no active pharmaceutical ingredient. The placebo ring was manufactured by Warner Chilcott, Inc. (Rockaway, New Jersey, USA), and contained the same excipient material used in Femring®. The ring was composed of silicone elastomer with a normal propylorthosilicate cross-linker, and cured by a condensation reaction using stannous octoate as the catalyst [14]. The ring had an outer diameter of 56 mm, and cross-sectional diameter of 7.6 mm.

### Statistics

As this was an exploratory trial, no formal sample size calculation was performed. The primary safety endpoints consisted of three categories of safety outcomes: pelvic/speculum and colposcopy examination findings; incident HIV infections, STIs and pregnancy incidence; and reported AEs. McNemar’s test was used to evaluate the statistical significance of differences in AE rates and in prevalence of STI infections between regimens. Statistical analyses were conducted using the statistical software SAS^®^ (version 9.2, SAS Institute Inc, Cary, North Carolina, USA). Occurrence rates of the selected safety endpoints were calculated and summarized by trial group, regimen and research centre. AEs were coded using version 10.0 of the Medical Dictionary for Regulatory Activities (MedDRA).

## Results

### Participant disposition

One-hundred-and-seventy (170) healthy, HIV negative women were enrolled in the trial with 50 participants enrolled at each of the research centers in Johannesburg, Durban and Tanzania, and 20 participants at the research center in Cape Town.

Eighty-eight (88) participants were included in Group A, and 82 participants in Group B. A total of 145 (85.3%) participants completed the trial (Group A: 76/88; 86.4%; Group B: 69/82; 84.1%). One participant in Group A withdrew consent immediately after enrollment and as a result did not receive a vaginal ring. No post-randomization data were collected for this participant and she was excluded from the analysis population (N = 169). The most common reason for early trial discontinuation in both groups (n = 24) was loss to follow-up (n = 9), followed by non-compliance (n = 2), inappropriate enrollment (n = 1) and other reasons (n = 12) such as visits outside the protocol-specified 5-day window period, consent withdrawn, missed visits, vaginal infections, and not meeting the trial’s eligibility criteria at the crossover visit.

### Demographic data

The median age of the enrolled women across the two trial groups (Groups A and B) was 27 years (range: 18–35) (Table 1). With the exception of nine Indian women and one woman of mixed ethnicity, all trial participants were of Black ethnicity. According to their relationship status and sexual history, the majority of participants were not married (113/170; 66.5%). All participants had a main sex partner and with the exception of four participants (two in each trial group) who reported having two or more sex partners, all women had only one sex partner prior to enrollment.

**Table 1:** Demographic and other baseline characteristics.

Characteristics of Trial	Group A	Group B
Participants	(N = 88)	(N = 82)
	n (%)	n (%)
**Race**		
Black	83 (94.3%)	77 (93.9%)
Coloured^a^	0	1 (1.2%)
Indian	5 (5.7%)	4 (4.9%)
**Age (years)**		
Median (range)	27.5 (18–35)	27.1 (18–35)
**BMI (kg/m**^**2**^**)**		
Mean (SD) / range	27.5 (6.1) / 17.6–43.2	27.7 (5.3) / 19.8–41.7
**Marital Status (%)**		
Not married	64 (72.7%)	49 (59.8%)
Married	21 (23.9%)	30 (36.6%)
Separated	1 (1.1%)	0
Divorced	1 (1.1%)	2 (2.4%)
Widowed	1 (1.1%)	1 (1.2%)
**Has a main sex partner**		
Yes	88 (100.0%)	82 (100.0%)
**Number of sex partners in past 3 months**		
1	86 (97.7%)	80 (97.6%)
2+	2 (2.3%)	2 (2.4%)
**Perceived risk of getting HIV**	**N = 87**	**N = 81**
**compared to others**		
High	9 (10.4%)	11 (13.6%)
About the same	13 (14.9%)	11 (13.6%)
Low	65 (74.7%)	59 (72.8%)
**Has ever used a male condom**		
Yes	73 (82.9%)	66 (80.5%)
No	15 (17.1%)	16 (19.5%)
**Number of sex acts with main partner in past 7 days**		
Median (range)	2 (0–8)	1 (0–14)
**Condom use in past 7 days**	**N = 70**	**N = 71**
**with main partner (among**		
**those reporting sex in past 7**		
**days) (N = 141)**		
None	22 (31.4%)	19 (26.8%)
Some	4 (5.7%)	4 (5.6%)
Every time had sex	44 (62.9%)	48 (67.6%)

BMI = Body mass index; HIV = Human immunodeficiency virus; SD = Standard deviation

^a^ “Coloured” is a national ethnic classification used in South Africa that describes a person with mixed racial/ethnic origins.

Overall, more than 80% of the participants reported having experience with the use of a male condom (139/170; 81.8%). The majority of the remaining 18% who reported never having used a male condom before was enrolled at the research center in Tanzania. This was also observed in the report of condom use in the 7 days prior to enrolment. Overall, the median number of sex acts with their main sex partner in the week before enrollment was two for Group A (range: 0–8) and one for Group B (range: 0–14). Just over 70% of the participants in the trial perceived their risk of acquiring HIV to be lower than that of others in their community (124/168; 73.8%), compared to 11.9% (20/168) who perceived themselves to be at high risk, and 14.3% (24/168) who perceived themselves to be at about the same risk (Table 1).

### Safety data

#### Adverse events

Just over 80% of participants in the trial (138/169; 81.7%) experienced at least one AE. In the ring intervention period, 65.5% of participants in Group A and 60.6% of participants in Group B were reported with AEs, compared to 59.5% and 73.2% for Groups A and B, respectively, in the observation period (Table 2).

**Table 2:** Incidence of adverse events that occurred in at least 5% of participants, regardless of causality.

MedDRA Preferred Term	Reported Severity (DAIDS Grade)	Ring Intervention Phase			Observation Phase				
		Group A	Group B	Total	Group A	Group B	Total	Overall Total	p-value
		N = 87	N = 71	N = 158	N = 79	N = 82	N = 161	N = 169	
		n (%)	n (%)	n (%)	n (%)	n (%)	n (%)	n (%)	
**Participant with any adverse event**		**57 (65.5%)**	**43 (60.6%)**	**100 (63.3%)**	**47 (59.5%)**	**60 (73.2%)**	**107 (66.5%)**	**138 (81.7%)**	0.6068
Vaginal candidiasis	Grade 1, 2	12 (13.8%)	6 (8.5%)	18 (11.4%)	8 (10.1%)	9 (11.0%)	17 (10.6%)	32 (18.9%)	0.8527
Influenza-like illness	Grade 1, 2	7 (8.0%)	5 (7.0%)	12 (7.6%)	4 (5.1%)	12 (14.6%)	16 (9.9%)	26 (15.4%)	0.4142
Metrorrhagia	Grade 1, 2	5 (5.7%)	4 (5.6%)	9 (5.7%)	4 (5.1%)	13 (15.9%)	17 (10.6%)	23 (13.6%)	0.0736
Vaginitis bacterial	Grade 1, 2	8 (9.2%)	7 (9.9%)	15 (9.5%)	6 (7.6%)	7 (8.5%)	13 (8.1%)	23 (13.6%)	0.6374
Headache	Grade 1, 2	12 (13.8%)	1 (1.4%)	13 (8.2%)	5 (6.3%)	5 (6.1%)	10 (6.2%)	21 (12.4%)	0.4913
Gynaecological chlamydia Infection	Grade 1, 2	2 (2.3%)	4 (5.6%)	6 (3.8%)	8 (10.1%)	1 (1.2%)	9 (5.6%)	15 (8.9%)	0.4386
Upper respiratory tract infection	Grade 1, 2	4 (4.6%)	1 (1.4%)	5 (3.2%)	1 (1.3%)	9 (11.0%)	10 (6.2%)	15 (8.9%)	0.1967
Vaginal discharge	Grade 1	2 (2.3%)	5 (7.0%)	7 (4.4%)	2 (2.5%)	1 (1.2%)	3 (1.9%)	10 (5.9%)	0.2059

DAIDS = Division of AIDS; MedDRA = Medical Dictionary for Regulatory Activities

The majority of AEs reported were of mild (Grade 1) intensity. Vaginal candidiasis was reported most often and occurred in 18 (18/158; 11.4%) participants in the ring intervention period and 17 (17/161; 10.6%) participants in the observation period. For most participants the event was assessed by the Investigator as mild in severity and not related (22/32; 68.8%) or probably not related (8/32; 25.0%) to vaginal ring use; whereas for two women the event was assessed as possibly related to product use (Table 3).

**Table 3:** Incidence of adverse events assessed by the investigator as possibly or probably related to the vaginal ring during the intervention phase.

MedDRA System Preferred Term	Ring Intervention Phase		
	Group A	Group B	Total
	N = 87	N = 71	N = 158
	n (%)	n (%)	n (%)
**Any product related event**^**a**^	**11 (12.6%)**	**8 (11.3%)**	**19 (12.0%)**
Vaginal discharge	0 (0%)	3 (4.2%)	3 (1.9%)
Vaginal odour	3 (3.4%)^b^	0 (0%)	3 (1.9%)
Vaginitis bacterial	2 (2.3%)	1 (1.4%)	3 (1.9%)
Abdominal pain lower	2 (2.3%)	0 (0%)	2 (1.3%)
Cervix erythema	2 (2.3%)	0 (0%)	2 (1.3%)
Vaginal candidiasis	1 (1.1%)	1 (1.4%)	2 (1.3%)
Vulvovaginal discomfort	0 (0%)	2 (2.8%)^b^	2 (1.3%)
Abdominal tenderness	1 (1.1%)	0 (0%)	1 (0.6%)
Cervical discharge	0 (0%)	1 (1.4%)	1 (0.6%)
Cervix haemorrhage uterine	1 (1.1%)	0 (0%)	1 (0.6%)
Coital bleeding	1 (1.1%) ^b^	0 (0%)	1 (0.6%)
Genital discomfort	0 (0%)	1 (1.4%) ^b^	1 (0.6%)
Genital erythema	0 (0%)	1 (1.4%)	1 (0.6%)
Gynaecological chlamydia infection	0 (0%)	0 (0%)	1 (0.6%)
Uterine pain	0 (0%)	1 (1.4%)	1 (0.6%)

MedDRA = Medical Dictionary for Regulatory Activities

^**a**^ No adverse events were considered by the Investigator as definitely related to placebo vaginal ring use during the trial. All product-related events were assessed as possibly related to the vaginal ring, unless otherwise indicated.

^b^ Included an event assessed as probably related to the placebo vaginal ring.

Other events of the reproductive system that occurred in more than 5% of participants were metrorrhagia, bacterial vaginitis, chlamydia infection, and vaginal discharge (Table 2). Most of these events were assessed as mild in severity.

The most commonly reported non-gynecological events, experienced in ≥ 10% of participants, included influenza-like illness, headache, and upper respiratory tract infection (Table 2). The majority of occurrences of these events were assessed as mild in intensity.

No AEs were regarded by the Investigator as definitely related to vaginal ring use. Four events (vaginal odor, vulvovaginal discomfort, coital bleeding, and genital discomfort) that occurred during the ring intervention period were assessed as probably related to ring use, each in a single participant (Table 3). All possibly related events occurred in < 2% of participants. The incidence of product-related events reported in the ring intervention period was comparable between Groups A and B (12.6% and 11.3%, respectively) (Table 3).

One serious AE was reported during the trial: a participant in Group A experienced appendicitis during the ring intervention period (after 44 days of ring use) and underwent an appendectomy. The participant continued with uninterrupted ring use and completed the trial successfully. The event was assessed by the Investigator as not related to the vaginal ring.

Three events assessed by the Investigator as severe (Grade 3) in intensity were reported during the trial, two in the observation period and one in the ring intervention period. In Group A, a woman experienced abdominal pain for 2 days during the observation period, and another participant, also in Group A, developed appendicitis during the ring intervention period which was also reported as an SAE (described above). One participant in Group B developed cystitis during the observation period which resolved after 11 days on treatment.

Vaginal ring removal due to an AE during the ring intervention period occurred in three participants in Group A, with the rings being permanently removed after 42 to 62 days of use: two participants experienced vaginal candidiasis (assessed by the Investigator as moderate in severity and probably not ring-related) and one participant presented with abdominal tenderness/lower abdominal pain (assessed by the Investigator as mild in severity and possibly ring-related). All three participants completed all scheduled trial visits, received treatment for these events which had resolved by the end of the trial, and were included in the final analysis. The occurrence of vaginal candidiasis and lower abdominal pain was comparable between the ring intervention and observation periods with 18 and 17 participants, respectively, who experienced vaginal candidiasis, and five and three participants, respectively, who reported lower abdominal pain. Abdominal tenderness was experienced by one participant (described above).

Two additional participants in Group B had AEs that resulted in temporary ring removal during the ring intervention period; both participants completed the trial: one participant presented with mild genital pruritus and the ring was removed for 5 days. The second participant had a moderate vaginal laceration (6 mm), that could also be due to non-ring related trauma events such as coitally associated or other object insertions, and the ring was removed for 7 days. Both events were assessed by the Investigator as not related to ring use and resolved spontaneously.

#### Pelvic and colposcopy examinations

No particular differences in pelvic/speculum and colposcopic findings were observed after enrollment between the ring intervention period (40 abnormalities reported) and the observation period (36 abnormalities reported) for either group. The most commonly occurring abnormalities were abnormal vaginal discharge (14 findings in the observation period and 10 findings in the ring intervention period), followed by erythema (six findings in the observation period and seven findings in the ring intervention period), and abrasion (two findings in the observation period and five findings in the ring intervention period). Most of these findings were assessed by the Investigator as mild in severity. Two findings were regarded as severe (Grade 3) in a participant in Group A, who experienced an abrasion on the cervix (5–10 mm); the event occurred 4 weeks after enrollment in the ring intervention regimen. The finding had resolved spontaneously 1 week later and was assessed by the Investigator as not related to ring use. The same participant was again reported with a severe abrasion on the cervix, similar to the first one, 8 weeks post-enrollment which also resolved spontaneously after 1 week. In the time interval between the reporting of these two severe (Grade 3) colposcopy findings, the participant was reported with mild coital bleeding that was probably due to trauma to a cervical ectopy scar caused by sexual intercourse. The event was reported as an AE and the ring was temporarily discontinued, re-introduced after a week, and then permanently removed due to an AE of moderate (Grade 2) vaginal candidiasis which occurred prior to the second severe abrasion finding. The participant received treatment for the event of vaginal candidiasis.

With the exception of seven abnormalities, all post-enrollment pelvic/speculum and colposcopy findings (69/76; 90.8%) were regarded as unrelated to ring use. Six findings, which included mild to moderate cases of abnormal vaginal discharge, ecchymosis, erythema, tenderness on bimanual palpation, and laceration, were regarded as possibly related to the placebo vaginal ring; and one instance of moderate abrasion was considered to be probably related to the vaginal ring. With one exception, all findings had resolved by the next visit.

#### Sexually transmitted infections and bacterial vaginosis

Overall, the number of participants who had positive diagnostic test results for chlamydia, trichomonas and bacterial vaginosis (using Amsel criteria) were somewhat higher during the observation period than the ring intervention period; the difference in bacterial vaginitis did not reach statistical significance. For gonorrhoea, the opposite was observed (Table 4).

**Table 4:** Incidence of sexually transmitted infections and bacterial vaginosis.

Assessment	Ring Intervention Phase			Observation Phase			p-value
	Group A	Group B	Total	Group A	Group B	Total	Total
	N = 87	N = 71	N = 158	N = 87	N = 71	N = 158	n (%)
	n (%)	n (%)	n (%)	n (%)	n (%)	n (%)	
Bacterial Vaginosis	19 (22%)	1 (1%)	20 (13%)	3 (4%)	25 (30%)	28 (17%)	0.2482
Chlamydia	5 (6%)	3 (4%)	8 (5%)	8 (10%)	7 (9%)	15 (9%)	0.1444
Gonorrhea	5 (6%)	2 (3%)	7 (4%)	1 (1%)	2 (2%)	3 (2%)	0.2059
Trichomonas	9 (10%)	0 (0%)	9 (6%)	2 (3%)	10 (12%)	12 (7%)	0.5127

None of the test results showed a statistically significant difference at the 5% level between the ring and observational period (Table 4). Of 162 participants who were tested for STIs and bacterial vaginosis at the crossover visit, 19 (11.7%) participants had positive diagnostic test results of whom nine participants with bacterial vaginosis (using Amsel criteria), seven participants with chlamydia, five participants with gonorrhea and one participant with trichomonas (data not shown). At the final trial visit, 144 participants underwent diagnostic testing for STIs and bacterial vaginosis, and 18 (12.5%) participants tested positive of whom nine participants with bacterial vaginosis, eight participants with chlamydia, two participants with gonorrhea and three participants with trichomonas.

#### HIV and pregnancy

Three participants tested HIV-positive during the course of the trial at the research centre in Durban: two participants in Group A tested positive at the crossover visit, and one participant in Group B tested positive at the end of the ring intervention period at the final trial visit. As this was a placebo safety and acceptability trial, HIV testing was only required at enrollment, and at the crossover and exit visits, or at the discretion of the Investigator. Since no retrospective testing was done in the trial, it is not clear when exactly the HIV infections in these three participants occurred. The results of the rapid HIV test performed at screening were non-reactive (negative) for these participants. The participants were offered appropriate antiretroviral therapy and HIV-related care.

Pregnancy was reported for one participant in Group A who, after completing all scheduled visits, had a positive urine pregnancy test result at the final trial visit (at the end of the observation period). This participant reported using an oral contraceptive Triphasil® (ethinyl estradiol/levonorgestrel).

## Discussion

The use of a placebo vaginal ring, worn continuously for 12 weeks, was assessed as safe and acceptable to women in Tanzania and South Africa. No clinical safety concerns were identified for any of the safety variables assessed during the trial.

No SAEs were reported that were considered related to the use of the placebo vaginal ring. Four participants (2.4%) experienced AEs during the intervention period that were assessed as probably related to vaginal ring use (vaginal odor, coital bleeding, vulvovaginal discomfort, and genital discomfort, each in a single participant), and 10% had possibly related events, including bacterial vaginitis (2%). The incidence of possibly related bacterial vaginitis was lower than that reported with NuvaRing® in three separate studies (4–6%) [15–18]. The incidence of bacterial vaginitis, regardless of causality, was notably higher in the observation period compared to the ring intervention period (23 and 15 participants, respectively), but did not reach statistical significance.

For three participants, the placebo vaginal ring was permanently removed, and for two participants it was temporarily removed, because of AEs. All five participants completed the trial and were included in the final analysis.

To avoid a possible carry-over effect in the trial between the two regimens, specifically during crossing over from the ring regimen to the observational regimen, participants had to show no signs or symptoms of any genital infections or findings on the pelvic/speculum and colposcopy examination at the crossover visit. Participants who presented with findings at this visit were treated and had to be asymptomatic or negative for findings before they were allowed to cross over to the next regimen.

The number of participants who tested positive for HIV in the trial was 6% (3/50) compared to 2% (3/140) in the placebo group during the double-blind, randomized, placebo-controlled trial, conducted over 12.5 months at ten research centers in Kenya, Malawi, Tanzania and South Africa. [19]. One of the three women who seroconverted during the IPM 011 trial tested positive for trichomonas infection at the screening visit, and another was reported with bacterial vaginosis at the crossover visit. Genital infections may increase the risk of HIV infection due to breaks in the mucosal epithelial barrier of the vagina or cervix, and increase the number of target cells in the submucosa due to local inflammation [20].

The rates of STIs and bacterial vaginosis reported in this trial at screening were lower for bacterial vaginosis (using Amsel criteria) (19%) and higher for trichomonas (11%), chlamydia (7%), and gonorrhea (3%) than the combined baseline results of four microbicide trials conducted in West Africa and India (38% for bacterial vaginosis, 6% for trichomonas, 4% for chlamydia, and 2% for gonorrhea) [21].

The social and behavioral outcomes from this trial previously reported [11] suggested that the vaginal ring was highly acceptable to trial participants, the vaginal ring was found easy and convenient to use and appeared highly promising for continuous topical release of antiretroviral-based microbicides [11]. High levels of vaginal ring use were reported by participants, with about 80% of women reporting that they never removed the vaginal ring for the entire 12-week ring use period, and more than 95% of participants reporting that they kept the ring inserted every day for at least 12 hours [12]. Approximately 20% of participants reported vaginal ring removals and/or expulsions and most were associated with menstruation and concerns about hygiene during menses [12], indicating a need for enhanced adherence counseling.

## Conclusions

The silicone elastomer vaginal ring, which is intended for use as a microbicide delivery method for the prevention of HIV-1 infection, was previously reported as well tolerated during the trial [11, 12] with no safety concerns observed. Based on the incidence of AEs, abnormal pelvic/speculum and colposcopy findings as well as STI and bacterial vaginosis results, there was no evidence of any major clinical difference in safety between the 3-month vaginal ring use and the observational safety regimens of the trial.

## Supporting information

S1 Fig Consort checklist. (PDF)

S1 File Study protocol. (PDF)

S1 Table Data points for baseline demographics. (PDF)

S2 Table Data points for sexually transmitted infections and bacterial vaginosis. (PDF)

S3 Table Summary of adverse event occurrence rates by center. (PDF)

S4 Table Summary of adverse event occurrence rates by sequence assignment. (PDF)

S5 Table Summary of adverse event occurrence rates by trial phase. (PDF)
