# Supplementary material for: Safety of a silicone elastomer vaginal ring as potential microbicide delivery method in African women: A Phase 1 randomized trial
Source: PLoS One. 2018 May 29;13(5):e0196904. doi: 10.1371/journal.pone.0196904 (PMC5973569; doi:10.1371/journal.pone.0196904)
Supplement: S1 File — (PDF) [file pone.0196904.s002.pdf]

IPM 011 PROTOCOL AMENDMENT 5

A SAFETY AND ACCEPTABILITY STUDY OF A VAGINAL RING MICROBICIDE DELIVERY METHOD FOR THE PREVENTION OF HIV INFECTION IN WOMEN

International Partnership for Microbicides
8401 Colesville Road
Suite 200
Silver Spring, MD 20910 USA

Final Version 2.0
24 November 2008

PARTICIPATING SITES AND INVESTIGATOR INFORMATION

International Centre for Reproductive Health, Mombasa, Kenya

Principal Investigator:	Dr. Stanley Luchters
Co-Investigators:	Dr. Evans Malava
	Dr. Jennifer Othigo
	Dr. Kishor Mandaliya
	Prof. Marleen Temmerman
	Dr. Zoella Pasta

Kilimanjaro Christian Medical Center, Moshi, Tanzania

Principal Investigator:	Dr. Gilead Masenga
Co-Investigators:	Dr. Sarah Chiduo
	Prof. Saidi Kapiga (Harvard School of Public Health)
	Dr. Noel Sam
	Dr. Charles Mmbando

Desmond Tutu HIV Foundation, Nyanga, Cape Town, South Africa

Principal Investigator:	Prof. Linda-Gail Bekker
Co-Investigators:	Dr. Danielle Crida
	Dr. Llwellyn Fleurs
	Dr. Jennifer Pitt
	Dr. Surita Roux
	Dr. Andrew Scheibe

Reproductive Health Research Unit, Johannesburg, South Africa

Principal Investigator:	Dr. Jocelyn Moyes
Co-Investigators:	Dr. Claire von Mollendorf
	Prof. Helen Rees
	Dr. Lucia Hans

South African Medical Research Council – Durban, South Africa

Principal Investigator:	Dr. Avashri Naidoo
Co-Investigators:	Prof. Gita Ramjee
	Dr. Roshini Govinden
	Dr. Shayhana Ganesh

SPONSOR CONTACT INFORMATION

**Sponsor Contact**	Annaléne Nel, MBChB, PhD
Office Phone:	+27 21 860 2300
Office Fax:	+27 21 860 2308
Office E-mail:	anel@ipm-microbicides.org
Mailing Address:	International Partnership for Microbicides<br>Zomerlust Estate<br>PricewaterhouseCoopers Building<br>Bergriver Boulevard<br>Paarl, 7646<br>SOUTH AFRICA

**Sponsor Medical Monitor**	Katherine Young, MBBCh, or designee
Office Phone:	+27 21 860 2303
Mobile Number:	+27 79 881 9819
Office Fax:	+27 21 860 2308
Office E-mail:	kyoung@ipm-microbicides.org.za
Mailing Address:	International Partnership for Microbicides<br>Zomerlust Estate<br>PricewaterhouseCoopers Building<br>Bergriver Boulevard<br>Paarl, 7646<br>SOUTH AFRICA

SIGNATURE PAGE

IPM 011

A SAFETY AND ACCEPTABILITY STUDY OF A VAGINAL RING MICROBICIDE DELIVERY METHOD FOR THE PREVENTION OF HIV INFECTION IN WOMEN

I have read this protocol and appendices and agree to conduct the trial as stipulated and in compliance with the principles of the World Medical Association Declaration of Helsinki, the International Conference on Harmonisation (ICH) Good Clinical Practice (GCP) guidelines, and all applicable regulations and guidelines.

______________________________	______________________________
Investigator Signature	Date
______________________________	______________________________
Investigator Name (Printed)	Investigative Site Name

On behalf of the International Partnership for Microbicides, I confirm that the sponsor will comply with all obligations as detailed in all applicable regulations and guidelines. I will ensure that the investigator is informed of all relevant information that becomes available during the conduct of this trial.

______________________________	______________________________
Chief Medical Officer Signature	Date

Annaléne Nel, MBChB, PhD
Chief Medical Officer Name (Printed)

TABLE OF CONTENTS

Page

ACRONYMS 9

PROTOCOL SYNOPSIS 10

1.1 *INTRODUCTION* *14*
- **1.2 *Background*** *14*
- **1.3 Rationale for Protocol IPM 011** 15

2.0 STUDY OBJECTIVE 16

3.1 OVERALL STUDY DESIGN 16
- **3.2 *Study Design*** *16*
- **3.3 *Study Duration*** *21*
- **3.4 Study Population** 21
  - **3.4.1 Inclusion Criteria** 21
  - **3.4.2 Exclusion Criteria** 22

4.1 *STUDY VISITS* *23*
- **4.2 *Screening Visit*** *23*
- **4.3 Visit 1 = ENROLLMENT VISIT** 25
  - **4.3.1 Pre-Enrollment Procedures** 25
  - **4.3.2 Enrollment Procedures** 25
    - **4.3.2.1 Group A: Vaginal Ring Regimen** 26
    - **4.3.2.2 Group B: Observational Safety Regimen** 26
- **4.4 Visits 2, 3, & 4 = 2, 4, & 8 Weeks Post-Enrollment Visit** 26
  - **4.4.1 Group A: Vaginal Ring Regimen** 26
  - **4.4.2 Group B: Observational Safety Regimen** 27
- **4.5 Visit 5 = 12 Weeks Post-Enrollment Visit & CROSSOVER VISIT** 27
  - **4.5.1 Group A: Vaginal Ring to Observational Safety Regimen** 27
  - **4.5.2 Group B: Observational Safety to Vaginal Ring Regimen** 28
- **4.6 Visit 5.1 = 2 Weeks Post-Visit 5 & CROSSOVER VISIT** 29
  - **4.6.1 Group A: Vaginal Ring to Observational Saefty Regimen** 29
  - **4.6.2 Group B: Observational Safety to Vaginal Ring Regimen** 29
- **4.7 Visits 6, 7, & 8 = 2, 4, & 8 Weeks Post-Crossover Visit** 30
  - **4.7.1 Group A: Observational Safety Regimen** 30
  - **4.7.2 Group B: Vaginal Ring Regimen** 31
- **4.8 Visit 9 = 12 Weeks Post-Crossover Visit & LAST STUDY VISIT** 31
  - **4.8.1 Group A: Observational Safety Regimen** 31
  - **4.8.2 Group B: Vaginal Ring Regimen** 32
- **4.9 Unscheduled Visits** 32
- **4.10 Missed & Late Visits** 33
- **4.11 Early Discontinuation Visit** 33
- **4.12 Premature Discontinuation of the Study** 34

Page

5.1 STUDY PROCEDURE DETAILS 34
5.2 Informed Consent 34
5.3 HIV Counseling *35*
5.3.1 HIV Pre- and Post-Test Counseling 35
5.3.2 HIV/STI Risk Reduction Counseling 36
5.4 HIV Testing & Management 36
5.5 Contraceptive Counseling 37
5.6 Pregnancy Testing and Management 38
5.7 Medical History and Physical Examination 38
5.8 Pelvic/Speculum Examination 38
5.9 Colposcopy *39*
5.10 STI Testing *39*
5.11 Pap Smear *40*
5.12 Acceptability Questionnaire 40
5.13 Vaginal Ring Insertion & Removal 41
5.14 Vaginal Ring Adherence Counseling 42
5.15 Method of Treatment Assignment 42
5.16 Qualitative Data Component 42
5.17 Reimbursement *43*
5.18 Study Operations Manual 43

6.1 INVESTIGATIONAL PRODUCT 43
6.2 Investigational Product Composition 43
6.3 Investigational Product Storage 44
6.4 Investigational Product Administration 44
6.5 Investigational Product Expulsion or Loss 44
6.6 Investigational Product Accountability 44
6.7 Concomitant Medications & Products 44

7.1 ADVERSE EVENTS *45*
7.2 Definition *45*
7.3 Assessment of Adverse Event Severity 46
7.4 Relationship to Investigational Product 47
7.5 Serious Adverse Events 48
7.5.1 Serious Adverse Event Definition 48
7.5.2 Serious Adverse Event Contact Information 49
7.5.3 Sponsor Notification of Unexpected SAEs to Regulatory Agencies 49
7.5.4 Site Notification of SAEs to Local Ethics Committee or Local Health or Regulatory Authorities 50

8.1 ***DATA MANAGEMENT*** *50*

TABLE OF CONTENTS (Continued)

TABLE OF CONTENTS (Continued)

Page

8.2 Data Handling at Study Sites 50
8.3 Source Data Verification 50

9.1 STATISTICAL METHODS 50
9.2 General Design 50
9.3 Sample Size 52
9.4 Analyses 52
9.4.1 Analysis of Safety Measures 52
9.4.2 Analysis of Acceptability Measures 53
9.4.3 Adherence Analysis 54
9.4.4 Condom Use and Sexual Behavior 55
9.4.5 Handling of Missing Data and Dropouts 55

10.1 INVESTIGATOR REQUIREMENTS 56
10.2 Study Initiation 56
10.3 Institutional Review Board or Institutional Ethics Committee Approval 57
10.3 Study Monitoring and Audits 57
10.4 Case Report Forms 58
10.5 Disclosure of Data 58
10.6 Record Retention 58

11.1 ETHICAL CONSIDERATIONS 59
11.2 Ethical Review 59
11.3 Social Harms 59

12.0 PUBLICATION 59

13.0 REFERENCES 60

APPENDIX A: Schedule of Clinical Procedures Group A 61

APPENDIX B: Schedule of Clinical Procedures Group B 62

APPENDIX C: HIV Testing Algorithm 63

APPENDIX D: Vaginal Ring Insertion & Removal Instructions 64

APPENDIX E: Division of AIDS (DAIDS) Table for Grading Severity of Adult Adverse Experiences for the HIV Prevention Trials Network 67

APPENDIX F: Clinical Management of Genital Diagnoses 75

APPENDIX G: Sample Screening Consent Form Template 77

TABLE OF CONTENTS (Continued)

Page

APPENDIX H: Sample Study Consent Form Template 81

APPENDIX I: Sample Consent Form Template Female Focus Group 90

APPENDIX J: Sample Consent Form Template Male Individual Interview 94

ACRONYMS

AE	Adverse Event
AIDS	Acquired Immunodeficiency Syndrome
AIDS-defining illness	A condition, such as an opportunistic infection, that is included in the 1993 Revised Classification System for HIV Infection and Expanded Surveillance Case Definition for AIDS among Adolescents and Adults
ARV	Antiretroviral
ASC-H	Atypical Squamous Cells - cannot exclude HSIL
BV	Bacterial Vaginosis
CDC	U.S. Centers for Disease Control and Prevention
CONRAD	Contraceptive Research & Development
CRF	Case Report Form
EIA	Enzyme Immunoassay
FDA	Food & Drug Administration (U.S.)
GCP	Good Clinical Practice
HIV-1	Human Immunodeficiency Virus-1
HSIL	High grade Squamous Intraepithelial Lesion
IB	Investigator's Brochure
ICF	Informed Consent Form
ICH	International Conference on Harmonisation
IEC	Institutional Ethics Committee
IND	Investigational New Drug
IPM	International Partnership for Microbicides
IRB	Institutional Review Board
LSIL	Low grade Squamous Intraepithelial Lesion
NRTI	Nucleoside Reverse Transcriptase Inhibitor
NNRTI	Non-nucleoside Reverse Transcriptase Inhibitor
PMTCT	Prevention of Mother-to-Child Transmission of HIV
R&D	Research and Development
SAE	Serious Adverse Event
SCR-ID	Screening Identification Number
SID	Subject Identification Number
STI	Sexually Transmitted Infection
US	United States
WHO	World Health Organization

PROTOCOL SYNOPSIS

A SAFETY AND ACCEPTABILITY STUDY OF A VAGINAL RING MICROBICIDE DELIVERY METHOD FOR THE PREVENTION OF HIV INFECTION IN WOMEN

BACKGROUND: To date, candidate vaginal microbicides have been formulated predominantly as gels, films, and suppositories. Multiple safety and efficacy trials with various microbicides are currently underway, most of which are designed to deliver the microbicide in gel form via a single-use vaginal applicator. However data suggest that compliance may be a critical factor in microbicide efficacy due to issues of gel acceptability and the fact that most gels are coitally dependent. More recently, vaginal rings have been proposed as alternative microbicide delivery methods that may have advantages over other formulations, since use of a ring can circumvent difficulties associated with daily or coitally dependent application of a gel.

PRIMARY OBJECTIVE: To assess the safety and acceptability of a silicone elastomer vaginal ring intended as a microbicide delivery method for the prevention of HIV infection when inserted in place for a 12 week period in healthy sexually active women.

DESIGN: IPM 011 is an open-label crossover study conducted at 5 sites in Kenya, South Africa, and Tanzania among 230 healthy sexually active women to assess the safety and acceptability of a silicone elastomer vaginal ring (containing no drug) intended as a microbicide delivery method for the prevention of HIV infection. Upon enrollment at each site, subjects will be randomly assigned in a 1:1 ratio to one of two study groups, Group A and Group B. Both groups will participate in two regimens: Vaginal Ring and Observational Safety (with no vaginal ring) with each regimen 12 weeks in duration.

Group A will participate in the Vaginal Ring regimen for the first 12 weeks of the study, self-insert a vaginal ring at enrollment (Visit 1) to be worn for 12 continuous weeks, and be followed 2, 4, 8, and 12 weeks post-enrollment ring insertion (Visits 2-5) to monitor safety and acceptability. After the first 12 week period is completed, Group A will remove the vaginal ring, and if asymptomatic for genital infections and negative for findings on the pelvic/speculum examination with coloposcopy, will cross over into the Observational Safety regimen at Visit 5 for the last 12 weeks of the study, and return to the clinic 2, 4, 8 and 12 weeks post-crossover visit (Visits 6-9) for follow-up observational safety only. If the subject has any genital symptomatology or findings on the pelvic/speculum examination with colposcopy after the first 12 week period is completed,

she will not cross over into the Observational Safety regimen but she will be treated as appropriate and will return to the clinic within 2 weeks (Visit 5.1). At that time, if the subject is asymptomatic for genital infections and negative for findings on the pelvic/speculum examination with colposcopy, she will cross over into the Observational Safety regimen and return to clinic 2, 4, 8 and 12 weeks post-Visit 5.1 crossover timepoint. If she continues to have any genital symptomatology or findings on the pelvic/speculum examination with colposcopy, she will conclude study participation and be followed until her condition is resolved or stabilized.

Group B will participate in the Observational Safety regimen for the first 12 weeks of the study and after enrollment (Visit 1) will be followed 2, 4, 8, and 12 weeks post-enrollment (Visits 2-5) for observational safety only. After the first 12 week period is completed, if asymptomatic for genital infections and negative for findings on the pelvic/speculum examination with coloposcopy, Group B will cross over into the Vaginal Ring regimen at Visit 5 for the last 12 weeks of the study, self-insert a vaginal ring to be worn for 12 continuous weeks, and be followed 2, 4, 8 and 12 weeks post-crossover ring insertion (Visits 6-9) to monitor safety and acceptability. If the subject has any genital symptomatology or findings on the pelvic/speculum examination with colposcopy after the first 12 week period is completed, she will not cross over into the Vaginal Ring regimen but she will be treated as appropriate and will return to the clinic within 2 weeks (Visit 5.1). At that time, if the subject is asymptomatic for genital infections and negative for findings on the pelvic/speculum examination with colposcopy, she will cross over into the Vaginal Ring regimen and return to clinic 2, 4, 8 and 12 weeks post-Visit 5.1 crossover timepoint. If she continues to have any genital symptomatology or findings on the pelvic/speculum examination with colposcopy, she will conclude study participation and be followed until her condition is resolved or stabilized.

Both groups will be followed on-study for a total of 24 weeks (i.e., two 12-week regimens) or 26 weeks if 2 additional weeks are required in the event the crossover visit is delayed due to genital symptomatology or findings on pelvic/speculum examination with colposcopy.

		**CROSSOVER**	
	**FIRST 12 WEEKS**		**LAST 12 WEEKS**
Group A	Vaginal Ring	→	Observational Safety
Group B	Observational Safety	→	Vaginal Ring

Approximately 20 women at each site who have completed the Vaginal Ring regimen in IPM 011 at each of the clinical sites may participate in separate focus group discussions within one month after completing the Vaginal Ring regimen depending on the accrual plan and exit schedule. Approximately 20 men who are partners of women who have completed the Vaginal Ring regimen in IPM 011 at each of the clinical sites may participate in individual interviews within one month after the women have completed the Vaginal Ring regimen depending on the accrual plan and exit schedule. The purpose of these discussions and interviews is to provide additional information on acceptability of the vaginal ring, in order to strengthen interpretation of findings from the acceptability questionnaire administered during the study.

STUDY POPULATION: Healthy sexually active women 18-35 years of age who understand the study and can provide informed consent.

SAMPLE SIZE: 230 women (between 20 and 60 per site) will be enrolled using competitive enrollment.

REGIMEN: Potential subjects who consent will be invited to screen for the study. All subjects who consent to participate in the trial, meet specified inclusion/exclusion criteria, have normal findings based upon a physical and pelvic/speculum examination with colposcopy and medical history, and have negative pregnancy and HIV tests will be invited to enroll in the study. Eligible women will be randomly assigned in a 1:1 ratio to one of two study groups, Group A and Group B.

During the 24 week study period, all subjects (Groups A and B) will undergo a general physical examination at screening, and a pelvic/speculum examination at screening, enrollment, 2 and 8 weeks post-enrollment, crossover, 2 and 8 weeks post-crossover and the last study visit. At enrollment, the crossover visit, and the last study visit, all subjects will also undergo a colposcopy examination. STI testing will be performed at screening, the crossover visit and the last study visit. A Pap smear will be done at screening. HIV/STI risk reduction counseling including condom dispensing and contraceptive counseling will be provided at all study visits including screening. Vaginal ring adherence counseling will be provided at the time of ring insertion and at every follow-up visit during the Vaginal Ring regimen only, i.e., for Group A, this will occur at enrollment and 2, 4, and 8 weeks post-enrollment ring insertion; and for Group B, this will occur at the crossover visit and 2, 4, and 8 weeks post-crossover ring insertion visit. HIV testing with pre- and post-test counseling will be conducted at screening, the crossover visit and the last study visit. In addition to screening, enrollment, the crossover visit

and the last study visit, pregnancy testing will only be conducted in the event the subject misses a menstrual period during the study. An acceptability questionnaire will be administered during the Vaginal Ring regimen, i.e., for Group A, this will occur at enrollment and 4, 8, 12 weeks post-enrollment ring insertion and the last study visit; and for Group B, this will occur at enrollment, the crossover visit and 4, 8 and 12 weeks post-crossover ring insertion visit. Adverse events, including vaginal complaints, and concomitant medications will be assessed at every visit.

STUDY DURATION: The maximum allowable time between screening and enrollment per subject is 30 days. Following enrollment into the study, each subject will be followed for a total of up to 26 weeks (i.e., two 12-week regimens and 2 additional weeks if the crossover visit is delayed due to genital symptomatology or findings on pelvic/speculum examination with colposcopy). It is anticipated full enrollment will be completed in 12 weeks per site for a total of up to 38 weeks (approximately 9 months) study duration.

1.1 INTRODUCTION

1.2 Background

The AIDS epidemic continues to exact a devastating toll on the health, economic and political infrastructure, and social fabric of communities worldwide. During 2005, almost 4.1 million people became newly infected with HIV-1, bringing the total number of people living with HIV to an estimated 38.6 million. In the same year almost 2.8 million died from AIDS, raising the global death toll to over 20 million since the first cases of AIDS were identified in 1981 (1). Over 95 percent of new infections are occurring in developing countries where there is little access to the treatment options that have prolonged life for HIV-positive persons in industrialized countries. Developing safe and effective HIV prevention technologies that can be made easily accessible in developing countries is thus an urgent public health priority.

Epidemiologic data published in the latest UNAIDS report show that women and girls bear a severe and increasingly heavy burden in the HIV epidemic. In Eastern Europe and Central Asia, an estimated 420,000 women aged 15 years and older were living with HIV in 2005, which is one-third more than the 310,000 in 2003. In China women accounted for 39% of new HIV cases reported in 2004, compared to 25% of the cases reported in 2002. In Cambodia 47% of adults living with HIV in 2003 were female, compared to 37% in 1998. In sub-Saharan Africa, women comprised 57% of HIV positive adults, and among young people (aged 15-24 years) the ratio of infection had risen to three young women for every infected young man. Similar statistics were reported for the Caribbean, where 51% of adults living with HIV are female. Young women in their late teens once again represented a particularly vulnerable subset of the population due to a combination of biological and sociological factors. In Trinidad, for example, young girls between the ages of 15-19 years were up to six times more likely to be HIV infected as their male counterparts (1).

Unprotected heterosexual intercourse is currently the leading source of HIV incidence amongst females. Correct and consistent use of latex condoms is one proven method of preventing HIV transmission; however, condoms are widely regarded as inadequate prevention options for women, who are often unable to negotiate condom use with their partners for fear of abuse or accusations of infidelity. Additionally, women who are having sex with men in exchange for gifts or money may be reluctant to use condoms if the men are willing to pay more for sex without a condom. The female condom has been marketed as an alternative barrier method, but this device is relatively costly and requires a certain level of skill, as well as acceptance by the male partner. Developing HIV prevention options that women can use with or without their partner’s knowledge is a pressing global concern given the rapidly growing HIV infection rate among women and the absence of an effective vaccine. Topical microbicides that can be self-administered to the vagina are one such promising alternative.

To date, candidate vaginal microbicides have been formulated predominantly as gels, films, and suppositories. Multiple safety and efficacy trials with various microbicides are currently underway, most of which are designed to deliver the microbicide in gel form via a single-use vaginal applicator. However data suggest that compliance may be a critical factor in microbicide efficacy due to issues of gel acceptability and the fact that most gels are coitally dependent. More recently, vaginal rings have been proposed as alternative microbicide delivery methods that may have advantages over other formulations, since use of a ring can circumvent difficulties associated with daily or coitally dependent application of a microbicide gel.

1.2 Rationale for Protocol IPM 011

While safety and efficacy are critical factors in the development of vaginal microbicides, in order for these products to have an impact on HIV transmission rates, they must also employ delivery methods that are acceptable to potential users.

Vaginal rings have already been developed and approved as delivery methods for medications such as Femring®, a hormone replacement product approved in June 2003 by the United States (U.S.) Food and Drug Administration (FDA) which treats menopause-induced vasomotor symptoms (e.g., hot flashes) and symptoms of vulvar and vaginal atrophy (e.g., dryness). Femring® is a silicone elastomer ring which contains estradiol acetate and is approved for 3 continuous months of use. Based on clinical studies of Femring®, the silicone elastomer ring itself appears to be safe as contraindications appear to be relative to estrogen use (2). Moreover, a recent acceptability study of the silicone elastomer ring used in Femring® (but containing no drug) among postmenopausal women in the U.S. demonstrated very high acceptability and ease of use (3).

The favorable response from postmenopausal women to a vaginal ring drug delivery method is promising. However, additional information is needed on the acceptability of the vaginal ring as a drug delivery method among pre-menopausal women, and also among women in geographical regions other than the U.S. IPM 011 is designed to assess the acceptability of the same type of silicone elastomer vaginal ring used in Femring® (but containing no drug) used over a 12 week period among healthy sexually active women in Africa. The study will be an open-label crossover study conducted at 5 sites in Kenya, South Africa, and Tanzania among 230 healthy sexually active women to assess the safety and acceptability of a silicone elastomer vaginal ring (containing no drug) intended as a microbicide delivery method for the prevention of HIV infection.

Since this study will be open-label and subjects will not be randomized to a placebo control, a crossover study design will be employed to evaluate safety. In the crossover design, each subject will be evaluated for 12 weeks with the vaginal ring inserted followed by another 12 weeks without the ring, or vice versa. Vaginal “events” with the ring inserted will be compared to events occurring during the period without the ring in each subject as well as with other women in the study for the respective study periods. This will improve the ability to evaluate safety in each woman since vaginal problems

(e.g., bacterial vaginosis, yeast infections, urinary tract infections, and vaginal symptoms) occur very commonly in "healthy" populations, even in the absence of a vaginal ring.

2.0 STUDY OBJECTIVE

To assess the safety and acceptability of a silicone elastomer vaginal ring intended as a microbicide delivery method for the prevention of HIV infection when inserted in place for a 12 week period in healthy sexually active women.

3.1 OVERALL STUDY DESIGN

3.2 Study Design

IPM 011 is an open-label crossover study conducted in Kenya, South Africa, and Tanzania among 230 healthy sexually active women to assess the safety and acceptability of a silicone elastomer vaginal ring (containing no drug) intended as a microbicide delivery method for the prevention of HIV infection.

Not including screening, all subjects will have 9-10 scheduled clinic visits as follows:

- Visit 1: **ENROLLMENT VISIT**
- Visit 2: 2 weeks post-enrollment visit
- Visit 3: 4 weeks post-enrollment visit
- Visit 4: 8 weeks post-enrollment visit
- Visit 5: 12 weeks post-enrollment **AND CROSSOVER VISIT**
- **Visit 5.1: 2 weeks post-Visit 5 AND CROSSOVER VISIT (ONLY for those with genital symptomatology or abnormal findings on pelvic/speculum examination with colposcopy at Visit 5)**
- Visit 6: 2 weeks post-crossover visit
- Visit 7: 4 weeks post-crossover visit
- Visit 8: 8 weeks post-crossover visit
- Visit 9: 12 weeks post-crossover visit **AND LAST STUDY VISIT**

With the exception of Visits 1 and 5.1, all visits have a study window of ±5 days. The overall study design is described below. For more details, refer to Section 4.0 Study Visits.

<u>At the Screening Visit</u>:

- Screening and study explained
- Written consent obtained
- Screening Identification Number (SCR-ID) assigned
- Collection of demographic information, relevant medical history, concomitant medication, and locator and menses information

- Inclusion and exclusion criteria assessment
- HIV/STI risk-reduction counseling and condom distribution
- Pre- and post-test HIV counseling
- Contraceptive counseling
- HIV rapid test
- Urine pregnancy test
- General physical examination
- Pelvic/speculum examination with collection of cervico-vaginal specimens for STI testing and a Pap smear
- Enrollment visit scheduled within 30 days based on continued study eligibility

<u>At the Enrollment Visit (Visit 1):</u>

- Pre-Enrollment Procedures conducted as follows:
  - o Collection of medical problems and concomitant medications since the last visit, as well as locator and menses information
  - o HIV/STI risk-reduction counseling (including condom distribution)
  - o Contraceptive counseling
  - o Urine pregnancy test
  - o Pelvic/speculum examination with colposcopy
- If the woman meets all inclusion criteria and none of the exclusion criteria, written informed consent obtained again and commencement of enrollment
- **Upon enrollment, random assignment of Subject Identification Number (SID) in 1:1 ratio to Group A or Group B**

<u>**GROUP A: Vaginal Ring First 12 Weeks & Observational Safety Last 12 Weeks**</u>

- **Visit 1 (ENROLLMENT VISIT)** – After SID assignment:
  - o Acceptability questionnaire administered
  - o Vaginal ring self-insertion
  - o Brief digital exam to ensure proper ring placement
  - o Vaginal ring adherence counseling

- **Visits 2, 3, & 4 (2, 4, and 8 weeks post-enrollment visit)**
  - o Collection of adverse events/concomitant medications since last visit and locator and menses information
  - o HIV/STI risk-reduction counseling and condom distribution
  - o Contraceptive counseling
  - o Pelvic/speculum examination <u>at Visits 2 and 4 only</u> (subject will remove and re-insert ring with each pelvic examination)
  - o Vaginal ring adherence counseling
  - o Acceptability questionnaire administered <u>at Visits 3 and 4 only</u>

- **Visit 5 (12 weeks post-enrollment and CROSSOVER VISIT)**
  - o Collection of adverse events/concomitant medications since last visit and locator and menses information

  - HIV/STI risk-reduction counseling and condom distribution
  - Pre- and post-test HIV counseling
  - Contraceptive counseling
  - HIV rapid test
  - Urine pregnancy test
  - Vaginal ring removal
  - Pelvic/speculum examination with colposcopy and collection of cervico-vaginal specimens for STI testing
  - Acceptability questionnaire administered
  - Crossover into Observational Safety regimen – NOTE: If any genital symptomatology or findings on the pelvic/speculum examination with colposcopy are noted, DO NOT CROSSOVER. Treat as appropriate and schedule Visit 5.1 in 2 weeks.

- **Visit 5.1 (2 weeks post-Visit 5 AND CROSSOVER VISIT -- ONLY for those with genital symptomatology or abnormal findings on pelvic/speculum examination with colposcopy at Visit 5)**
  - Collection of adverse events/concomitant medications since last visit and locator and menses information
  - HIV/STI risk-reduction counseling and condom distribution
  - Contraceptive counseling
  - Pelvic/speculum examination with colposcopy
  - If asymptomatic for genital infections and negative for findings on the pelvic/speculum examination with colposcopy, crossover into Observational Safety regimen.
  - If continued genital symptomatology or findings on the pelvic/speculum examination with colposcopy, conclude study participation and follow subject until condition is resolved or stabilized

- **Visits 6, 7 & 8 (2, 4, and 8 weeks post-crossover visit)**
  - Collection of adverse events/concomitant medications since last visit and locator and menses information
  - HIV/STI risk-reduction counseling and condom distribution
  - Contraceptive counseling
  - Pelvic/speculum examination <u>at Visits 6 and 8 only</u>

- **Visit 9 (12 weeks post-crossover and LAST STUDY VISIT)**
  - Collection of adverse events/concomitant medications since last visit and menses information
  - HIV/STI risk-reduction counseling and condom distribution
  - Pre- and post-test HIV counseling
  - Contraceptive counseling
  - HIV rapid test
  - Urine pregnancy test

  - Pelvic/speculum examination with colposcopy and collection of cervico-vaginal specimens for STI testing
  - Acceptability questionnaire
  - Study participation concluded

<u>GROUP B: Observational Safety First 12 Weeks & Vaginal Ring Last 12 Weeks</u>

- **Visit 1 (ENROLLMENT VISIT)** – After SID assignment:
  - Acceptability questionnaire administered

- **Visits 2, 3, & 4 (2, 4, and 8 weeks post-enrollment visit)**
  - Collection of adverse events/concomitant medications since last visit and locator and menses information
  - HIV/STI risk-reduction counseling and condom distribution
  - Contraceptive counseling
  - Pelvic/speculum examination <u>at Visits 2 and 4 only</u>

- **Visit 5 (12 weeks post-enrollment and CROSSOVER VISIT)**
  - Collection of adverse events/concomitant medications since last visit and locator and menses information
  - HIV/STI risk-reduction counseling and condom distribution
  - Pre- and post-test HIV counseling
  - Contraceptive counseling
  - HIV rapid test – if positive, DO NOT CROSSOVER INTO VAGINAL RING REGIMEN
  - Urine pregnancy test -- if positive, DO NOT CROSSOVER INTO VAGINAL RING REGIMEN
  - Pelvic/speculum examination with colposcopy and collection of cervico-vaginal specimens for STI testing
  - Crossover into Vaginal Ring regimen – NOTE: If any genital symptomatology or findings on the pelvic/speculum examination with colposcopy are noted, DO NOT CROSSOVER (and DO NOT perform remaining 4 procedures immediately below). Treat as appropriate and schedule Visit 5.1 in 2 weeks.
  - Acceptability questionnaire administered
  - Vaginal ring self-insertion
  - Brief digital exam to ensure proper ring placement
  - Vaginal ring adherence counseling

- **Visit 5.1 (2 weeks post-Visit 5 AND CROSSOVER VISIT -- ONLY for those with genital symptomatology or abnormal findings on pelvic/speculum examination with colposcopy at Visit 5)**
  - Collection of adverse events/concomitant medications since last visit and locator and menses information
  - HIV/STI risk-reduction counseling and condom distribution
  - Contraceptive counseling

    - Pelvic/speculum examination with colposcopy
    - If asymptomatic for genital infections and negative for findings on the pelvic/speculum examination with colposcopy, crossover into the Vaginal Ring regimen and perform the following:
        - Acceptability questionnaire administered
        - Vaginal ring self-insertion
        - Brief digital exam to ensure proper ring placement
        - Vaginal ring adherence counseling
    - If continued genital symptomatology or findings on the pelvic/speculum examination with colposcopy, conclude study participation and follow subject until condition is resolved or stabilized

- **Visits 6, 7 & 8 (2, 4, and 8 weeks post-crossover visit)**
    - Collection of adverse events/concomitant medications since last visit and locator and menses information
    - HIV/STI risk-reduction counseling and condom distribution
    - Contraceptive counseling
    - Pelvic/speculum examination at Visit 6 and 8 only (subject will remove and re-insert ring with each pelvic examination)
    - Vaginal ring adherence counseling
    - Acceptability questionnaire administered at Visits 7 and 8 only

- **Visit 9 (12 weeks post-crossover and LAST STUDY VISIT)**
    - Collection of adverse events/concomitant medications since last visit and menses information
    - HIV/STI risk-reduction counseling and condom distribution
    - Pre- and post-test HIV counseling
    - Contraceptive counseling
    - HIV rapid test
    - Urine pregnancy test
    - Vaginal ring removal
    - Pelvic/speculum examination with colposcopy and collection of cervico-vaginal specimens for STI testing
    - Acceptability questionnaire
    - Study participation concluded

CROSSOVER

	**FIRST 12 WEEKS**		**LAST 12 WEEKS**
Group A	Vaginal Ring	→	Observational Safety
Group B	Observational Safety	→	Vaginal Ring

All adverse events will be monitored until resolution and/or the cause is identified or until the site investigator does not expect any improvement or worsening of condition/symptoms. If an adverse event remains unresolved at the subject's last study visit, a clinical assessment will be made by the site's investigator and the IPM Medical Monitor or designee to determine whether continued follow-up of the adverse event is warranted.

Approximately 20 women at each site who have completed the Vaginal Ring regimen in IPM 011 at each of the clinical sites may participate in separate focus group discussions within one month after completing the Vaginal Ring regimen depending on the accrual plan and exit schedule. Approximately 20 men who are partners of women who have completed the Vaginal Ring regimen in IPM 011 at each of the clinical sites may participate in individual interviews within one month after the women have completed the Vaginal Ring regimen depending on the accrual plan and exit schedule. The purpose of these discussions and interviews is to provide additional information on acceptability of the vaginal ring, in order to strengthen interpretation of findings from the acceptability questionnaire administered during the study. Refer to Section 5.15 for more details.

This study will be conducted in accordance with the ethical principles of the World Medical Association Declaration of Helsinki, the International Conference on Harmonisation (ICH) Good Clinical Practice (GCP) guidelines and any applicable local regulatory requirements (4-5).

3.3 Study Duration

The maximum allowable time between screening and enrollment per subject is 30 days. Each subject will be followed on-study for a total of 24 weeks (i.e., two 12-week regimens) or 26 weeks if 2 additional weeks are required in the event the crossover visit is delayed due to genital symptomatology or findings on pelvic/speculum examination with colposcopy). It is anticipated full enrollment will be completed in 12 weeks per site for a total of up to 38 weeks (approximately 9 months) study duration.

3.4 Study Population

This study will enroll sexually active women 18-35 years of age, who in the opinion of the investigator understand the study and can provide informed consent. 230 subjects will be enrolled.

3.3.1 Inclusion Criteria

Women must meet all of the following criteria to be eligible for study enrollment:

1. Women ≥18 and ≤35 years of age who can give written informed consent; illiterate participants may provide a thumbprint or mark witnessed by a person independent from the study staff;

2. Available for all visits and consent to follow all procedures scheduled for the study;

3. Healthy and self-reported sexually active (defined as one penetrative vaginal coital act per month for the last 3 months prior to enrollment);

4. HIV-negative as determined by a HIV rapid test at time of enrollment;

5. On a stable oral contraceptive regimen or have had an IUD inserted (with no vaginal complaints or gynecological symptoms associated with its use) for at least 3 months prior to enrollment or on long-acting progestins for 6 months prior to enrollment or have undergone surgical sterilization at least 3 months prior to enrollment and willing to continue practicing at least one form of contraception which does not utilize a vaginal barrier method (i.e., vaginal hormonal ring, diaphragm, cervical cap or shield) for the duration of the study or have undergone surgical sterilization at least 3 months prior to enrollment;

6. In the absence of the use of exogenous hormone(s), have a regular menstrual cycle defined as having a minimum of 21 days and a maximum of 36 days between menses;

7. Willing to undergo pelvic/speculum examinations with colposcopy according to the protocol throughout the study;

8. Upon pelvic/speculum examination, the cervix and vagina appear normal as determined by the investigator;

9. Asymptomatic for genital infections and have a normal pelvic/speculum examination with colposcopy at the time of enrollment (if a woman is diagnosed with any STI syndromically or by laboratory test during the screening period, she must receive treatment for at least 72 hours prior to enrollment);

10. Willing to refrain from use of vaginal products or objects including tampons, cotton wool, rags, diaphragms, cervical caps, douches and drying agents within 14 days from enrollment and for the duration of the study;

11. Willing to answer acceptability questionnaires throughout the study;

12. Willing to refrain from participation in any other research study;

13. Willing to provide adequate locator information for study retention purposes and be reachable per local standard procedures (e.g., by home visit or telephone; or via family or close neighbor contacts [confidentiality to be maintained].

3.3.2 Exclusion Criteria

Women who have any of the exclusion criteria below are not eligible:

1. Currently pregnant or last pregnancy within 3 months prior to enrollment;

2. Currently breast-feeding;

3. Participated in any other research study within 30 days prior to enrollment;

4. Symptomatic untreated vaginal infections, e.g., urinary tract or other sexually transmitted infections, or other gynecological symptoms, e.g., vaginal itching, pain, or discharge, within 2 weeks prior to enrollment;

5. Presence of abnormal physical finding on the vulva, vaginal walls or cervix during pelvic/speculum examination and/or colposcopy, such as abrasions, lacerations, petechiae, ulcerations, etc.;

6. History of significant urogenital or uterine prolapse, undiagnosed vaginal bleeding, urethral obstruction;

7. Pap smear result at screening that requires cryotherapy, biopsy, treatment (other than for infection), or further evaluation [this includes any findings of atypical squamous cells of undetermined significance (ASCUS)];

8. Unexplained, undiagnosed abnormal bleeding per vagina, bleeding per vagina during or following vaginal intercourse, or gynecologic surgery within 90 days prior to enrollment;

9. Any history of anaphylaxis or severe allergy resulting in angioedema; or a history of sensitivity/allergy to latex or silicone elastomer;

10. Any serious acute, chronic or progressive disease (e.g., any known history of neoplasm, cancer, insulin-dependent diabetes, cardiac disease, autoimmune disease, HIV, AIDS, or blood dyscrasias), or with signs of cardiac disease, renal failure, or severe malnutrition;

11. Any condition(s) that, in the opinion of the investigator, might interfere with adherence to study requirements or evaluation of the study objectives.

4.1 STUDY VISITS

4.2 Screening Visit

a. Explain screening and study procedures to potential subject.

b. If potential subject agrees to participate, obtain written informed consent (illiterate subjects may provide a thumbprint or mark witnessed and signed by a person independent from study staff).

c. Assign unique Screening Identification number (SCR-ID) to potential subject in sequential order. SCR-ID numbers should never be reassigned. A master log of screening subjects with SCR-ID, demographic and locator information must be maintained and kept in a locked/secure location to track potential subjects who have been screened for the study.

d. Obtain and record demographic information, relevant medical history, concomitant medication (taken within the last 30 days), and locator and menses information. Refer to Section 5.6 for more details about relevant medical history.

e. Conduct preliminary review of inclusion/exclusion criteria with potential subject, e.g., whether sexually active, etc.

f. Provide HIV/STI risk-reduction counseling (including dispensing of condoms), pre- and post-test counseling, and contraceptive counseling. Refer to Sections 5.2.2, 5.2.1, and 5.4, respectively for further details.

g. Obtain an oral swab for HIV rapid testing and a urine sample for pregnancy testing.

h. If either the HIV rapid test or urine pregnancy test is positive, the woman is not eligible to enroll in the study and no further study procedures will be performed. For women who test HIV positive, perform a finger prick (and draw 3 ml venous blood, if necessary) for confirmatory testing, and refer her to local health facilities for social support or other medical services as clinically indicated (refer to Section 5.3 for further details). Refer pregnant women to the local prenatal clinic for support services.

i. If both the HIV rapid test and urine pregnancy test are negative, perform general physical examination. Refer to Section 5.6 for a description of the elements required in the general physical examination.

j. Perform pelvic/speculum examination. If the pelvic/speculum examination indicates abnormal findings, e.g., any symptoms or evidence of genital infections, abrasions, ulcerations, etc., provide or refer for appropriate treatment. The potential subject must be asymptomatic for genital infections and have a normal pelvic/speculum examination with colposcopy at the time of enrollment. ***NOTE:*** *If the potential subject is menstruating at the time of screening, the pelvic/speculum examination with sample collection should be rescheduled for two days after completion of menses.*

k. Collect cervico-vaginal swabs for STI testing and a Pap smear. ***NOTE:*** *If Pap smear results require cryotherapy, biopsy, treatment (other than for infection) or further*

evaluation including any findings of atypical squamous cells of undetermined significance (ASCUS), the subject is ineligible for the study but should be referred for appropriate treatment.

l. Based on continued study eligibility, invite potential subject to return to clinic within 30 days for the enrollment visit.

4.3 Visit 1 = ENROLLMENT VISIT (Within 30 days of Screening)

NOTE: *If potential subject is menstruating at this visit, the entire visit should be rescheduled for two days after completion of menses but must be completed within 30 days of the screening visit.*

4.2.1 Pre-Enrollment Procedures

a. Obtain and record any medical problems and concomitant medication since the last visit. ***NOTE:*** *Record any acute conditions as part of the Relevant Medical History. See Section 5.6.*

b. Obtain locator and menses information.

c. Provide HIV/STI risk-reduction counseling (including dispensing of condoms) and contraceptive counseling. Refer to Sections 5.2.2 and 5.4, respectively for further details.

d. Obtain a urine sample for pregnancy testing.

e. If the urine pregnancy test is positive, the woman is not eligible to enroll in the study and no further study procedures will be performed. Refer pregnant women to the local prenatal clinic for support services.

f. If the urine pregnancy test is negative, perform pelvic/speculum examination with colposcopy. ***NOTE:*** *The potential subject must be asymptomatic for genital infections and have a normal pelvic/speculum examination with colposcopy at the time of enrollment.*

g. If all inclusion criteria and none of the exclusion criteria continue to be met, invite woman to enroll immediately.

4.2.2 Enrollment Procedures

a. If potential subject agrees to enroll in the study, obtain written informed consent again (illiterate subjects may provide a thumbprint or mark witnessed and signed by a person independent from study staff).

b. Assign unique Subject Identification Number (SID) to subject in sequential order. Subjects assigned to Group A will participate in the Vaginal Ring regimen for the first half of the study and the Observational Safety regimen for the second half of the study. Subjects assigned to Group B will participate in the Observational Safety regimen for the first half of the study and the Vaginal Ring regimen for the second half of the study.

4.2.2.1 GROUP A: VAGINAL RING REGIMEN

c. Administer acceptability questionnaire. ***NOTE:*** *The questionnaire must be administered prior to insertion of the vaginal ring.*

d. Dispense one vaginal ring to the subject.

e. Instruct the subject to insert the vaginal ring. Perform brief digital examination to verify the vaginal ring has been properly placed.

f. Provide vaginal ring adherence counseling. Refer to Section 5.13 for further details.

g. Schedule next visit.

4.2.2.2 GROUP B: OBSERVATIONAL SAFETY REGIMEN

c. Administer acceptability questionnaire.

d. Schedule next visit.

4.3 Visits 2, 3, & 4 = 2, 4, & 8 Weeks Post-Enrollment Visit (Window ±5 days)

NOTE: *If subject is menstruating or anticipates menstruation at any visit, the entire visit should be rescheduled for two days after completion of menses but should be completed within the visit window.*

4.3.1 GROUP A: VAGINAL RING REGIMEN

a. Obtain and record any adverse events and concomitant medications since the last visit.

b. Obtain locator and menses information.

c. Provide HIV/STI risk reduction counseling (including dispensing of condoms), contraceptive counseling, and vaginal ring adherence counseling. Refer to Sections 5.2.2, 5.4, and 5.13, respectively for further details.

d. Instruct subject to remove the vaginal ring and perform pelvic/speculum examination **AT VISITS 2 AND 4 ONLY.** Following the examination, instruct subject to reinsert the ring.

e. Administer acceptability questionnaire **AT VISITS 3 AND 4 ONLY.** ***NOTE:*** *The questionnaire should be administered after the pelvic/speculum examination, if applicable, to assess for ease of removal and insertion.*

f. Schedule next visit.

4.3.2 GROUP B: OBSERVATIONAL SAFETY REGIMEN

a. Obtain and record any adverse events and concomitant medications since the last visit.

b. Obtain locator and menses information.

c. Provide HIV/STI risk reduction counseling (including dispensing of condoms), and contraceptive counseling. Refer to Sections 5.2.2 and 5.4, respectively for further details.

d. Perform pelvic/speculum examination **AT VISITS 2 AND 4 ONLY.**

e. Schedule next visit.

4.4 Visit 5 = 12 Weeks Post-Enrollment Visit & CROSSOVER VISIT (Window ±5 days)

4.4.1 GROUP A: VAGINAL RING TO OBSERVATIONAL SAFETY REGIMEN

a. Obtain and record any adverse events and concomitant medications since the last visit.

b. Obtain locator and menses information.

c. Provide HIV/STI risk-reduction counseling (including dispensing of condoms), pre- and post-test counseling, and contraceptive counseling. Refer to Sections 5.2.2, 5.2.1, and 5.4, respectively for further details.

d. Obtain an oral swab for HIV rapid testing and a urine sample for pregnancy testing.

e. If the HIV rapid test is positive, perform a finger prick (and draw 3 ml venous blood, if necessary) for confirmatory testing, and refer subject to local health facilities for social support or other medical services as clinically indicated. Refer to Section 5.3

for further details. If the pregnancy test is positive, refer subject to the local prenatal clinic for support services. Refer to Section 5.5 for further details.

f. Instruct subject to remove the vaginal ring. Dispose of vaginal ring according to site's biohazard waste disposal policy.
g. Perform pelvic/speculum examination with colposcopy and collect cervico-vaginal swabs for STI testing. Refer to Section 5.9 for further details.

h. Administer acceptability questionnaire. ***NOTE:*** *The questionnaire should be administered after the pelvic/speculum examination to assess for ease of removal and insertion.*

i. Crossover subject into the Observational Safety regimen and schedule next visit. ***NOTE: If the subject has any genital symptomatology or any abnormal findings on the pelvic/speculum examination with colposcopy, DO NOT CROSSOVER. Treat subject as appropriate and reschedule subject to return to Visit 5.1 in 2 weeks.***

4.4.2 GROUP B: OBSERVATIONAL SAFETY TO VAGINAL RING REGIMEN

a. Obtain and record any adverse events and concomitant medications since the last visit.

b. Obtain locator and menses information.

c. Provide HIV/STI risk-reduction counseling (including dispensing of condoms), pre- and post-test counseling, and contraceptive counseling. Refer to Sections 5.2.2, 5.2.1, and 5.4, respectively for further details.

d. Obtain an oral swab for HIV rapid testing and a urine sample for pregnancy testing.

e. If the HIV rapid test is positive, DO NOT enter subject into the Vaginal Ring Regimen. Perform a finger prick (and draw 3 ml venous blood, if necessary) for confirmatory testing, and refer subject to local health facilities for social support or other medical services as clinically indicated. Refer to Section 5.3 for further details. If the pregnancy test is positive, DO NOT enter subject into the Vaginal Ring Regimen, and refer her to the local prenatal clinic for support services. Refer to Section 5.5 for further details.

f. Perform pelvic/speculum examination with colposcopy and collect cervico-vaginal swabs for STI testing. Refer to Section 5.9 for further details.

g. Crossover subject into the Vaginal Ring regimen. ***NOTE: If the subject has any genital symptomatology or any abnormal findings on the pelvic/speculum examination with colposcopy, DO NOT CROSSOVER and do not perform***

procedures h through k listed below. Treat subject as appropriate and reschedule subject to return to Visit 5.1 in 2 weeks.

h. Administer acceptability questionnaire. ***NOTE:*** *The questionnaire must be administered prior to insertion of the vaginal ring.*

i. Dispense one vaginal ring to the subject.

j. Instruct the subject to insert the vaginal ring. Perform brief digital inspection to verify the vaginal ring has been properly placed.

k. Provide vaginal ring adherence counseling. Refer to Section 5.13 for further details.

l. Schedule next visit.

4.5 Visit 5.1 = 2 Weeks Post-Visit 5 & CROSSOVER VISIT (ONLY for those with genital symptomatology or abnormal findings on pelvic/speculum examination with colposcopy at Visit 5)

4.5.1 GROUP A: VAGINAL RING TO OBSERVATIONAL SAFETY REGIMEN

a. Obtain and record any adverse events and concomitant medications since the last visit.

b. Obtain locator and menses information.

c. Provide HIV/STI risk-reduction counseling (including dispensing of condoms) and contraceptive counseling. Refer to Sections 5.2.2 and 5.4, respectively for further details.

d. Perform pelvic/speculum examination with colposcopy.

e. If asymptomatic for genital infections and negative for findings on the pelvic/speculum examination with colposcopy, crossover subject into the Observational Safety regimen and schedule next visit. ***NOTE: If the subject still has any genital symptomatology or any abnormal findings on the pelvic/speculum examination with colposcopy, conclude subject's participation in the trial and follow the subject until her condition is resolved or stabilized.***

4.5.2 GROUP B: OBSERVATIONAL SAFETY TO VAGINAL RING REGIMEN

a. Obtain and record any adverse events and concomitant medications since the last visit.

b. Obtain locator and menses information.

c. Provide HIV/STI risk-reduction counseling (including dispensing of condoms) and contraceptive counseling. Refer to Sections 5.2.2 and 5.4, respectively for further details.

d. Perform pelvic/speculum examination with colposcopy.

e. If asymptomatic for genital infections and negative for findings on the pelvic/speculum examination with colposcopy, crossover subject into the Vaginal Ring regimen. ***NOTE: If the subject still has any genital symptomatology or any abnormal findings on the pelvic/speculum examination with colposcopy, conclude subject's participation in the trial at this point and follow the subject until her condition is resolved or stabilized.***

f. Administer acceptability questionnaire. ***NOTE:*** *The questionnaire must be administered prior to insertion of the vaginal ring.*

g. Dispense one vaginal ring to the subject.

h. Instruct the subject to insert the vaginal ring. Perform brief digital inspection to verify the vaginal ring has been properly placed.

i. Provide vaginal ring adherence counseling. Refer to Section 5.13 for further details.

j. Schedule next visit.

4.6 Visits 6, 7, & 8 = 2, 4, & 8 Weeks Post-Crossover Visit (Window ±5 days)

4.6.1 GROUP A: OBSERVATIONAL SAFETY REGIMEN

a. Obtain and record any adverse events and concomitant medications since the last visit.

b. Obtain locator and menses information.

c. Provide HIV/STI risk reduction counseling (including dispensing of condoms), and contraceptive counseling. Refer to Sections 5.2.2 and 5.4, respectively for further details.

d. Perform pelvic/speculum examination **AT VISITS 6 AND 8 ONLY**.

e. Schedule next visit.

4.6.2 GROUP B: VAGINAL RING REGIMEN

a. Obtain and record any adverse events and concomitant medications since the last visit.

b. Obtain locator and menses information.

c. Provide HIV/STI risk reduction counseling (including dispensing of condoms), contraceptive counseling, and vaginal ring adherence counseling. Refer to Sections 5.2.2, 5.4, and 5.13, respectively for further details.

d. Instruct subject to remove the vaginal ring and perform pelvic/speculum examination **AT VISITS 6 AND 8 ONLY**. Following the examination, instruct subject to reinsert the ring.

e. Administer acceptability questionnaire **AT VISITS 7 AND 8 ONLY.** ***NOTE:*** *The questionnaire should be administered after the pelvic/speculum examination, if applicable, to assess for ease of removal and insertion.*

f. Schedule next visit.

4.7 Visit 9 = 12 Weeks Post-Crossover Visit & LAST STUDY VISIT (Window ±5 days)

4.7.1 GROUP A: OBSERVATIONAL SAFETY REGIMEN

a. Obtain and record any adverse events and concomitant medications since the last visit.

b. Obtain menses information.

c. Provide HIV/STI risk-reduction counseling (including dispensing of condoms), pre- and post-test counseling, and contraceptive counseling. Refer to Sections 5.2.2, 5.2.1, and 5.4, respectively, for further details.

d. Obtain an oral swab for HIV rapid testing and a urine sample for pregnancy testing.

e. If the HIV rapid test is positive, perform a finger prick (and draw 3 ml venous blood, if necessary) for confirmatory testing, and refer subject to local health facilities for social support or other medical services as clinically indicated. Refer to Section 5.3 for further details. If the pregnancy test is positive, refer pregnant women to the local prenatal clinic for support services. Refer to Section 5.5 for further details.

f. Perform pelvic/speculum examination with colposcopy.

g. Collect cervico-vaginal swabs for STI testing. Refer to Section 5.9 for further details.

h. Administer acceptability questionnaire.

i. Conclude subject participation in the study.

4.7.2 GROUP B: VAGINAL RING REGIMEN

a. Obtain and record any adverse events and concomitant medications since the last visit.

b. Obtain menses information.

c. Provide HIV/STI risk-reduction counseling (including dispensing of condoms), pre- and post-test counseling, and contraceptive counseling. Refer to Sections 5.2.2, 5.2.1, and 5.4, respectively, for further details.

d. Obtain an oral swab for HIV rapid testing and a urine sample for pregnancy testing.

e. If the HIV rapid test is positive, perform a finger prick (and draw 3 ml venous blood, if necessary) for confirmatory testing, and refer subject to local health facilities for social support or other medical services as clinically indicated. Refer to Section 5.3 for further details. If the pregnancy test is positive, refer pregnant women to the local prenatal clinic for support services. Refer to Section 5.5 for further details.

f. Instruct subject to remove the vaginal ring. Dispose of vaginal ring according to site's biohazard waste disposal policy.

g. Perform pelvic/speculum examination with colposcopy.

h. Collect cervico-vaginal swabs for STI testing. Refer to Section 5.9 for further details.

i. Administer acceptability questionnaire ***NOTE:*** *The questionnaire should be administered after the pelvic/speculum examination to assess for ease of removal and insertion.*

j. Conclude subject participation in the study.

4.8 Unscheduled Visits

Unscheduled visits may be performed at any time during the study if the subject is experiencing any problems, e.g., vaginal complaints, difficulties with re-inserting the ring in cases of accidental expulsion, or accidental loss of the ring. Subjects will be evaluated and treated appropriately. Referral for treatment at an outside medical facility will be made if necessary.

All unscheduled visits will be documented in the source documents and applicable CRFs.

4.9 Missed & Late Visits

Study staff will make every effort to contact subjects to return to clinic for scheduled visits. If a subject does not return to clinic for a scheduled visit during the study window, e.g., within ±5 days of a scheduled visit, continued attempts to contact the subject should be made and documented in the source documents and applicable CRFs.

If the subject does not return to clinic for a scheduled visit prior to the start of the study window of the next visit, the visit will be considered missed. For example, if a subject does not return for Visit 3 by the time the study window has begun for Visit 4, i.e., within 5 days from Visit 4, Visit 3 will be considered missed. Missed visits will be documented as protocol deviations. ***NOTE:*** *In the event that the subject misses Visit 5 (cross-over visit), i.e. does not return before the start of the window for Visit 6, her participation in the study will be concluded and early study discontinuation procedures will be performed.*

If a subject is outside the window for a scheduled study visit, i.e., >5 days past the study visit date, the clinic should still conduct the regularly scheduled study activities. This will be considered a late visit and all study procedures should still be performed. The subject should then be put back on her original study schedule. ***NOTE:*** *If the subject has any genital symptoms or abnormal findings at a late Visit 5 (cross-over visit), her participation in the study will be concluded and early study discontinuation procedures will be performed.*

4.10 Early Discontinuation Visit

Subjects may be discontinued early from the study prior to completion of the last study visit (12 weeks post-crossover visit) for any of the following reasons:

- Subject withdraws her consent
- Subject fails to follow protocol requirements which are deemed to be serious enough by the investigator to warrant a discontinuation, e.g., in the absence of an adverse event or discomfort, subject refuses to keep vaginal ring inserted for duration of the Vaginal Ring regimen
- Subject is lost to follow-up, i.e., site is unsuccessful in contacting subject or bringing subject back to clinic and subject misses 3 consecutive visits
- At the discretion of the site investigator, Sponsor, IRB/IEC or the government health agency

The date, time, and reason for permanent study discontinuation are to be noted in the source documents and applicable CRFs. All subjects who prematurely discontinue from the study should be encouraged to return to clinic within 2 weeks for a final evaluation, at

which time all study procedures to be performed at the end of the current study regimen the subject is on should be followed. For example, if a subject prematurely discontinues during the Vaginal Ring regimen, all procedures related to the last Vaginal Ring regimen visit (Visit 5 for Group A and Visit 9 for Group B) should be performed. If a subject prematurely discontinues during the Observational Safety regimen, all procedures related to the last Observational Safety regimen visit (Visit 9 for Group A and Visit 5 for Group B) should be performed.

Subjects who miss three (3) consecutive study visits will be considered lost to follow-up and will be permanently discontinued from the study. Contact attempts and final early study termination will be documented in the source documents and applicable CRFs. If a subject already considered lost to follow-up returns to clinic prior to site study completion, the clinic chart (including CRFs) may be re-opened to perform study discontinuation procedures according to the study regimen the subject was following at the time she was lost to follow-up.

Subjects who discontinue early from the study will not be replaced.

4.11 Premature Discontinuation of the Study

The Sponsor has the right to discontinue this study at any time for any reason. If the clinical study is prematurely discontinued, the investigator must promptly inform the subjects and IRB/IECs, and ensure medical follow-up of subjects in consultation with the Sponsor. If the study is prematurely discontinued, all procedures and requirements pertaining to the archiving of documents will be observed. The Sponsor will provide the sites with instructions on the proper disposition of any clinical supplies remaining at the site.

5.1 STUDY PROCEDURE DETAILS

5.2 Informed Consent

The informed consent form will describe the purpose of the study, the procedures to be followed, and the risks and benefits of participation. Two consent forms will be administered: a screening consent form and a study consent form. At the screening visit, potential subjects who agree to screen for the study will sign/date the screening consent form which describes the screening procedures. During the enrollment visit, eligible subjects who agree to participate in the study will sign/date the study consent form which describes all the visits and procedures to be followed during the trial.

The informed consent process should include adequate time for each potential subject to have any study questions answered by the study staff including a qualified physician who is an IPM 011 investigator or co-investigator, and the entire process will be documented in the source records.

At screening, potential subjects who agree to participate in the study will sign and date the screening consent form. The form will be signed and dated by the person administering the consent form and must also be countersigned and dated by a qualified physician who is an investigator or co-investigator in IPM 011 to document his/her involvement in the informed consent process. If a potential subject is functionally illiterate, the consent document(s) and any written study-related materials must be read to her in the language best understood by the potential subject in the presence of an impartial literate observer not affiliated with the study. After the potential subject has orally consented and provided a thumbprint or mark which is witnessed by the impartial observer, this independent observer will sign and date the consent form as a witness.

During the enrollment visit, eligible subjects who agree to participate in the study will sign and date the study consent form which will be signed and countersigned in the same manner as described above at the screening visit.

The signed and dated consent forms must be retained at the study site. A copy of the signed and dated consent forms will be offered to the subject; if the subject is not willing to receive the forms, the second copy will be retained at the site. Likewise during the study, signed and dated consent form updates and any amendments to written study-related materials to be given to subjects will be offered to the subject but retained at the site if the subject is unwilling to receive the forms.

Documentation of the subject's refusal to accept a copy of the informed consent or other study-related materials should be noted in the source documents.

The consent forms and any study-related materials given to the subject will be translated and back-translated and communicated in the local languages by a certified translator. Documentation will be required to verify who performed translation/back-translation of the materials as well as a written statement by the certified translator indicating that the consent form(s) is an accurate translation of the accompanying English version. This is the Principal Investigator's responsibility.

All consent forms must first be reviewed and approved by IPM and then approved by the responsible Institutional Review Board (IRB) and/or local Institutional Ethics Committee (IEC) and National Regulatory Agency prior to administration to the subjects.

If new information becomes available which may be relevant to the subject's willingness to continue study participation, the information will be provided via an IRB and/or IEC-approved revised consent form or addendum to the original consent form in a timely manner and must be signed and dated by the subject in the same manner described above.

5.3 HIV Counseling

5.3.1 HIV Pre- and Post-Test Counseling

At the screening, crossover and last study visit where HIV testing is performed, pre- and post-test counseling will be provided according to the CDC Revised Guidelines for HIV Counseling, Testing, MMWR 2001;50 (No. RR-19):1-58 or on the internet (for reference, see http://www.cdc.gov/mmwr/pdf/rr/rr5019.pdf). Adaptations of these guidelines in accordance with locally accepted standards of practice are allowed. Each study site will document the counseling policies and procedures prior to study implementation for purposes of staff training, quality assurance, and study monitoring.

A comprehensive package of post-test counseling and psychosocial support will be provided to women who test HIV positive during screening or at study termination. Initial counseling services will be provided at the site and women will be referred to additional counseling, support services and, where available, treatment. These services will be identified by IPM prior to study initiation and referral agreements with local providers will be documented in writing

5.3.2 HIV/STI Risk Reduction Counseling

HIV/STI risk reduction guidelines will be developed in conjunction with local voluntary counseling and testing (VCT) guidelines with community input. Counseling will be provided at all study visits including screening. Efforts will be made to ensure the highest quality of risk reduction counseling at the study clinics.

Risk reduction counseling will include recommendation of condom use. Participants will be provided with a regular supply of male non-spermicidal condoms during each study visit.

5.4 HIV Testing & Management

At screening, potential subjects will be tested using the OraSure OraQuick® ADVANCE HIV-1/HIV-2 Rapid Test. If a potential subject tests positive per the OraQuick® test, she is not eligible to enroll in the study. Those who test positive will have a finger prick for confirmatory testing by the Abbott Determine™ HIV-1/2 Rapid Test. If the Determine™ test is positive, the subject is confirmed HIV infected. If the Determine™ test is negative, (discordant results), 3 ml of blood should be drawn and tested using Abbott Murex HIV Ag/Ab Combination EIA. If the Murex HIV Ag/Ab Combination EIA is positive, the subject is confirmed HIV infected. If the Murex HIV Ag/Ab Combination EIA is negative, results are considered indeterminate and the subject will be referred for counseling and repeat testing in 4-6 weeks. (See following tables & Appendix C for testing algorithm). Individuals with confirmed positive results should be counseled and referred to local health facilities for social support or other medical services as clinically indicated.

Laboratory Test	Study Visit	Required Sample/Volume
OraSure OraQuick® ADVANCE HIV-1/HIV-2	Screening, crossover, last study visit	Oral swab

Rapid Test		
Abbott Determine™ HIV-1/2 Rapid Test	As indicated if OraQuick® positive	Finger prick
Abbott Murex HIV Ag/Ab Combination EIA	As indicated if OraQuick® positive and Determine™ negative (discordant results)	3 ml blood

The following table describes the possible HIV testing results and outcomes:

**OraQuick®**	**Determine™**	**Murex EIA**	**Final Outcome**	
–	N/A	N/A	**Not Infected**	**If screening, eligible to enroll**
+	+	N/A	**Infected**	**If screening, do NOT enroll**
+	–	+	**Infected**	**If screening, do NOT enroll**
+	–	–	**Indeterminate** – Provide counseling & have subject return to clinic in 4-6 weeks for repeat testing	**If screening, do NOT enroll**

At the crossover and last study visit, all enrolled subjects will be tested again per the procedures described above. If a Group B subject is confirmed infected at the crossover visit, she will not receive the vaginal ring during the Vaginal Ring regimen but will continue to be followed per the study schedule for safety. Regardless of the results, individuals with indeterminate or positive results should be counseled and referred to local health facilities for social support or other medical services as clinically indicated. Subjects will also be counseled that if they do become HIV positive anytime during the trial, they will immediately discontinue the investigational product (if on the Vaginal Ring regimen) but will continue to be followed per the study schedule for safety.

Subjects in IPM-sponsored trials who become infected with HIV during the course of a trial will be offered appropriate ARV therapy and HIV-related care. The threshold for initiation of ARV treatment will be determined with reference to the host country's treatment guidelines or, if those guidelines are not in place guidelines established by the World Health Organization (WHO). If national or local ARV treatment programs are not in place, IPM will pay for ARV treatment until such resources are available. Women who become pregnant and HIV positive during the trial will be provided with appropriate Prevention of Mother-to-Child Transmission (PMTCT) services.

5.5 Contraceptive Counseling

Subjects will receive contraceptive counseling at all study visits including screening. Counseling will include information about the most reliable birth control options available to the subject which do not utilize a vaginal barrier method (i.e., vaginal hormonal ring, diaphragm, cervical cap or shield) and reminders to continue on a stable oral contraceptive or long-acting progestin regimen or IUD, as applicable. Sites will

either directly provide contraceptives to the subjects for the duration of the trial or make referrals to the appropriate family planning facility to obtain contraceptives.

At the last study visit, subjects will be counseled that they may discontinue contraception for the purposes of study participation if they choose to do so or they may continue on contraceptives if desired.

Subjects will also be counseled that if they do become pregnant during the trial, they will immediately discontinue the investigational product (if on the Vaginal Ring regimen) but will continue to be followed. See *Pregnancy Testing and Management* below in Section 5.5.

5.6 Pregnancy Testing and Management

Urine pregnancy testing will be performed at the screening, enrollment, crossover and last study visit for all subjects. In addition, pregnancy testing will be conducted on-study only in the event the subject misses a menstrual period.

If a potential subject tests positive for pregnancy during screening, she is not eligible to enroll in the study but will receive referrals to prenatal clinics or other appropriate facilities.

If a subject tests positive for pregnancy while on study, she will remove the ring immediately (and permanently) if she is on the Vaginal Ring regimen. If a Group B subject tests positive for pregnancy, she will not receive the vaginal ring during the Vaginal Ring regimen but will continue to be followed per the study schedule for safety.

5.7 Medical History and Physical Examination

At screening and the enrollment visit (pre-enrollment), relevant medical history will be collected including but not limited to history of STIs, gynecological conditions, hospitalizations, surgeries, allergies, any conditions requiring prescription or chronic medication, i.e., >2 weeks in duration, and acute conditions occurring prior to enrollment.

A general physical examination will also be conducted which includes height, weight, vital signs, and examination of skin, respiratory, cardiovascular, central nervous and abdominal systems as well as an assessment of cervical and axillary lymph nodes.

5.8 Pelvic/Speculum Examination

A pelvic/speculum examination will be performed at screening, enrollment, 2 and 8 weeks post-enrollment, crossover, 2 and 8 weeks post-crossover and the last study visit. On-study examinations will be performed to assess safety, i.e., any local vaginal reactions.

The examination will include the following and must be performed by a qualified physician that is an IPM 011 investigator or co-investigator:

- Naked eye examination of vulva
- Speculum examination of vagina and cervix
- Digital and bimanual examination for adnexal or fundal masses or tenderness
- Documentation of genital findings (based on naked eye evaluation) according to the WHO/CONRAD Manual

Each subject will be instructed to contact study staff in advance if menstruation is anticipated to occur on a visit with the pelvic/speculum examination. If the subject is menstruating, all other evaluations will be conducted and the pelvic/speculum examination will be rescheduled at least 2 days after menstruation is completed.

Vaginal Ring regimen only

Although a full pelvic/speculum examination will have been performed earlier, immediately following insertion of the vaginal ring for the first time (enrollment visit for Group A and crossover visit for Group B subjects), a brief digital examination will be performed to verify that the ring has been properly placed by the subject.

At subsequent study visits with a pelvic/speculum examination, the subject will remove the ring prior to the examination. Following the examination (including colposcopy, if applicable), subjects may cleanse the ring with lukewarm water, if desired, and will then reinsert the vaginal ring.

5.9 Colposcopy

Colposcopy will be performed by a qualified physician that is an IPM 011 investigator or co-investigator on all subjects at enrollment, crossover and the last study visit (in conjunction with the pelvic/speculum examination).

Colposcopy will be performed and documented according to the WHO/CONRAD Manual for the Standardization of Colposcopy for the Evaluation of Vaginal Products (Update 2004), Revised Procedure for Colposcopy in the Development of New Vaginal Products.

Both normal and abnormal findings will be documented during each procedure. Additional colposcopies may be performed if abnormalities or lesions are identified or until symptom resolution or at the discretion of the investigator based on signs or symptoms.

5.10 STI Testing

Cervico-vaginal samples will be collected for STI testing at screening and at crossover and the last study visit. All subjects will be evaluated for bacterial vaginosis (BV), trichomonas, gonorrhea (GC), and Chlamydia.

The following tests and procedures will be performed for the associated STIs:

**STI**	**Laboratory Test**
Bacterial Vaginosis	• Wet Mount<br>• Vaginal pH<br>• Whiff Test<br>***NOTE:*** *Tests will done according to Amsel criteria*
Trichomonas	• InPouch culture
Gonorrhea	• *Neisseria gonorrhoeae* Nucleic Acid Test (NG NAT)
Chlamydia	• *Chlamydia trachomatis* Nucleic Acid Test (CT NAT)

Other tests may be performed at the investigator's discretion based on symptomatology and clinical assessment. See separate *Lab Operations Manual* for additional descriptive information regarding specimen collection and processing for all tests.

All original laboratory results should be reviewed by a qualified physician that is an IPM 011 investigator or co-investigator with the review documented on the original laboratory report itself.

STI Treatment

Participants will be treated at the study site or referred to a local health facility for curable STIs per local STI Treatment Guidelines. Those with non-curable STIs, e.g., HSV-2 or HPV, will be referred to a local health facility for treatment per local STI Treatment Guidelines. See *Clinical Management of Genital Diagnoses* (Appendix F) for guidelines to determine whether vaginal ring requires temporary removal and follow-up recommendations.

5.11 Pap Smear

A cervico-vaginal sample will be collected for a Pap smear at screening. Any potential subject with a Pap smear result that requires cryotherapy, biopsy, treatment (other than for infection), or further evaluation including any findings of atypical squamous cells of undetermined significance (ASCUS) is not eligible to enroll in the study but should be referred for medical services as clinically indicated.

5.12 Acceptability Questionnaire

During the Vaginal Ring regimen only, trained staff will administer a confidential vaginal ring acceptability questionnaire as follows: Group A will be administered questionnaires

at enrollment, 4, 8, 12 weeks post-enrollment, and the last study visit; and Group B will be administered questionnaires at enrollment, crossover, 4 and 8 weeks post-crossover and the last study visit.

The questionnaires will be used to assess user perspectives and experiences on the vaginal ring and may include questions about prior use of vaginal rings and other products or objects, traditional vaginal practices, perception of risk for STI/HIV infection, preconceived perceptions of acceptability. Other questions may include ones about the vaginal ring in relation to product characteristics, including ease of use (i.e., insertion and removal), comfort, cleanliness, any instances of accidental expulsion or intentional removal, and use before, during, and after sex as well as perceptions of a subject's partners' reactions. Questions about sexual behavior and condom use may also be asked as well as additional questions about willingness to use this type of ring if it contained a vaginal microbicide approved for protection against HIV infection and preferences for access to such a product.

5.13 Vaginal Ring Insertion & Removal

During the Vaginal Ring regimen only, at the initial visit when the vaginal ring is inserted, i.e., enrollment visit for Group A and crossover visit for Group B, under clinic supervision the subject will be instructed to wash her hands thoroughly, relax, and get into a comfortable position, either standing with one foot on a chair or lying on her back with her knees up. After opening the folds of skin around the vagina, she will gently squeeze the ring into an oval shape and push it upwards and backwards towards the small of the back as far as it will go. She will then be instructed to wash her hands thoroughly again. A brief digital examination will be performed immediately after to verify proper placement of the ring. If upon digital examination the ring is not inserted correctly, the investigator should allow the subject 3 maximum attempts to re-insert the ring properly or provide assistance as required to put the ring in place.

At the last visit of the Vaginal Ring regimen, i.e., crossover for Group A and the last study visit for Group B, under clinic supervision the subject will be instructed to wash her hands thoroughly, relax, and get into a comfortable position, either standing with one foot on a chair or lying on her back with her knees up. She will be instructed to put her finger into her vagina, hook it around the ring, and gently pull downwards and forwards. She will then be instructed to wash her hands thoroughly again. The investigator should allow the subject 3 maximum attempts to remove the ring properly or provide assistance as required to remove the ring. The used vaginal ring should be disposed of according to the site's biohazard waste policy (see Section 6.5).

Subjects will be instructed to refrain from removing the ring over the 12 week Vaginal Ring regimen period except as directed during scheduled study visits. See Section 6.4 for additional instructions in case of accidental ring expulsion or loss.

At the subject's request, trained site staff may provide assistance with insertion or removal of the ring at any time (including at scheduled or unscheduled visits). This should be noted in the source documents and applicable CRFs.

5.14 Vaginal Ring Adherence Counseling

During the Vaginal Ring regimen only, subjects will receive vaginal ring adherence counseling at the time of ring insertion and at every follow-up visit during the Vaginal Ring regimen, i.e., for Group A, this will occur at enrollment and 2, 4, and 8 weeks post-enrollment; and for Group B, this will occur at crossover and 2, 4, and 8 weeks post-crossover visit. Site staff will counsel subjects to refrain from removing the ring (except as directed during clinic visits) and from using concomitant vaginal products or other objects. Site staff will also provide instructions for re-insertion in case of accidental ring expulsion, e.g., during sex or exercise.

5.15 Method of Treatment Assignment

Subjects who meet all of the study inclusion criteria and none of the exclusion criteria at baseline will be randomly assigned in a 1:1 ratio to one of two groups, Group A or Group B.

At each study site, as each new subject enters the study, a sequential SID number will be assigned to that subject. No SID numbers should be skipped or repeated.

Group A will participate in the Vaginal Ring regimen the first 12 weeks and then cross over into the Observational Safety regimen the last 12 weeks of the study. Group B will participate in the Observational Safety regimen the first 12 weeks and then cross over into the Vaginal Ring regimen the last 12 weeks of the study.

5.16 Qualitative Data Component

At each site, approximately 20 women who have completed the Vaginal Ring regimen in IPM 011 at each of the clinical sites may participate in separate focus group discussions within one month after completing the Vaginal Ring regimen depending on the accrual plan and exit schedule. Approximately 20 men who are partners of women who have completed the Vaginal Ring regimen in IPM 011 at each of the clinical sites may participate in individual interviews within one month after completing the Vaginal Ring regimen depending on the accrual plan and exit schedule. The purpose of these discussions and interviews is to assess the acceptability of the vaginal ring and provide additional qualitative information to be used in interpretation of the study's quantitative acceptability instrument.

The enrollment consent form will include information about the possibility of female participation in a focus group and possible invitation to ask the male partner to participate in an individual interview. Participants will be told that their decision to accept or

decline participation in the focus group will not influence their participation in the main study. In addition to the study consent form signed at the screening and enrollment visits, women will sign and date a separate informed consent form prior to participation in the focus group. Men will sign and date an informed consent form prior to participation in the individual interview. See Section 5.1 for more details about informed consent.

5.17 Reimbursement

Subjects will be reimbursed for their time, effort and any travel costs to the study site. Reimbursements will be made after the completion of each study visit. Site specific reimbursement amounts will be documented in the study informed consent approved by the applicable IRB/IEC.

5.18 Study Operations Manual

A separate Study Operations Manual will be supplied to all sites to provide general guidance on the conduct of study procedures.

6.1 INVESTIGATIONAL PRODUCT

6.2 Investigational Product Composition

The vaginal ring selected for use in this study is a silicone elastomer ring developed by Warner Chilcott to deliver a hormone replacement product, estradiol acetate, in their marketed product Femring®. Warner Chilcott received marketing approval from the US Food and Drug Administration in June 2003 for Femring® for the treatment of moderate or severe vasomotor symptoms (e.g., hot flashes) and moderate or severe symptoms of vulvar and vaginal atrophy (e.g., dryness) associated with menopause. Femring® is approved for 3 continuous months of use. The ring to be used in the IPM 011 study is comprised of the same material used to make the silicone vaginal ring for Femring® but will contain no active pharmaceutical ingredient.

The vaginal ring is made of cured silicone elastomer composed of dimethyl polysiloxane silanol, silica (diatomaceous earth), normal propyl orthosilicate, and stannous octoate but will contain no active pharmaceutical ingredient. The ring dimensions are as follows: outer diameter 56 mm, cross-sectional diameter 7.6 mm, core diameter 2 mm.

According to the Pharmacia and Upjohn Company, which received marketing approval from the US Food and Drug Administration in May 1996 for Estring®, an estradiol vaginal ring also made from silicone elastomer and used to treat local symptoms of urogenital atrophy, the biological safety of the silicone elastomer has been studied in various *in vitro* and *in vivo* test models. The results show that the silicone elastomer is non-toxic, non-pyrogenic, non-irritating, and non-sensitizing. Long-term implantation

induced encapsulation equal to or less than the negative control (polyethylene) used in the USP test. No toxic reaction or tumor formation was observed with the silicone elastomer (6).

6.3 Investigational Product Storage

All vaginal rings must be stored between 15°C to 30°C (59°F to 86°F). In the event that the investigational product has been subjected to different storage conditions than specified above, the affected investigational product must not be used (unless IPM or its designee provides written authorization for use). IPM should be notified immediately.

The site investigator (or pharmacist) will maintain an inventory and acknowledge receipt of all shipments of investigational product.

6.4 Investigational Product Administration

During the Vaginal Ring regimen only, subjects will self-insert the ring (enrollment visit for Group A, and the crossover visit for Group B subjects) to be worn for the 12 week duration of the regimen.

6.5 Investigational Product Expulsion or Loss

If the subject accidentally expels the ring, e.g., during sex or exercise, she should be instructed to rinse the ring in lukewarm water and re-insert the vaginal ring. If the ring is expelled in such a manner that the subject is unwilling to re-insert the ring, e.g., during urination or a bowel movement, or if the ring is lost, the subject should be instructed to return to clinic. Based on investigational product availability and evaluation by the investigator, another ring may be dispensed to the subject.

6.6 Investigational Product Accountability

The investigator or designee will be responsible for adequate and accurate accounting, handling, storage and dispensing of investigational product. Investigational product will be stored safely and properly in a secure location with access available only to the investigator and designated study personnel. Investigational product and clinical supplies are to be dispensed only in accordance with the protocol. Accurate records of the investigational product received from IPM, the amount dispensed to the subjects, the amount returned by the subjects, the quantity remaining at the conclusion of the study and any wasted or expired investigational product must be maintained.

At the conclusion of the study, IPM will provide instructions to the sites regarding final disposition of any remaining unused investigational product.

6.7 Concomitant Medications & Products

All prescription and non-prescription medications, including any treatment for STIs and other vaginal infections, will be collected and recorded on the source documents and applicable CRFs.

Concomitant use of vaginal products or other objects including tampons, cotton wool, rags, diaphragms, cervical caps, douches, and drying agents are prohibited for the duration of the study. *NOTE: If any of these products are used, this will be considered a protocol deviation and will be documented on the source document and applicable CRFs.*

7.1 ADVERSE EVENTS

7.2 Definition

An adverse event (AE) is any untoward medical occurrence during the course of a trial in a subject who received investigational product at any dose and that does not necessarily have a causal relationship with the investigational product. An AE can therefore be any unfavorable and unintended sign (including an abnormal laboratory finding), symptom or disease temporally associated with the use of an investigational product, whether or not considered related to the investigational product. This definition includes intercurrent illnesses or injuries and exacerbation of pre-existing conditions.

An *unexpected adverse event* is an adverse reaction, the nature or severity of which is not consistent with the applicable product information (e.g., Investigator's Brochure for an unapproved investigational medicinal product). Final determination of whether an event is considered unexpected will be made by IPM, but the PI should be knowledgeable of the contents of the Investigator's Brochure.

Whenever possible, the laboratory abnormalities should be considered in the context of the primary clinical diagnosis and reported as such (e.g., acute hepatitis with increased bilirubin).

Any condition occurring prior to enrollment (treatment assignment) at Visit 1 will be reported as a pre-existing condition under Medical History. <u>All AEs occurring during the trial will be recorded in the source documents and applicable CRFs</u>.

If possible, a specific disease or syndrome rather than individual associated signs and symptoms should be recorded by the investigator. However, if an observed or reported sign, symptom, or clinically significant laboratory abnormality is not considered a component of a specific disease or syndrome by the investigator, it should be recorded as a separate AE.

All AEs should be monitored until resolution and/or cause is identified or until the PI does not expect any improvement or worsening of condition/symptoms. If an AE remains unresolved at the subject's last study visit, the site investigator will make a clinical

assessment with the Medical Monitor to determine whether continued follow up of the AE is warranted.

7.3 Assessment of Adverse Event Severity

The investigator is responsible for assessing the severity of adverse events occurring on study. All AEs except vulvovaginitis and cervicitis will be graded according to the *Division of AIDS (DAIDS) Table for Grading Severity of Adult Adverse Experiences for the HIV Prevention Trials Network* (See Appendix E). Vulvovaginitis and cervicitis will be graded according to the following table:

Severity Grading Table for Vulvovaginitis and Cervicitis

	Vulvovaginitis	Cervicitis
Grade 1	Vulvar and/or vaginal discomfort (including itching or burning), pelvic exam findings indicative of inflammation, and/or other exam findings* (including findings involving epithelial disruption) that do not require medical therapy and that cause no or minimal interference with usual social and functional activities	Cervical inflammation or other findings on exam (including erythema, mucopurulent discharge, and/or friability) that do not require medical therapy and that cause no or minimal interference with usual social and functional activities
Grade 2	Vulvar and/or vaginal discomfort (including itching or burning), pelvic exam findings indicative of inflammation, and/or other exam findings* (including findings involving epithelial disruption) that require minimal medical therapy (such as a course of topical or oral antibiotics or antifungals) or cause greater than minimal interference with usual social and functional activities	Cervical inflammation or other findings on exam (including erythema, mucopurulent discharge, and/or friability) that require minimal medical therapy (such as a course of oral antibiotics) or that cause greater than minimal interference with usual social and functional activities
Grade 3	Vulvar and/or vaginal discomfort (including itching or burning), pelvic exam findings indicative of inflammation, and/or other exam findings* (including findings involving epithelial disruption) that result in inability to perform usual social and functional activities and/or require significant medical intervention such as a surgical procedure or hospitalization	Cervicitis or other findings on exam (including erythema, mucopurulent discharge, and/or friability) that require significant medical intervention (such as intravenous antibiotics) or that cause inability to perform usual social and functional activities
Grade 4	Life threatening — vulvovaginitis with Perforation	Life threatening

* Findings include erythema, edema, grossly white finding, petechiae, ecchymosis, peeling, ulceration, abrasion, laceration.

For AEs not listed on the DAIDS table, the following criteria will be used to estimate the grade of severity:

- **Mild**

  Transient or mild discomfort (<48 hours); no medical intervention/therapy required

- **Moderate**

  Mild to moderate limitation in activity – some assistance may be needed; no or minimal medical intervention/therapy required

- **Severe**

  Marked limitation in activity, some assistance usually required; medical intervention/therapy required; hospitalizations possible

- **Life-threatening**

  Extreme limitation in activity, significant assistance required; significant medical intervention/therapy required; hospitalization or hospice care probable

7.4 Relationship to Investigational Product

The investigator is responsible for determining the relationship of all AEs occurring on study and will assess AEs based on the following criteria:

- **Not Related**

  The AE is clearly explained by another cause (concurrent disease, concomitant medication, environmental or toxic factors, etc.).

- **Probably Not Related**

  The AE is more likely explained by another cause (concurrent disease, concomitant medication, environmental or toxic factors, etc.).

- **Possibly Related**

  The AE is equally likely explained by another cause but the possibility of the investigational product relationship cannot be ruled out, e.g., the administration of the investigational product and AE are considered reasonably related in time.

- **Probably Related**

  The AE is more likely explained by the investigational product, e.g., the administration of the investigational product and AE are considered reasonably related in time and the AE is less likely explained by another cause.

- **Definitely Related**

The AE is clearly related and most likely explained by the administration of the investigational product.

7.5 Serious Adverse Events

7.4.1 Serious Adverse Event Definition

A Serious Adverse Event (SAE) is defined as any untoward medical occurrence that at any dose meets any of the following criteria:

- Results in death
- Is life-threatening

  This criterion applies if the subject is at immediate risk of death from the event as it occurred, in the opinion of the investigator; it does not refer to an event which hypothetically might have caused death if it were more severe.
- Requires inpatient hospitalization or prolongation of existing hospitalization

  This criterion applies if the event requires at least a 24-hour inpatient hospitalization or, if in the opinion of the investigator, prolongs an existing hospitalization. A hospitalization (including hospitalization for an elective procedure or routinely scheduled treatment) for a pre-existing condition which has not worsened does not constitute a SAE.
- Results in persistent or significant disability/incapacity

  This criterion applies if the event causes a substantial disruption of a person’s ability to conduct normal life functions.
- Is a congenital anomaly/birth defect

  This criterion applies if a subject gives birth to a child with a congenital anomaly or birth defect.
- Is an important and significant medical event that may not be immediately life threatening or result in death or hospitalization but, based upon appropriate medical judgment, may jeopardize the subject or require intervention to prevent one of the other outcomes listed above. e.g., bronchospasm requiring intensive treatment in an emergency room or at home.

NOTE: An SAE need not be severe in nature to meet any of the above criteria.

All SAEs that occur from the time the subject is enrolled (receives treatment assignment) through the duration of the study, whether considered to be associated with investigational product or not, must be reported to IPM within 24 hours of the site becoming aware of the event. All SAEs should be reported using the designated SAE Report Form.

The SAE Report Form should be completed with all available information at the time of reporting. The investigator is required to write a detailed written report and complete SAE follow-up in a timely manner until the SAE returns to baseline, subject returns to normal health or until the investigator does not expect further improvement or worsening of the event. Medical records may be requested by IPM to assist in assessing relatedness and severity of the SAE, and for possible submission to Regulatory or Health authorities. To maintain confidentiality, the subject's name must be blacked out and replaced with the Participant Identification Number and initials on any medical records submitted.

More details on SAE reporting requirements are described in separate SAE Reporting Guidelines.

7.4.2 Serious Adverse Event Contact Information

Serious Adverse Events will be faxed to IPM within 24 hours of becoming aware of the event.

If the SAE is related, and life-threatening or fatal, immediately telephone the Medical Monitor.

CONTACT INFORMATION

Medical Monitor	Dr. Katherine Young, or designee
Office Phone:	+27 21 860 2300
Mobile Number:	+27 79 881 9819
Office Fax:	+27 21 860 2308
Office Email:	kyoung@ipm-microbicides.org.za

IPM will process all safety events. The Medical Monitor will review all SAEs and generate the necessary queries.

7.4.3 Sponsor Notification of Unexpected SAEs to Regulatory Agencies

Any unexpected serious adverse event which is deemed to be "Definitely Related", "Probably Related", or "Possibly Related" to the investigational product will be considered "associated with the use of the investigational product" and thus IPM will notify appropriate regulatory authorities of the event in an expedited manner.

Any unexpected serious adverse event deemed to be "Probably Not Related" or "Not Related" will not be reported to regulatory authorities in an expedited manner.

7.4.4 Site Notification of SAEs to Local Ethics Committee or Local Health or Regulatory Authorities

The investigator will report all SAEs to the local Ethics Committee (EC) and/or health or regulatory authorities in accordance with standard operating procedures and policies of the EC and/or health or regulatory authorities.

8.1 DATA MANAGEMENT

8.2 Data Handling at Study Sites

All study data will first be collected on designated source documents and then recorded on Case Report Forms (CRFs) unless otherwise specified by IPM. Site staff responsible for completing the CRFs will receive proper training prior to trial start and will follow standardized procedures. Data must be legibly entered onto the CRFs. Data corrections will be made in accordance with standard procedures provided by IPM or its designee.

The investigator will maintain, and store in a secure manner, complete, accurate and current study records throughout the study. Standard GCP practices will be followed to ensure accurate, reliable and consistent data collection.

8.3 Source Data Verification

All study data must be verifiable to the source documentation (which includes original recordings, laboratory requisitions and reports, medical records, etc.). Source documentation will be available to the Sponsor or representative(s) for review to ensure that the collected data is consistent with the CRFs and has been completely and accurately reported as required by the study protocol.

9.1 STATISTICAL METHODS

9.2 General Design

IPM 011 is an open-label crossover study conducted at 5 sites in Kenya, South Africa and Tanzania among 230 healthy sexually active women to assess the safety and acceptability of a silicone elastomer vaginal ring (containing no drug) intended as a microbicide delivery method for the prevention of HIV infection. Upon enrollment at each site, subjects will be assigned in a 1:1 ratio to one of two study groups, Group A and Group B. Both groups will participate in two regimens: Vaginal Ring and Observational Safety (with no vaginal ring).

Group A will participate in the Vaginal Ring regimen for the first 12 weeks of the study, self-insert a vaginal ring at enrollment (Visit 1) to be worn for 12 continuous weeks, and be followed 2, 4, 8, and 12 weeks post-enrollment ring insertion (Visits 2-5) to monitor safety and acceptability. After the first 12 week period is completed, Group A will

remove the vaginal ring, and if asymptomatic for genital infections and negative for findings on the pelvic/speculum examination with coloposcopy, will cross over into the Observational Safety regimen at Visit 5 for the last 12 weeks of the study, and return to the clinic 2, 4, 8 and 12 weeks post-crossover visit (Visits 6-9) for follow-up observational safety only. If the subject has any genital symptomatology or findings on the pelvic/speculum examination with colposcopy after the first 12 week period is completed, she will not cross over into the Observational Safety regimen but she will be treated as appropriate and will return to the clinic within 2 weeks (Visit 5.1). At that time, if the subject is asymptomatic for genital infections and negative for findings on the pelvic/speculum examination with colposcopy, she will cross over into the Observational Safety regimen.

Group B will participate in the Observational Safety regimen for the first 12 weeks of the study and after enrollment (Visit 1) will be followed 2, 4, 8, and 12 weeks post-enrollment (Visits 2-5) for observational safety only. After the first 12 week period is completed, if asymptomatic for genital infections and negative for findings on the pelvic/speculum examination with coloposcopy, Group B will cross over into the Vaginal Ring regimen at Visit 5 for the last 12 weeks of the study, self-insert a vaginal ring to be worn for 12 continuous weeks, and be followed 2, 4, 8 and 12 weeks post-crossover ring insertion (Visits 6-9) to monitor safety and acceptability. If the subject has any genital symptomatology or findings on the pelvic/speculum examination with colposcopy after the first 12 week period is completed, she will not cross over into the Vaginal Ring regimen but she will be treated as appropriate and will return to the clinic within 2 weeks (Visit 5.1). At that time, if the subject is asymptomatic for genital infections and negative for findings on the pelvic/speculum examination with colposcopy, she will cross over into the Vaginal Ring regimen.

During the 24 week study period, all subjects (Groups A and B) will undergo a general physical examination at screening, and a pelvic/speculum examination at screening, enrollment, 2 and 8 weeks post-enrollment, crossover, 2 and 8 weeks post-crossover and the last study visit. At enrollment, the crossover visit, and the last study visit, all subjects will also undergo a colposcopy examination. STI testing will be performed at screening, the crossover visit and the last study visit. A Pap smear will be done at screening. HIV/STI risk reduction counseling including condom dispensing and contraceptive counseling will be provided at all study visits including screening. Vaginal ring adherence counseling will be provided at the time of ring insertion and at every follow-up visit during the Vaginal Ring regimen only, i.e., for Group A, this will occur at enrollment and 2, 4, and 8 weeks post-enrollment ring insertion; and for Group B, this will occur at the crossover visit and 2, 4, and 8 weeks post-crossover ring insertion visit. HIV testing with pre- and post-test counseling will be conducted at screening, the crossover visit and the last study visit. In addition to screening, enrollment, the crossover visit and the last study visit, pregnancy testing will only be conducted in the event the subject misses a menstrual period during the study. An acceptability questionnaire will be administered during the Vaginal Ring regimen, i.e., for Group A, this will occur at enrollment and 4, 8, 12 weeks post-enrollment ring insertion and the last study visit; and

for Group B, this will occur at enrollment, the crossover visit and 4, 8 and 12 weeks post-crossover ring insertion visit. Adverse events, including vaginal complaints, and concomitant medications will be assessed at every visit

Each subject will be followed on-study for a total of 24 weeks (i.e., two 12-week regimens) or 26 weeks if 2 additional weeks are required in the event the crossover visit is delayed due to genital symptomatology or findings on pelvic/speculum examination with colposcopy).

Approximately 20 women at each site who have completed the Vaginal Ring regimen in IPM 011 at each of the clinical sites may participate in separate focus group discussions within one month after completing the Vaginal Ring regimen depending on the accrual plan and exit schedule. Approximately 20 men who are partners of women who have completed the Vaginal Ring regimen in IPM 011 at each of the clinical sites may participate in individual interviews within one month after the women have completed the Vaginal Ring regimen depending on the accrual plan and exit schedule. The purpose of these discussions and interviews is to provide additional information on acceptability of the vaginal ring, in order to strengthen interpretation of findings from the acceptability questionnaire administered during the study

9.3 Sample Size

230 sexually active women (between 20 and 60 per site) will be enrolled in this study with competitive enrollment.

9.4 Analyses

The primary focus of the analysis will be safety and acceptability measures. The primary analysis will be conducted on the intent-to-treat population, with some analyses conducted on the per-protocol population or subgroups of particular interest, as appropriate.

Simple descriptive statistics will be generated for each variable. For continuous variables, they include, but are not limited to overall mean (when appropriate), mean by study group, mean by site, variance of the means, range, and number of missing data points. For categorical variables they include overall frequencies (when appropriate), frequencies by study group, and frequency listing by site. This section discusses the planned analyses beyond the listings and simple descriptive analyses.

9.4.1 Analysis of Safety Measures

Safety of the ring will be assessed using clinical measures collected through pelvic/speculum examinations performed during specified study visits, in combination

with colposcopy observations at the crossover and last study visit (see Appendix A and B for scheduled clinical procedures). Adverse events reported will also be used to assess the safety of the ring. The primary measure will be the proportion of women who have abnormal observations during the pelvic/speculum examinations and the proportion of women who experience adverse events. Comparisons will be made with two-sided t-test between women who are on the Vaginal Ring Regimen and women who are on the Observational Safety Regimen. Each woman will also be compared to herself using pelvic/speculum observation data obtained during the two regimens. It is expected that women who have existing conditions will be treated and evaluated before being enrolled into the study and at time of crossover. As a result, a baseline adjustment is not considered as necessary. However, data will be evaluated and analysis strategies will be adjusted based on the evaluation.

Safety measures will be correlated with other clinical data, such as STD and HIV testing, since genital abnormalities can be caused by medical conditions rather than the product itself. In addition, when analyzing safety measures, sexual practice, regimen adherence (from Adherence Baseline and Follow-Up Questionnaires), product use experience, and vaginal practice (from Acceptability Questionnaire) will be used as covariates and be controlled for.

9.4.2 Analysis of Acceptability Measures

Instead of being protocol specific and focused on pre-established hypotheses/endpoints, the analysis will be exploratory in understanding factors that influence the product's acceptability, as guided by the conceptual framework and research questions. The acceptability of the ring will be assessed using the variables selected from the following group of variables of the Acceptability Questionnaire: Willingness to Use, Product Use Experience, and Product Characteristics. The acceptability questionnaire will be administered to enrolled subjects at enrollment (to assess pre-use vaginal product behavior) and 4, 8, and 12 weeks post-enrollment and the last study visit for Group A; and at enrollment, the crossover visit and 4, 8 and 12 weeks post-crossover visit for Group B. Group differences between women who are under the Vaginal Ring Regimen and who are not will be tested using two-sided t-tests. This comparison may need to be limited to data collected before the changeover only, since data from the group of women who change from the Vaginal Ring Regimen to the Observational Safety Regimen could be contaminated by their experience prior to the changeover (the so called carry-over effect).

The study's primary measure of acceptability will be defined as the proportion of women indicating that they would use the study product if it is found to prevent HIV. Data collected from the following questions in the Exit Section of the Acceptability Follow-Up Questionnaire will be used:

- Do you like having the ring in place every day?
- If rings like this are someday available to prevent HIV, who do you think would like to use them?

- If rings like this are someday available for women to prevent HIV, would they be acceptable to men?
- If it is recommended that you use both condoms and the ring for sex, would you use both?
- In general, do you think people will approve of the ring?
- In general, do you think community leaders will approve of the ring?

Women's perceived risk of HIV, sexual practice and vaginal practice will be used as covariates in analyzing the "willingness to use" variables. In addition, adherence variables will also be used to adjust for possible confounding factors.

Changes in attitude (based on "willingness to use" variables) within a subject will be investigated by comparing means of the scores between the two regimens and by growth curve models. For Group B, the group that starts with the Observational Safety Regimen first, the mean attitude scores from the pre- and post-treatment periods will be compared. This comparison can be done at group level (i.e., group level means for pre- and post-treatment will be compared). Each woman in the group can also form a pair of observations to allow paired t-tests. If the change in attitude scores during the Vaginal Ring Regimen is of interest, then attitude scores can be modeled for both groups as a function of time, as well as other covariates, through a growth curve model.

Data collected in the Product Use Experience and Product Characteristics sections of the Acceptability Questionnaire will be important for understanding both the product's acceptability and study regimen adherence. Proportions of women who respond "Yes" to each of the questions will be calculated for each study group, study group by site, as well as two study groups combined. It is not expected that the random assignment will influence women's experience with the product nor the product characteristics.

Since the study is a 2 X 2 cross-over design, potentially there is a carry-over effect. Therefore the difference of the two arms during the first 12 weeks and the second 12 weeks is also of interest and can be investigated through formal statistical testing.

Another aspect of the acceptability analysis is to investigate factors that influence the subjects' attitudes and predict subject responses to the study product for the planned main study. For such a purpose, we will conduct conditional regression or logistic regression, using subjects as strata. The acceptance measures will be regressed over possible predictors and statistical significant levels of the coefficients will be tested.

9.4.3 Adherence Analysis

The main purpose of analyzing adherence data, in addition to using them in acceptability and safety data analysis, is to understand the factors that influence adherence behavior. To this end, the proportion of women who did not keep the ring inserted during the last 7 days will be computed for each of the visits for both study groups. Weekly and monthly rates will be presented by (1) study group and (2) by study group and site. The

cumulative number of days of ring not being inserted during the 12 week Vaginal Ring Regimen period will also be calculated by (1) study arm and (2) study arm by site. Multivariate regression models will be used to identify significant predictors on adherence. The proportion of times the ring is inserted can be modeled through a logistic regression model with covariates of sexual practice, vaginal practice, and product characteristics.

9.4.4 Condom Use and Sexual Behavior

Analyzing condom use and sexual behavior data will allow better understanding of who will most benefit from using the product and will inform the study design for the next stage of product development. The proportion of women with no coital activity in the last 7 days will be computed. These weekly rates will be presented in tables by (1) study arm, (2) study arm by regimen, and (3) study arm by regimen by site. Appropriate statistical tests will be used to compare study arms within study regimen.

Also, for each woman with at least one sexual vaginal act during the 12 weeks of ring use, the four following proportions will be computed:

- Number of acts in which condom only was used divided by the total number of coital acts
- Number of acts in which ring only was used divided by the total number of coital acts
- Number of acts in which ring and condoms were used divided by the total number of coital acts
- Number of acts in which ring and condoms were not used divided by the total number of coital acts

Appropriate summary measures of these four proportions will be presented in tables and graphs by (1) study group and (2) study group by site. Appropriate statistical tests will be used to compare study arms within frequency of use.

9.4.5 Handling of Missing Data and Dropouts

It is expected that a certain amount of data will be missing, mostly due to missed visits. In general, missing data imputation is not recommended except in situations where variables are required for other analysis (such as a regression modeling). Those cases will be considered on a case-by-case basis. However, missing data patterns may provide insight into answering research questions on acceptability and regimen adherence when analyzed with data collected before the missed visits and after the subject returns. Exit interviews will be attempted for each dropout subject. Data collected from the exit interview will be analyzed as a part of the acceptability analysis. However, if there are a large number of subjects where exit interviews are not available, a survival analysis on the dropout subjects may yield useful information. In such an analysis, a time-to-event model (where event is defined as dropping out) using the Cox model will allow incorporation of covariates collected up to the point of dropping out. Significant factors that contribute to the dropouts may be identified

10.1 INVESTIGATOR REQUIREMENTS

10.2 Study Initiation

The study cannot be initiated at the site until the site has been fully qualified with the Sponsor. Following Sponsor approval, IPM will notify the site in writing via letter correspondence to begin study operations according to the protocol and all other related study materials.

Prior to implementation, the following documents must be on file with IPM or its representative:

- Protocol signature page signed and dated by the Principal Investigator
- Investigator Brochure signature page signed and dated by the Principal Investigator
- Pre-study visit report
- Study Site Counseling Checklist signed & dated by IPM or its representative
- Statement of Investigator signed and dated by the Principal Investigator
- All co-investigators must be listed on the Statement of Investigator. Investigators must also complete all regulatory documentation as required by local and national regulations
- Current signed and dated curricula vitae of the Principal Investigator and all co-investigators which includes medical licensure and/or medical qualifications and cites the association that the Principal Investigator and co-investigators have with the medical-site institution
- Current signed and dated curricula vitae of the Laboratory Director or designee
- Signed and dated Financial Disclosure Forms for Principal Investigator and all co-investigators listed on the Statement of Investigator
- Institutional Review Board and/or Institutional Ethics Committee (IRB/IEC) membership list
- Written documentation of IRB/IEC and/or other national ethics committee (if applicable) approval of protocol and informed consent document (both identified by study protocol number or title and date of approval)

- Copy of the IRB/IEC-approved informed consent document

- Written documentation of IRB/IEC review and approval of any advertising materials to be used for study recruitment as well as subject information

- Current laboratory certification of the laboratory performing the analysis (if available), as well as current normal laboratory ranges for all laboratory tests

- Signed Clinical Study Agreement

- Certified translations and back-translations of approved informed consent document, and pertinent correspondence (when applicable)

- Other country-specific required documents including regulatory authority approval or acknowledgement of receipt of notification

All regulatory and ethics committee(s) submissions need to be reviewed and approved by IPM prior to submission to the applicable agencies.

10.3 Institutional Review Board or Institutional Ethics Committee Approval

This protocol, the informed consent document, and relevant supporting information must be submitted to the IRBs/IECs for review and must be approved before the study is initiated.

The Principal Investigator is responsible for communicating with IRBs and/or IECs regarding the progress of the study and changes made to the protocol as deemed appropriate, but in any case at least once a year. The Principal Investigator must also keep the IRBs/IECs informed of any significant adverse events and SAEs.

10.4 Study Monitoring and Audits

Study monitors will regularly visit participating study sites to review all study documents including but not limited to individual subject records, consent forms, source documents, CRFs, supporting data, laboratory specimen records and medical records (physicians' progress notes, nurses' notes, individuals' hospital charts) to ensure protection of study subjects, compliance with the protocol, and accuracy and completeness of records. The site monitors also will inspect the site's regulatory files to ensure that regulatory requirements are being followed; the site's pharmacies to review product storage, management, and drug accountability; and the site's laboratory and other clinical supplies to ensure proper storage and continued viability of supplies. All applicable study documents should be readily available for review during the visits. The site monitors will also check that clinical study procedures are observed and will discuss any problems with investigator or designee as applicable.

During or after the clinical study, the governmental regulatory authorities, local IRB/IEC and/or representatives of the Sponsor may request access to all study documents for on-site audit or inspection.

10.5 Case Report Forms

Case Report Forms (CRFs) will be supplied by IPM or its designee and should be handled in accordance with instructions from IPM.

All CRFs should be filled out completely by the designated study staff. Upon study completion, the CRF is reviewed, signed, and dated by the investigator or co-investigators listed on the Statement of Investigator.

All CRFs should be completed in a neat, legible manner to ensure accurate interpretation of data. Black ink is required to ensure clarity. When making changes or corrections, the original entry should be crossed out with a single line, and the change initialed and dated. Erasures, overwriting, and correction fluid are NOT allowed on the CRFs.

10.6 Disclosure of Data

Subject medical information is confidential and disclosure to third parties other than those described in Section 10.3 is strictly prohibited. All study data will be stored securely at the study site. All subject information including laboratory specimens, reports, forms, lists, logbooks, appointment books and administrative forms will be stored in locked file cabinets in areas with access limited to study staff.

Subjects' study information will not be released without written permission of the subject, except as necessary for monitoring by the Sponsor, Sponsor's designated monitors, or regulatory authorities.

10.7 Record Retention

The investigator will retain in a secure manner, complete, accurate and current study records for a minimum of two years after marketing approval or termination of product development. Study records include administrative documentation, including site registration documents and all reports and correspondence relating to the study, as well as documentation related to each subject screened and/or enrolled in the study, including informed consent forms, CRFs, notations of all contacts with the subject, and all other source documents. All records must be retained on-site throughout the study's period of performance. The Sponsor will provide the study site with written instructions for long-term record storage at the completion of the study.

No records should be destroyed without prior written permission from IPM.

11.1 ETHICAL CONSIDERATIONS

11.2 Ethical Review

This protocol, site-specific informed consent forms, subject education, outreach, recruitment materials and any other requested documents or subsequent modifications will be reviewed and approved by the ethical review bodies responsible for oversight of research conducted at the study site.

Subsequent to initial review and approval, the local Institutional Review Board (IRB) and/or Institutional Ethics Committee (IEC) will be notified about study completion within three months following study termination or completion.

This study will be conducted in accordance with the ethical principles of:

- World Medical Association Declaration of Helsinki http://www.wma.net/e/policy/b3.htm (11)
- International Conference on Harmonisation (ICH) Good Clinical Practice (GCP) guidelines (See Guidance for Industry: E6 Good Clinical Practice: Consolidated Guidance http://www.fda.gov/cder/guidance/959fnl.pdf) (12)
- Applicable regulatory requirement(s).

11.3 Social Harms

Social harms including discrimination, loss of job opportunities, difficulties with insurance or military service, or disruption of family or personal relationships may result due to participation in this trial becoming known to others. In addition, investigational product use could potentially not be acceptable to the participant's sex partner and result in difficulties with her spouse or sex partner. If a participant is or becomes HIV-infected, she may also experience social harms.

During each HIV counseling session, participants will be asked questions to address the occurrence of social harms. Participants who experience social harms will be counseled accordingly and provided with assistance to mitigate the circumstances if possible. This will be recorded in the source documents and applicable CRFs.

12.0 PUBLICATION

Any presentation, abstract, or manuscript shall be reviewed and approved by the Sponsor prior to submission. Publication of the results of this trial will be governed by the Sponsor's publication policies. Authorship criteria will be based on contributions to the design, work, and analysis of the study.

13.1 REFERENCES

1. Joint United Nations Programme on HIV/AIDS (UNAIDS). 2006 Report on the Global AIDS Epidemic, Executive Summary; May 2006
2. Warner Chilcott Femring® Full U.S. Prescribing Information (http://www.warnerchilcott.com/dtcfemring/femring_hcp.php)
3. Warner Chilcott Femring® Patient Information Materials: Patient Acceptability. (http://www.warnerchilcott.com/pdf/FemrPatAccept.pdf)
4. World Medical Association Declaration of Helsinki. Ethical Principles for Medical Research Involving Human Subjects; WMA General Assembly, Tokyo 2004 http://www.wma.net/e/policy/b3.htm
5. Guidance for Industry: E6 Good Clinical Practice: Consolidated Guidance (http://www.fda.gov/cder/guidance/959fnl.pdf).
6. Pharmacia & Upjohn Estring® Physician Leaflet (http://www.pfizer.com/pfizer/download/uspi_estring.pdf)

APPENDIX A: SCHEDULE OF CLINICAL PROCEDURES GROUP A

		VAGINAL RING				CROSSOVER		OBSERVATIONAL SAFETY			
**Study Visit**	**Screening**	**1**[a]	**2**[b]	**3**	**4**	**5**	**5.1**[c]	**6**	**7**	**8**	**9**
		Enrollment	2 wk post-enroll	4 wk post-enroll	8 wk post-enroll	Crossover	ONLY if needed	2 wk post-crossover	4 wk post-crossover	8 wk post-crossover	Last Study Visit
Informed Consent	X	X									
Demographics & Medical History	X	X[d]									
Inclusion/Exclusion Criteria	X	X[d]									
Locator Information	X	X[d]	X	X	X	X	X	X	X	X	
Menses Information	X	X[d]	X	X	X	X	X	X	X	X	X
General Physical Examination	X										
Pelvic/Speculum Examination	X	X[d]	X		X	X	X	X		X	X
Colposcopy		X[d]				X	X				X
HIV/STI Risk Reduction Counseling (with Condoms)	X	X[d]	X	X	X	X	X	X	X	X	X
HIV Pre- & Post-Test Counseling	X					X					X
Vaginal Ring Adherence Counseling		X	X	X	X						
Contraceptive Counseling	X	X[d]	X	X	X	X	X	X	X	X	X
HIV-1 Rapid Testing[e]	X					X					X
Urine Pregnancy Testing[f]	X	X[d]				X					X
Cervico-vaginal Sample Collection for STI Testing	X					X					X
Cervico-vaginal Sample Collection for Pap smear	X										
Ring Acceptability Questionnaire		X		X	X	X					X
Vaginal Ring Insertion[g]		X									
Adverse Event			X	X	X	X	X	X	X	X	X
Concomitant Medication Evaluation	X	X[d]	X	X	X	X	X	X	X	X	X
Vaginal Ring Removal						X					
Study Completion											X

[a] Study Week 0 (enrollment) must occur within 30 days of screening.
[b] The study window for all visits except for screening, enrollment and Visit 5.1 is ±5 days.
[c] Visit 5.1 will be done 2 weeks post-visit 5 only for those with genital symptomatology or abnormal findings on pelvic/speculum examination with colposcopy at Visit 5.
[d] The specified procedures occur prior to enrollment. If all the inclusion criteria and none of the exclusion criteria continue to be met, subject is eligible to enroll immediately.
[e] Subjects who test HIV-1Rapid Test positive will have a finger prick (and 3 ml blood drawn, if necessary) for confirmatory testing.
[f] In addition to the time points noted, urine pregnancy testing will be conducted on-study only in the event the subject misses a menstrual period.
[g] Once the vaginal ring is inserted by the subject, the ring should not be removed during the 12 week Vaginal Ring regimen except as directed at study visits.

APPENDIX B: SCHEDULE OF CLINICAL PROCEDURES GROUP B

		OBSERVATIONAL SAFETY				CROSSOVER		VAGINAL RING			
**Study Visit**	**Screening**	**1**[a]	**2**[b]	**3**	**4**	**5**	**5.1**[c]	**6**	**7**	**8**	**9**
		Enrollment	2 wk post-enroll	4 wk post-enroll	8 wk post-enroll	Crossover	ONLY if needed	2 wk post-crossover	4 wk post-crossover	8 wk post-crossover	Last Study Visit
Informed Consent	X	X									
Demographics & Medical History	X	X[d]									
Inclusion/Exclusion Criteria	X	X[d]									
Locator Information	X	X[d]	X	X	X	X	X	X	X	X	
Menses Information	X	X[d]	X	X	X	X	X	X	X	X	X
General Physical Examination	X										
Pelvic/Speculum Examination	X	X[d]	X		X	X	X	X		X	X
Colposcopy		X[d]				X	X				X
HIV/STI Risk Reduction Counseling (with Condoms)	X	X[d]	X	X	X	X	X	X	X	X	X
HIV Pre- & Post-Test Counseling	X					X					X
Vaginal Ring Adherence Counseling						X[e]	X	X	X	X	
Contraceptive Counseling	X	X[d]	X	X	X	X	X	X	X	X	X
HIV-1 Rapid Testing[f]	X					X					X
Urine Pregnancy Testing[g]	X	X[d]				X					X
Cervico-vaginal Sample Collection for STI Testing	X					X					X
Cervico-vaginal Sample Collection for Pap smear	X										
Ring Acceptability Questionnaire		X				X[e]	X		X	X	X
Vaginal Ring Insertion[h]						X[e]	X				
Adverse Event			X	X	X	X	X	X	X	X	X
Concomitant Medication Evaluation	X	X[d]	X	X	X	X	X	X	X	X	X
Vaginal Ring Removal											X
Study Completion											X

[a] Study Week 0 (enrollment) must occur within 30 days of screening.
[b] The study window for all visits except for screening, enrollment and Visit 5.1 is $\pm$5 days.
[c] Visit 5.1 will be done 2 weeks post-visit 5 only for those with genital symptomatology or abnormal findings on pelvic/speculum examination with colposcopy at Visit 5.
[d] The specified procedures occur prior to enrollment. If all the inclusion criteria and none of the exclusion criteria continue to be met, subject is eligible to enroll immediately.
[e] If a Visit 5.1 is required, these procedures will be delayed until Visit 5.1 and the crossover visit is made.
[f] Subjects who test HIV-1Rapid Test positive will have a finger prick (and 3 ml blood drawn, if necessary) for confirmatory testing.
[g] In addition to the time points noted, urine pregnancy testing will be conducted on-study only in the event the subject misses a menstrual period.
[h] Once the vaginal ring is inserted by the subject, the ring should not be removed during the 12 week Vaginal Ring regimen except as directed at study visits.

APPENDIX C: HIV TESTING ALGORITHM

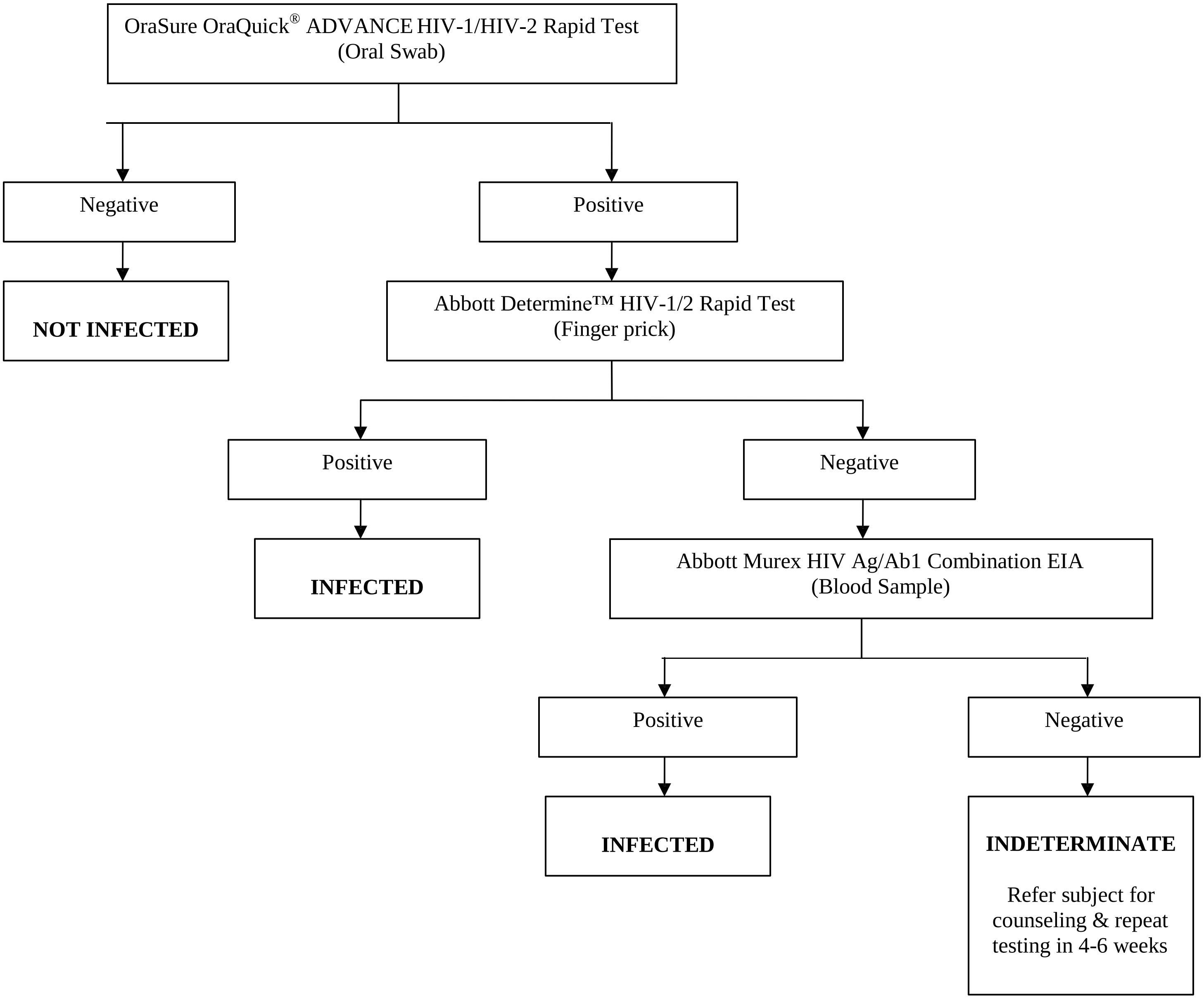

APPENDIX D: VAGINAL RING INSERTION & REMOVAL INSTRUCTIONS

HOW DO I USE THE VAGINAL RING?

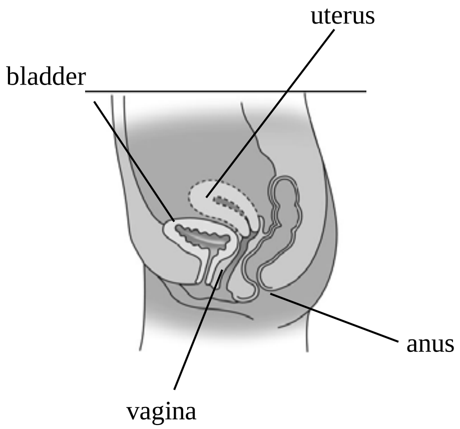

TO INSERT THE RING INTO YOUR VAGINA:

1. Wash and dry your hands.
2. Remove the ring from its package.
3. Choose the position that is most comfortable for you. For example, lying down or standing with one leg up. (See **Diagrams 1a and 1b**, respectively).

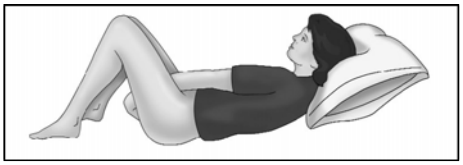

DIAGRAM 1a

OR

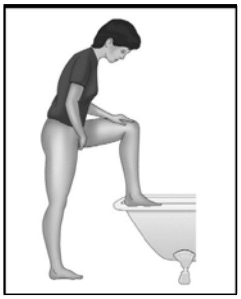

DIAGRAM 1b

4. Use your thumb and index finger (pointer finger) to press the sides of the ring together. You may find it easier to insert the ring if you twist it into a figure-of-eight shape. (See **Diagram 2**)

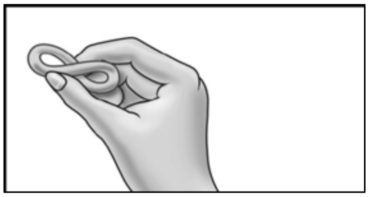

DIAGRAM 2

5. Use your other hand and hold open the folds of skin around your vagina. (See **Diagram 3**)

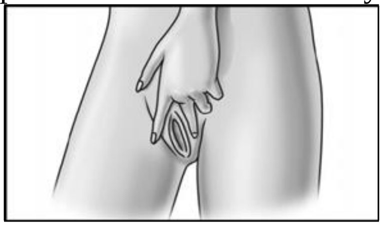

DIAGRAM 3

6. Place the tip of the ring in the vaginal opening and then use your index finger to push the folded ring gently into your vagina. Push it up towards your lower back as far as you can. (See **Diagram 4**)

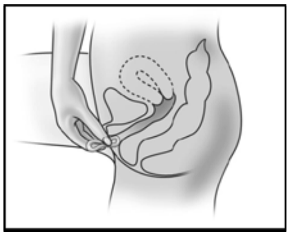

DIAGRAM 4

If the ring feels uncomfortable, you probably did not push it into your vagina far enough. Use your index finger to push the ring as far as you can into your vagina (See **Diagram 5**). There is no danger of the ring being pushed too far up in the vagina or getting lost.

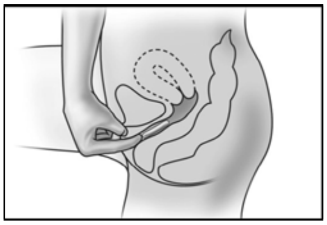

DIAGRAM 5

The ring should now be in your upper vagina (See **Diagram 6**).

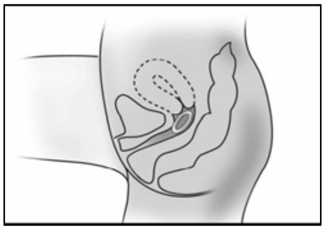

DIAGRAM 6

7. Wash your hands when you are done.

TO REMOVE THE VAGINAL RING:

1. Wash and dry your hands.
2. Choose the position that is most comfortable for you (See **Diagrams 1a** and **1b**).
3. Put a finger into your vagina and hook it through the ring. (See **Diagram 7**)

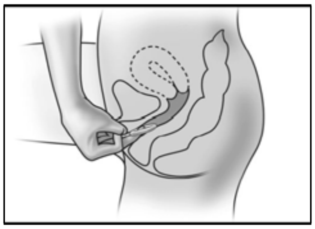

DIAGRAM 7

4. Gently pull downwards and forwards to remove the ring.
5. Wrap the used ring in tissue or toilet paper and give it to the clinic staff for disposal.
6. Wash your hands.

IMPORTANT INFORMATION

- If possible, you should try not to remove the ring for the entire 12 week period of the Vaginal Ring regimen except as directed during study visits. If the ring accidentally comes out of your vagina before your next clinic visit, e.g., during sex, clean it with warm water and put it back in your vagina.
- If you have any problems putting the ring back in your vagina, call or come to the clinic.

APPENDIX E: DIVISION OF AIDS (DAIDS) TABLE FOR GRADING SEVERITY OF ADULT ADVERSE EXPERIENCES FOR THE HIV PREVENTION TRIALS NETWORK

ABBREVIATIONS: **Abbreviations utilized in the Table:**

**ULN = Upper Limit of Normal**	**LLN = Lower Limit of Normal**
**R_x = Therapy**	**Req = Required**
**Mod = Moderate**	**IV = Intravenous**
**ADL = Activities of Daily Living**	**Dec = Decreased**

ESTIMATING SEVERITY GRADE

For abnormalities NOT found elsewhere in the Toxicity Table use the scale below to estimate grade of severity:

**GRADE 1**	**Mild**	Transient or mild discomfort (< 48 hours); no medical intervention/therapy required
**GRADE 2**	**Moderate**	Mild to moderate limitation in activity - some assistance may be needed; no or minimal medical intervention/therapy required
**GRADE 3**	**Severe**	Marked limitation in activity, some assistance usually required; medical intervention/therapy required, hospitalizations possible
**GRADE 4**	**Life-threatening**	Extreme limitation in activity, significant assistance required; significant medical intervention/therapy required, hospitalization or hospice care probable.

SERIOUS OR LIFE-THREATENING AEs

ANY clinical event deemed by the clinician to be serious or life-threatening should be considered a grade 4 AE. Clinical events considered to be serious or life-threatening include, but are not limited to: **seizures, coma, tetany, diabetic ketoacidosis, disseminated intravascular coagulation, diffuse petechiae, paralysis acute psychosis, severe depression.**

DAIDS TABLE FOR GRADING SEVERITY OF ADULT ADVERSE EXPERIENCES

**PARAMETER**	GRADE 1 MILD	GRADE 2 MODERATE	GRADE 3 SEVERE	GRADE 4 POTENTIALLY LIFE THREATENING
**HEMATOLOGY**				
Hemaglobin	9.5 g/dL – 10.5 g/dL	8.0 g/dL – 9.4 g/dL	7.9 g/dL – 6.5 g/dL	<6.5 g/dL
Absolute Neutrophil Count	1000 – 1500/mm^3	750 – 999/mm^3	500 - 749/mm^3	<500/mm^3
WBC	>13,000	>15,000	>20,000	>30,000 or <1,000
Percent Polys + Bands	>80%	90%	≥95%	---------
Platelets	100,000 – 120,000/mm^3	75,000 – 99,999/mm^3	50,000 – 74,999/mm^3	20,000 – 49,999/mm^3
CD4 Counts<br>Uninfected<br>Infected	300 - 400/mm^3<br><300 or <20%	<300/mm^3<br><200 or <18%	<200/mm^3<br><100 or <15%	<100/mm^3<br><50 or <12%
Fibrinogen	100-200 mg/dl OR 400-600 mg/dl	<100 mg/dl OR >800mg/dl	<50 mg/dl OR associated with gross bleeding OR associated with disseminated coagulation	---------
Prothrombin Time (PT)	>1.0 – 1.24 x ULN	>1.25 – 1.49 x ULN	>1.4 – 3.0 x ULN	>3.0 x ULN
PTT	>1.0 – 1.66 x ULN	>1.66 – 2.33 x ULN	>2.33 – 3.0 x ULN	>3.0 x ULN
**CHEMISTRIES**				
CPK	≥4 ULN	≥6 ULN	≥10 ULN	≥20 ULN
Creatinine	>1.0 – 1.5 x ULN	>1.5 – 1.9 x ULN	>2.0 – 6.0 x ULN	>6. x ULN
SODIUM<br>Hyponatremia<br>Hypernatremi	130 – 135 meq/L<br>146 – 150 meq/L	123 – 129 meq/L<br>151 – 157 meq/L	116 – 122 meq/L<br>158 – 165 meq/L	<116 meq/L<br>>165 meq/L

DAIDS TABLE FOR GRADING SEVERITY OF ADULT ADVERSE EXPERIENCES

**PARAMETER**	GRADE 1<br>MILD	GRADE 2<br>MODERATE	GRADE 3<br>SEVERE	GRADE 4<br>POTENTIALLY<br>LIFE THREATENING
POTASSIUM				
Hyperkalemia	5.0 – 5.5 meq/L	5.6 – 6.0 meq/L	6.1 – 6.5 meq/L	>6.6 meq/L
Hypokalemia	3.2 – 3.4 meq/L	3.0 – 3.1 meq/L	2.5 – 2.0 meq/L	<2.5 meq/L
PHOSPHATE				
Hypophosphalemia	2.0 – 2.4 mg/dL	1.5 – 1.0 mg/Dl	1.0 – 1.4 mg/dL	<1.0 mg/Dl
CALCIUM				
Hypocalcemia	7.8 – 8.4 mg/dL	7.0 – 7.7 mg/dL	6.1 – 6.9 mg/dL	<6.1 mg/dL
Hypercalcemia	10.6 – 11.5 mg/dL	11.6 – 12.5 mg/dL	12.6 – 13.5 mg/dL	>13.5 mg/Dl
MAGNESIUM				
Hypomagnesemia	1.2 – 1.4 meq/L	0.9 – 1.1 meq/L	0.6 – 0.8 meq/L	<0.6 meq/L
BILIRUBIN				
Hyperbilirubinemia	>1.0 – 1.5 x ULN	>1.5 – 2.5 x ULN	>2.5 – 5 x ULN	>5 x ULN
GLUCOSE				
Hypoglycemia	55 – 64 mg/dL	40 – 54 mg/dL	30 – 39 mg/dL	<30 mg/dL
Hyperglycemia (nonfasting and no prior diabetes)	116 – 160 mg/dL	161 – 250 mg/dL	251 – 500 mg/dL	>500 mg/dL
Triglycerides	-------------	400 – 750 mg/dL	751 – 1200 mg/dL	>1200 mg/dL
URIC ACID<br>Hyperuricemia	7.5 – 10.0 mg/dL	10.1 – 12.0 mg/dL	12.1 – 15.0 mg/dL	>15.0 mg/Dl

DAIDS TABLE FOR GRADING SEVERITY OF ADULT ADVERSE EXPERIENCES

**PARAMETER**	GRADE 1<br>MILD	GRADE 2<br>MODERATE	GRADE 3<br>SEVERE	GRADE 4<br>POTENTIALLY<br>LIFE THREATENING
LIVER TRANSAMINASE (LFTs)				
AST (SGOT)	1.25 – 2.5 x ULN	>2.5 – 5.0 x ULN	>5.0 – 10.0 x ULN	>10.0 x ULN
ALT (SGPT)	1.25 – 3.0 x ULN	>3.0 – 5.0 x ULN	>5.0 – 10.0 x ULN	>10.0 x ULN
GGT	1.25 – 2.5 x ULN	>2.5 – 5.0 x ULN	>5.0 – 10.0 x ULN	>10.0 x ULN
Alk Phos	1.25 – 2.5 x ULN	>2.5 – 5.0 x ULN	>5.0 – 10.0 x ULN	>10.0 x ULN
PANCREATIC ENZYMES				
Amylase	>1.0 – 1.5 x ULN	>1.5 – 2.0 x ULN	>2.0 – 5.0 x ULN	5.0 x ULN
Pancreatic amylase	>1.0 – 1.5 x ULN	>1.5 – 2.0 x ULN	>2.0 – 5.0 x ULN	5.0 x ULN
Lipase	>1.0 – 1.5 x ULN	>1.5 – 2.0 x ULN	>2.0 – 5.0 x ULN	5.0 x ULN
**CARDIOVASCULAR**				
Cardiac Arrhythmia	-----------	Asymptomatic; transient dysrhythmia, No R_x req	Recurrent/persistent dysrhythmia; symptomatic R_x req	Unstable dysrhythmia; hospitalization and R_x req
Hypertension	Transient, increase >20 mm Hg diastolic BP, no R_x req.	Recurrent; chronic increase >20 mm Hg diastolic BP; R_x req.	Acute R_x req; outpatient OR hospitalization possible	Hospitalization req OR en organ damage
Hypotension	Transient orthostatic hypotension with heart rate increased by >20 beats/min OR decreased by <10 mm Hg systolic BP, no R_x req.	Symptoms OR BP decreased by <20 mm HG systolic, correctable with oral fluid $R_{x.}$	IV fluid req. OR hospitalization	Mean arterial pressure <60 mm Hg. OR end organ damage, OR shock, vasopressor R_x req.
Pericarditis	Minimal effusion	Mild/mod asymptomatic effusion, no $R_{x.}$	Symptomatic effusion, pain, EKG changes.	Tamponade OR pericardiocentesis OR surgery req.
Hemorrhage, blood loss	-------------------	Mildly symptomatic no R_x req.	Gross blood loss OR 1-2 units transfused	Massive blood loss OR >2 units transfused.

DAIDS TABLE FOR GRADING SEVERITY OF ADULT ADVERSE EXPERIENCES

PARAMETER	GRADE 1 MILD	GRADE 2 MODERATE	GRADE 3 SEVERE	GRADE 4 POTENTIALLY LIFE THREATENING
**GASTROINTESTINAL**				
Nausea	Mild OR transient, reasonable intake maintained	Mod discomfort OR intake decreased for <3 days	Severe discomfort OR minimal intake for ≥ 3 days	Hospitalization req
Vomiting	Mild OR transient 2-3 episodes pe day OR mild vomiting lasting < 1 week	Mod OR persistent 4-5 episodes per day; OR vomiting lasting ≥ 1 week	Severe vomiting of all food/fluids in 24 hrs OR orthostatic hypotension OR IV R_x req.	Hypotensive shock OR severe electrolyte imbalance
Diarrhea	Mild OR transient; 3-4 loose stools per day OR mild diarrhea < 1 week	Mod OR persistent; 5-10 loose stools per day OR diarrhea lasting ≥ 1 week	> 10 loose stools/day, bloody diarrhea; OR orthostatic hypotension OR electrolyte imbalance, >2 L IV fluid required	Hypotensive shock OR severe electrolyte imbalance
Oral Discomfort/Dysphagia	Mild discomfort, no difficulty swallowing	Difficulty swallowing but able to eat and drink	Unable to swallow solids	Unable to drink fluids; IV fluids req.
Constipation		Moderate abdominal pain 78 hours with impaction require output prescription	Requiring disimpaction or hospital treatment	Distention with vomiting OR obstipation
**RESPIRATORY**				
Cough (for aerosol studies)	Transient, no R_x	Treatment associated cough; inhaled bronchodilator	Uncontrolled cough; systemic R_x req.	-------------------
Bronchospasm (acute)	Transient; no R_x ; FEV1 or peak flow reduced to 70%-80%	R_x Req; normalizes with bronchodilator; FEV1 or peak flow 50%-60%	No normalization with bronchodilator; FEV1 or peak flow 25% - 49%, retractions	Cyanosis; FEB1 or peak flow <25% OR intubated
Dyspnea	Dyspnea on exertion	Dyspnea with normal activity	Dyspnea at rest	Dyspnea requiring O_2 therapy

DAIDS TABLE FOR GRADING SEVERITY OF ADULT ADVERSE EXPERIENCES

PARAMETER	GRADE 1<br>MILD	GRADE 2<br>MODERATE	GRADE 3<br>SEVERE	GRADE 4<br>POTENTIALLY<br>LIFE THREATENING
**NEUROLOGIC**				
Neuro-cerebellar	Slight incoordination OR dysdiadochokinesia	Intention tremor OR dysmetria OR slurred speech OR nystagmus	Ataxia requiring assistance to walk or arm incoordination interfering with ADLs	Unable to stand
Neuro-psych/mood	-----------------	--------------------	Severe mood changes requiring medical intervention; Suicidal ideation	Acute psychosis req hospitalization; Suicidal gesture/attempt
Paresthesia (burning, tingling, etc.)	Mild discomfort; no R_x req.	Mod discomfort; non-narcotic analgesia required	Severe discomfort; OR narcotic analgesia req with symptomatic improvement	Incapacitating; OR not responsive to narcotic analgesia
Neuro-motor	Mild weakness in muscle of feet but able to walk and/or mild increase or decrease in reflexes	Mod weakness in feet (unable to walk on heels and/or toes), mild weakness in hands, still able to do most hand tasks and/or loss of previously present reflex or development of hyperreflexia and/or unable to do deep knee bends due to weakness	Marked distal weakness (unable to dorsiflex toes or foot drop, and mod proximal weakness e.g., in hands interfering with ADLs and/or requiring assistance to walk and/or unable to rise from chair unassisted	Confined to bed or wheel chair because of muscle weakness
Neuro-sensory	Mild impairment (decreased sensation, e.g., vibratory, pinprick, hot/cold in great toes) in focal area or symmetrical distribution	Mod impairment (mod decreased sensation, e.g., vibratory, pinprick, hot/cold to ankles) and/or joint position or mild impairment that is not symmetrical	Severe impairment (decreased or loss of sensation to knees or wrists) or loss of sensation of at least mod degree in multiple different body areas (i.e., upper and lower extremities)	Sensory loss involves limbs and trunk

DAIDS TABLE FOR GRADING SEVERITY OF ADULT ADVERSE EXPERIENCES

PARAMETER	GRADE 1 MILD	GRADE 2 MODERATE	GRADE 3 SEVERE	GRADE 4 POTENTIALLY LIFE THREATENING
**MUSCULOSKELETAL**				
Arthralgia/Arthritis	Arthralgia	Arthralgia with joint effusion or moderate impairment of activity	Frank arthritis with or without effusion OR resulting in severe impairment of activity	-----------------------------
Myalgia	Myalgia without limitation of activity	Muscle tenderness at other than injection site or with moderate impairment of activity	Frank myonecrosis OR with severe impairment of activity	-------------------------------
**CUTANEOUS**				
Rash/Dermatitis	Erythema, pruritus	Diffuse maculopapular rash OR dry desquamation	Vesiculation OR moist desquamation OR ulceration	ANY ONE: mucous membrane involvement, suspected Stevens-Johnson (TEN), erythema multiforme, necrosis req surgery, exfoliative dermatitis
Local Reaction	Erythema OR induration <15 x 15 cm (225 cm^2)	Erythema, induration, or Edema >15 x 15 cm (225 cm^2)	Ulceration OR super infection OR phlebitis	Necrosis of the skin
**URINALYSIS**				
Proteinuria				
Random urine	1+	2 – 3+	4+	Nephrotic syndorme
24 hour urine	200 mg-1 g loss/day OR <0.3% OR <3 g/l	1 – 2 g loss/day OR 0.3 – 1.0% OR 3 – 10 g/l	2 – 3.5 g loss/day OR >1.0% OR >10 g/l	Nephrotic syndrome OR >3.5 g loss/day
Hematuria	Microscopic only ≤ 10 rbc/hpf	> 10 rbc/hpf	Gross, with or without clots OR RBC casts	Obstructive OR transfusion req

DAIDS TABLE FOR GRADING SEVERITY OF ADULT ADVERSE EXPERIENCES

**PARAMETER**	GRADE 1<br>MILD	GRADE 2<br>MODERATE	GRADE 3<br>SEVERE	GRADE 4<br>POTENTIALLY<br>LIFE THREATENING
**MISCELLANEOUS**				
Fever<br>Oral >12 hours	37.7 – 38.9C<br>(100.0 – 101.5F)	39.0 –39.5C<br>(101.6 – 102.9F)	39.6 – 40.5C<br>(103 – 105F)<br>or max temp of 103.5F	>40.5C (205F)
Headache	Mild; no R_x req, OR non-narcotic analgesia R_x	Mod; OR responds to initial narcotic R_x	Severe; intractable; OR requiring repeated narcotic R_x	Requiring hospitalization, associated with neurologic, respiratory or cardiovascular abnormalities
Allergic Reaction	Pruritus without rash at injection site	Localized urticaria at injection site	Generalized urticaria angloedema	Anaphylaxis
ADL*	Normal activity reduced <48 hours	Normal activity reduced 25-50% >48 hours	Normal activity reduced >50%; cannot work > 48 hours	Unable to care for self
EYE		Mild pain, visual changes, conjunctival erythema, abnormal slit lamp	Loss of vision, clinically diagnosed uveitis, mod-severe pain, glaucoma	-----------------------------

APPENDIX F: CLINICAL MANAGEMENT OF GENITAL DIAGNOSES

Diagnosis	Evaluation	Treatment Action and Follow-Up	Investigational Product Use
**(Severe) Cutaneous Reaction**<br>Erythema and/or edema covering a localized area more than 50% of vulvar surface, or >50% of combined vulvar and perianal surface, or >50% of combined vaginal and cervical surface.	Clinical history for hypersensitivity reaction (assess genital/other products used), speculum and pelvic examination.<br><br>Perform pH and wet preparations for vaginitides.	Topical or systemic antihistamine and or anti-inflammatory agent. Follow-<br><br>up in 5-7 days.	Remove investigational product and permanently discontinue use.
**Ulceration**<br>Deep epithelial disruption.	Clinical history (HSV?), speculum and pelvic examination.<br><br>Culture lesion for HSV.<br><br>Syphilis serology.<br><br>pH and wet mount preparations as indicated.	Treatment as indicated.<br><br>If syphilis screen is positive, obtain confirmatory test and treat accordingly.<br><br>If negative for HSV and syphilis, consider *H. ducreyi* (chancroid) infection.<br><br>Re-evaluate in 5-7 days; consider biopsy if lesion persists.	Temporarily discontinue until resolution of lesion.
**Abrasion/ Peeling**<br>Superficial epithelial disruption of vulvar, vaginal, or perianal areas.	Clinical history (trauma?), speculum and pelvic exam.<br><br>pH and wet mount preparations as indicated.	Counsel regarding factors for trauma if present.<br><br>Re-evaluate in 72 hours by speculum exam.<br><br>If progression, evaluate as for ulceration.	Temporarily discontinue use.<br><br>If condition resolves, restart product use.
**Vulvovaginitis**	Clinical history, speculum and pelvic examination.<br><br>pH and wet mount preparations for vaginitides.	Treat candidiasis, bacterial vaginosis, trichomoniasis if present.	If symptomatic, temporarily discontinue product use; may resume product use after 3 days of treatment if vulvovaginitis resolved.<br><br>If asymptomatic, initiate treatment and continue product use.

Diagnosis	Evaluation	Treatment Action and Follow-Up	Investigational Product Use
**Cervicitis**	Clinical history, speculum and pelvic examination.<br><br>Obtain specimens for DNA tests for *N. gonorrhea* and *C. trachomatis*.<br><br>pH and wet mount preparations as indicated.	Presumptive treatment at discretion of clinician.<br><br>Recall for treatment for GC and/or Chlamydia as needed.<br><br>Treat vaginitis if present.	If presumptive treatment provided, continue product use; otherwise temporarily discontinue product if clinical determination is not made or awaiting test results.
**Abnormal Uterine Bleeding**	Clinical history, speculum and pelvic examination.<br><br>Classify according to etiology (dysfunctional uterine bleeding, pregnancy, bleeding diathesis, benign or neoplastic anatomic lesion, inflammatory conditions, laceration or trauma, contraceptive method, systemic disease, other).<br><br>Pregnancy test, cultures, pH and wet preparations as indicated.	Treatment as indicated.	If pregnant or neoplastic lesion is present, permanently discontinue product use.<br><br>Otherwise, temporarily discontinue product use and re-evaluate at 7 day intervals as indicated. May resume product use when condition resolved.
**Petechiae (Vulvar, vaginal or cervical) <2-3mm**	Clinical history, speculum and pelvic examination.<br><br>pH and wet mount preparations as indicated.	If tests negative, observe and re-evaluate at least every 72 hours until resolved.<br><br>Treat positive results of wet mount preparations as indicated.	Continue product use or temporarily discontinue use if evidence of progression.
**Ecchymosis (Vulvar, vaginal or cervical >3 mm**	Clinical history, speculum and pelvic examination.<br><br>pH and wet preparations as indicated.	If tests negative, observe and re-evaluate at least every 72 hours until resolved.<br><br>Treat positive results of wet mount preparations as indicated.	Continue product use, or temporarily discontinue use if evidence of progression.

Screening ID Number **Subject Initials**

APPENDIX G: SAMPLE <u>SCREENING</u> CONSENT FORM TEMPLATE

A SAFETY AND ACCEPTABILITY STUDY OF A VAGINAL RING MICROBICIDE DELIVERY METHOD FOR THE PREVENTION OF HIV INFECTION IN WOMEN

Insert study site/host country and the International Partnership for Microbicides (IPM) are conducting a research study in healthy sexually active women who will wear a vaginal ring and be asked their opinion about its use. Vaginal rings have previously been used to deliver medicines to prevent pregnancy and to treat menopause. Research is being conducted to see if the ring can be used to deliver a vaginal microbicide to prevent HIV infection in women. Vaginal microbicides are medicines being developed to prevent HIV infection. **<u>The ring being used in this particular study contains no medicine at all and will not protect you from HIV or any other sexually transmitted infection.</u>** This study (called the "ring study") is being conducted to see if the ring delivery method is acceptable and safe.

You are not being asked to join in the ring study right now. You are being asked to join in a screening study to see if you meet the requirements to join the ring study. You will be given information about the ring study and told what it means to join the study. If you meet the requirements and are willing to take part in the ring study, you will be given the chance to join later.

Before you decide if you want to join this 30 day screening study or not, we want to explain the study, its risks, its potential benefits, and what you will be asked to do. You may ask questions as we discuss the study, so that you understand what the study is about. It is important you know the following:

- Joining the screening study is your decision (voluntary) and not up to anyone else.
- You will not lose any of your routine medical care benefits if you decide not to join the screening study.
- You may join the screening study and then change your mind and leave the screening study at any time.

If you agree to join this screening study, we will ask you to sign and date this consent form. We will give you a copy of this form to keep.

<u>HOW WILL THE SCREENING STUDY WORK?</u>

After you sign this consent form, the following will happen:

1. **Interview** – You will be asked questions about yourself including your health, medical history, medicines taken, menses information, and where we can contact you. You will also be asked some questions to see if you are eligible for the ring study.
2. **Counseling** – You will meet in private with a counselor who will talk with you and provide information about how to prevent or reduce your risk of infection with HIV or other sexually transmitted infections. You will be provided condoms. The counselor will talk to you before your HIV test and after your test results are ready. The counselor will also discuss the most

Screening ID Number **Subject Initials**

reliable type of birth control method for you to use during the ring study and will provide the birth control method to you or refer you to a family planning clinic to obtain contraceptives. **You will not be allowed to use a vaginal barrier method, for example, a diaphragm or cervical cap.**

3. **HIV Testing** – Your mouth will be swabbed to test for HIV. If the test shows you have HIV, you cannot join the ring study and no more procedures will be done. However the nurse or doctor will use a needle to prick your finger and, if necessary, the nurse or doctor may also need to take a small amount of blood from your arm (less than 1 teaspoon**)** to confirm the test results. You will be referred to local health facilities for social support or other medical services as needed.
4. **Pregnancy Testing** – You will give a sample of your urine to test if you are pregnant. If you are pregnant, you cannot join the ring study and no more procedures will be done. You will be referred to the local prenatal clinic for support services.
5. **Physical Exam, Pelvic Exam, and Lab Testing** – You will have a physical exam and a pelvic exam to check inside your vagina for any infections, ulcers or sores. The nurse or doctor will use a swab to take samples from your vagina which will be sent to a lab to check for genital infections. You will also have samples taken for a Pap smear to check for cervical cancer. (If you are menstruating at this screening visit, the pelvic exam and collection of vaginal samples will be rescheduled to another visit after completion of your menses). If you have symptoms or findings of a genital infection, you will be provided treatment, if available, or referred to local health facilities for treatment as needed. If your exams and lab tests show you are still eligible for the study, you will be given a date to return to clinic within 30 days for the enrollment visit. (NOTE: You should NOT be menstruating at the enrollment visit. If you are, tell the study staff and the visit will be rescheduled).

The visit will take about **X amount time** to complete. You will receive **X amount** for your time and travel expenses.

IF I AM STILL ELIGIBLE FOR THE RING STUDY, WHAT HAPPENS?
At the time of the enrollment visit (within 30 days from the screening visit), you will first undergo some pre-enrollment procedures as follows:

1. **Interview** – You will be asked about any medical problems and/or medicines you have taken since your last visit, menses information, and where we can contact you
2. **Counseling** – You will meet in private with a counselor who will talk with you and provide information about how to prevent or reduce your risk of infection with HIV or other sexually transmitted infections. You will be given condoms. The counselor will also discuss the most reliable type of birth control method for you to use during the ring study and will provide the birth control method to you or refer you to a family planning clinic to obtain contraceptives. **You will not be allowed to use a vaginal barrier method, for example, a diaphragm or cervical cap**.

Screening ID Number **Subject Initials**

3. **Pregnancy Testing** – You will give a sample of your urine to test if you are pregnant. If you are pregnant, you cannot join the ring study and no more procedures will be done. You will be referred to the local prenatal clinic for support services.
4. **Pelvic Exam & Colposcopy** – If your pregnancy test is negative, you will have a pelvic exam and colposcopy to check inside your vagina for any infections, ulcers or sores. A colposcope is an instrument which uses a magnifying lens to examine your vaginal tissue. If your exam shows that you still have infections, ulcers or sores 30 days after your initial screening visit, you will be provided treatment, if available, or referred to local health facilities for treatment as needed but you will not be eligible for the ring study.

If your exam shows that you are still eligible, you will be invited to join in the ring study right away, and be requested to sign a separate study consent form. However, you will be under no obligation to join in ring study and may freely refuse to do so. Regardless of whether you decide to join the ring study or not, the visit will take about **X amount time** to complete and you will receive **X amount** for your time and travel expenses.

By signing this consent form, you have been told that if you are eligible to return for the enrollment visit but do not show up, you have given permission for clinic staff to contact you.

WHAT ARE THE POTENTIAL RISKS OF THIS SCREENING STUDY?
There are no serious risks associated with joining in this screening eligibility study. You may feel discomfort during the pelvic exam and/or colposcopy, and there is a very small risk of injury to the lining inside your vagina from the speculum. You may become embarrassed, worried, or anxious during the pelvic or physical exams, or when discussing your health, sexual behaviors, or as a result of being tested for HIV infection and counseled. You may become worried or anxious while waiting for results of your tests for HIV or other infections. If you have a genital infection, you might experience problems with your partner(s). If you have a positive HIV test and need to have blood drawn to confirm your result, you may feel discomfort from the finger prick or when blood is drawn and you may feel dizzy or faint and may later have a bruise or swelling (and rarely, an infection) where the needle goes in your finger or arm.

WHAT ARE THE POTENTIAL BENEFITS OF THIS SCREENING STUDY?
You will have your health evaluated, receive medical exams and a lab test to check for genital infections and offered treatment or referred outside of the study for medical care free of charge.

WHAT ARE THE COSTS OF THE SCREENING STUDY?
There is no cost to you for taking part in the study. You will be reimbursed **X amount** for your time and travel.

WHO WILL SEE MY PERSONAL & MEDICAL INFORMATION?
We will do everything we can to protect your privacy. You will be assigned a code number which will be used on all information collected about you (instead of your name) on this study. A master list with your name and code will be kept under lock and key at the clinic where you

Screening ID Number **Subject Initials**

are enrolled. Your name will never be used in any publication or presentation about this study. However people or groups that may review your records include your country's regulatory agency and other national regulatory agencies, e.g., the U.S. Food and Drug Administration (FDA), Ethics Committees, study monitors, the manufacturing company or the Sponsor of the study (IPM).

WHAT HAPPENS IF I AM INJURED?

If you become ill or injured as a result of taking part in the screening study, you will receive medical treatment free of charge. The study staff also will tell you where you can get additional treatment, if needed. We will pay your reasonable medical costs for treatment of any illness or injury that is associated with your joining the study..

WHAT IF I HAVE QUESTIONS OR PROBLEMS?

If you have any questions, you can contact a member of the clinic staff or Dr.______________ Tel__________________. If at any time you have any questions regarding your rights as a subject in a research study, you may contact___________________.

SIGNATURE TO SCREEN FOR THE IPM 011 STUDY:

I have read this consent form (or had it explained to me), and all of my questions have been answered to my satisfaction. I know that I can refuse to join this screening study, or if I agree to join I can drop out of the screening study at any time without losing any benefits or services to which I am entitled. After signing below, I will receive a copy of this consent form. My signature (or thumbprint or mark) below confirms that I freely agree to **SCREEN** for this study.

___________________________________	___________________________________
Volunteer's Name (print)	Volunteer's Signature/Thumbprint/Mark & Date
___________________________________	___________________________________
Name of Staff Conducting Consent Session (print)	Staff Signature & Date
___________________________________	___________________________________
Investigator or Co-investigator (print)	Investigator or Co-investigator Signature & Date
___________________________________	_____________________________
Witness' Name (print) (AS APPRO PRIATE)	Witness's Signature & Date

Subject ID Number

Subject Initials

APPENDIX H: SAMPLE <u>STUDY</u> CONSENT FORM TEMPLATE

A SAFETY AND ACCEPTABILITY STUDY OF A VAGINAL RING MICROBICIDE DELIVERY METHOD FOR THE PREVENTION OF HIV INFECTION IN WOMEN

Insert study site/host country and the International Partnership for Microbicides (IPM) are conducting a research study in healthy sexually active women who will wear a vaginal ring and be asked their opinion about its use. Vaginal rings have previously been used to deliver medicines to prevent pregnancy and to treat menopause. Research is being conducted to see if the ring can be used to deliver a vaginal microbicide to prevent HIV infection in women. Vaginal microbicides are medicines being developed to prevent HIV infection. <u>**The ring being used in this particular study contains no medicine at all and will not protect you from HIV or any other sexually transmitted infection.**</u> This study (called the "ring study") is being conducted to see if the ring delivery method is acceptable and safe. This consent form will give you information to help you decide if you would like to join the ring study.

Before you decide if you want to join this ring study or not, we want to explain the study, its risks, its potential benefits, and what you will be asked to do. You may ask questions as we discuss the ring study, so that you understand what the study is about. It is important you know the following:

- Joining the ring study is your decision (voluntary) and not up to anyone else.
- You will not lose any of your routine medical care benefits if you decide not to join the ring study.
- You may join the ring study and then change your mind and leave the study at any time.

If you agree to join this ring study, we will ask you to sign and date this consent form. We will give you a copy of this form to keep.

<u>WHAT IS THE STUDY FOR?</u>

The main study purpose is to find out your opinions about using a vaginal ring over a 12 week period. We also want to look at any vaginal problems you may experience while wearing the ring for a 12 week period compared to when you are not wearing the ring for the other 12 week period.

<u>WHO WILL BE IN THE STUDY?</u>

230 healthy women from 5 sites in Kenya, South Africa and Tanzania, will join this study which is expected to last approximately 9 months total, or perhaps longer, depending on how long it takes for 230 women to join the study.

<u>WHAT IS THE VAGINAL RING?</u>

The vaginal ring used in this study is an off-white, soft, flexible ring <u>which contains NO study medication</u>. Studies have shown that the ring itself appears safe, non-toxic and non-irritating.

Subject ID Number **Subject Initials**

WHAT IS THE STUDY REGIMEN?

There are two 12 week regimens in this study, the Vaginal Ring regimen and the Observational Safety regimen. During the Vaginal Ring regimen, women will wear the ring for 12 continuous weeks and be followed at the clinic. During the Observational Safety regimen, women will also be followed at the clinic but not wear a ring. Half of the women will start on the Vaginal Ring regimen the first 12 weeks and then "crossover" or switch to the Observational Safety regimen the last 12 weeks. The other half of the women will be on the exact opposite schedule and start on the Observational Safety regimen the first 12 weeks and then crossover to the Vaginal Ring regimen the last 12 weeks.

If you join the study, you will be put into 1 of the 2 study groups by chance, Group A or Group B. Group A will wear the ring the first 12 weeks and then not wear the ring the last 12 weeks. Group B will not wear the ring the first 12 weeks and then wear the ring the last 12 weeks. Everyone who joins the study will have an equal chance of being in either group. Neither you nor the study staff chooses the group you are in.

CROSSOVER

	**FIRST 12 WEEKS**		**LAST 12 WEEKS**
Group A	Vaginal Ring	→	Observational Safety (No Ring)
Group B	Observational Safety (No Ring)	→	Vaginal Ring

HOW LONG IS THE STUDY?

There are 9 scheduled visits (including the enrollment visit) which take place over a 24 week period. After enrolling into the study, you will return to clinic 2, 4, 8, and 12, 14, 16, 20 and 24 weeks after enrollment. If you have any genital symptoms or pelvic exam findings at the crossover visit (12 weeks into the study), you will come back to clinic for an extra study visit and your study schedule will be stretched out 2 more weeks (for a total participation time of 26 weeks). Most visits will take about **X** hours to complete but sometimes a study visit may take a little longer. You may need to come back to clinic for additional visits if you have any medical problems or concerns.

In addition to these study visits, at a later time after you have completed the Vaginal Ring regimen, you may be invited to join in a separate focus group discussion composed of approximately 20 women to find out your opinions about the vaginal ring. If you decide to join in this separate focus group discussion, the procedures will first be explained to you and you will be given a separate consent form to sign. Joining this separate focus group discussion is

Subject ID Number

Subject Initials

voluntary. If you decide you do not want to join in this separate group discussion, you will not lose any of your routine medical care benefits and you can still also participate in the main IPM 011 ring study.

Also, at a later time after you have completed the Vaginal Ring regimen, you will be asked if you would like your male partner to be invited to participate in an individual interview to find out his opinions about the vaginal ring including how he feels about your using the ring and how he feels about you joining in this study. If he decides to participate in an individual interview, the procedures will first be explained to him and he will be given a separate consent form to sign. His participation in an individual interview is voluntary. If he decides he does not want to participate in the individual interview, there is no penalty. You will not lose any of your routine medical care benefits and you can still participate in the main IPM 011 ring study.

ONCE I AM CONSIDERED ELIGIBLE FOR THE RING STUDY, WHAT HAPPENS?
After you have passed all the screening and pre-enrollment procedures described earlier in the Screening Consent form and have signed this study consent form, you will be enrolled into the study and put into either Group A or Group B. The following describes what will happen at specific visits depending on which group you are in. (NOTE: the procedures below do not necessarily occur in the order listed):

GROUP A – (VAGINAL RING FIRST 12 WEEKS & OBSERVATIONAL SAFETY LAST 12 WEEKS)

- **Vaginal Ring Insertion – Enrollment**
  Study staff will give you a vaginal ring and show you how to insert the ring yourself and you will do this at the clinic with staff help, if needed. You will have a brief inspection to confirm the ring is in place.
- **Adherence Counseling – Enrollment & 2, 4, and 8 Weeks After Enrollment**
  You will be counseled to wear the ring for the 12 week period and not to take it out at anytime except when directed at study visits. You will be given instructions on how to re-insert the ring if it comes out accidentally. You will also be told not to use vaginal products or other objects.
- **Vaginal Ring Removal – 12 Weeks After Enrollment**
  The study staff will show you how to remove the ring yourself and you will do this at the clinic with staff help if needed. The staff will dispose of the ring at the clinic.
- **Questionnaire – Enrollment & 4, 8, 12, and 24 Weeks After Enrollment**
  Depending on the visit, you will be asked questions regarding your opinion about using a vaginal ring, what you know about these types of rings, whether you have used one before, and if you would use a ring to prevent HIV infection, ease of use, comfort, cleanliness, and use during sex as well as your partner’s reactions. You will also be asked questions about sexual behavior, condom use, and whether you would use this type of vaginal ring if it contained a vaginal microbicide approved for protection against HIV infection.

Subject ID Number

Subject Initials

- **Pelvic Exam – 2, 8, 12, 14, 20, and 24 Weeks After Enrollment**
  You will have a pelvic exam to check inside your vagina for any problems. At 2 and 8 weeks after enrollment only, before you have the exam, the study staff will show you how to remove the ring yourself and you will do this with staff help if needed. After the exam, you will reinsert the ring yourself with staff help if needed.
- **Colposcopy & Lab Tests – 12 and 24 Weeks After Enrollment**
  In addition to the pelvic exam at these visits, the doctor will use a colposcope, which is an instrument with a magnifying lens, to check inside your vagina for any problems. Your vagina will be swabbed for samples and sent to a lab to check for genital infections.
- **Interview –2, 4, 8, 12, 14, 16, 20, and 24 Weeks After Enrollment**
  You will be asked about any medical problems and/or medicines you have taken since your last visit, menses information, and where we can contact you.
- **HIV & Contraceptive Counseling – 2, 4, 8, 12, 14, 16, 20, and 24 Weeks After Enrollment**
  You will meet in private with a counselor who will talk with you and provide information about how to prevent or reduce your risk of infection with HIV or other sexually transmitted infections. You will be given condoms. The counselor will also discuss the most reliable type of birth control method for you to use during the study and will provide the birth control method for you or refer you to a family planning clinic to obtain contraceptives. **You will not be allowed to use a vaginal barrier method, for example, a diaphragm or cervical cap.**
  At 12 and 24 weeks after enrollment only, the counselor will also talk to you before your HIV test and after your test results are ready.
- **HIV Testing – 12 and 24 Weeks After Enrollment**
  Your mouth will be swabbed to test for HIV.
- **Pregnancy Testing – 12 and 24 Weeks After Enrollment**
  You will give a sample of your urine to test if you are pregnant. NOTE: You will also have pregnancy testing during the study any time you miss a menstrual period.

NOTE: If you have any genital symptoms or findings from the pelvic exam 12 weeks after enrollment, you will be treated and asked to return to clinic 2 weeks later for another pelvic and colposcope exam. If you have no more symptoms or findings at this time, you will continue with your study schedule for the last 12 weeks of the study. If you still have symptoms or findings at this time, your participation in the study will end but you will be followed until your condition resolves or stabilizes.

You will be reminded to contact or return to clinic if you have any medical problems or concerns anytime during the study including if you accidentally expel or lose the vaginal ring during the Vaginal Ring regimen (first 12 weeks of the study). Each visit will take about **X amount time** to complete. You will receive **X amount** for your time and travel expenses.

Subject ID Number

Subject Initials

GROUP B – (OBSERVATIONAL SAFETY FIRST 12 WEEKS & VAGINAL RING LAST 12 WEEKS)

- **Pelvic Exam – 2, 8, 12, 14, 20, and 24 Weeks After Enrollment**
  You will have a pelvic exam to check inside your vagina for any problems. At 14 and 20 weeks after enrollment only, before you have the exam, the study staff will show you how to remove the ring yourself and you will do this with staff help if needed. After the exam, you will reinsert the ring yourself with staff help if needed.
- **Colposcopy & Lab Tests – 12 and 24 Weeks After Enrollment**
  In addition to the pelvic exam at these visits, the doctor will use a colposcope, which is an instrument with a magnifying lens, to check inside your vagina for any problems. Your vagina will be swabbed for samples and sent to a lab to check for genital infections.
- **Interview – 2, 4, 8, 12, 14, 16, 20, and 24 Weeks After Enrollment**
  You will be asked about any medical problems and/or medicines you have taken since your last visit, menses information, and where we can contact you.
- **HIV & Contraceptive Counseling – 2, 4, 8, 12, 14, 16, 20, and 24 Weeks After Enrollment**
  You will meet in private with a counselor who will talk with you and provide information about how to prevent or reduce your risk of infection with HIV or other sexually transmitted infections. You will be given condoms. The counselor will also discuss the most reliable type of birth control method for you to use during the study and will provide the birth control method for you or refer you to a family planning clinic to obtain contraceptives. **You will not be allowed to use a vaginal barrier method, for example, a diaphragm or cervical cap.** At 12 and 24 weeks after enrollment only, the counselor will also talk to you before your HIV test and after your test results are ready.
- **HIV Testing – 12 and 24 Weeks After Enrollment**
  Your mouth will be swabbed to test for HIV.
- **Pregnancy Testing – 12 and 24 Weeks After Enrollment**
  You will give a sample of your urine to test if you are pregnant. NOTE: You will also have pregnancy testing during the study any time you miss a menstrual period.
- **Vaginal Ring Insertion – 12 Weeks After Enrollment**
  Study staff will give you a vaginal ring and show you how to insert the ring yourself and you will do this at the clinic with staff help, if needed. You will have a brief inspection to confirm the ring is in place.
- **Adherence Counseling – 12, 14, 16, and 18 Weeks After Enrollment**
  You will be counseled to wear the ring for the 12 week period and not to take it out at anytime except when directed at study visits. You will be given instructions on how to re-insert the ring if it comes out accidentally. You will also be told not to use vaginal products or other objects.
- **Vaginal Ring Removal – 24 Weeks After Enrollment**
  The study staff will show you how to remove the ring yourself and you will do this at the clinic with staff help if needed. The staff will dispose of the ring at the clinic.

Subject ID Number

Subject Initials

- **Questionnaire – Enrollment & 12, 16, 20, and 24 Weeks After Enrollment**
  Depending on the visit, you will be asked questions regarding your opinion about using a vaginal ring, what you know about these types of rings, whether you have used one before, and if you would use a ring to prevent HIV infection, ease of use, comfort, cleanliness, and use during sex as well as your partner's reactions. You will also be asked questions about sexual behavior, condom use, and whether you would use this type of vaginal ring if it contained a vaginal microbicide approved for protection against HIV infection.

NOTE: If you have any genital symptoms or findings from the pelvic exam 12 weeks after enrollment, you will be treated and asked to return to clinic 2 weeks later for another pelvic and colposcope exam. If you have no more symptoms or findings at this time, you will continue with your study schedule for the last 12 weeks of the study. If you still have symptoms or findings at this time, your participation in the study will end but you will be followed until your condition resolves or stabilizes.

You will be reminded to contact or return to clinic if you have any medical problems or concerns anytime during the study including if you accidentally expel or lose the vaginal ring during the Vaginal Ring regimen (last 12 weeks of the study). Each visit will take about **X amount time** to complete. You will receive **X amount** for your time and travel expenses.

Regardless of whether you are in Group A or B, we will use the contact information you provide to remind you of scheduled visits. If you miss a visit, the study staff will try to contact you by phone or other methods. They will try to reach you at home or through family, friends or authorities. If they talk to these people, they will not tell them why they are trying to reach you.

ARE THERE ADDITIONAL STUDY REQUIREMENTS DURING THE STUDY?

Do not use products or objects which are inserted into the vagina such as tampons, cotton wool, rags, diaphragms, cervical caps, douches and drying agents while you are participating in the study.

WHAT IF I BECOME HIV-INFECTED DURING THE STUDY?

After you have enrolled in the study, if your mouth swab tests positive for HIV, the nurse or doctor will use a needle to prick your finger and, if necessary, the nurse or doctor may also need to take a small amount of blood from your arm (less than 1 teaspoon**)** to confirm the test results. You will be referred to local health facilities for social support or other medical services as needed. If you are confirmed to be infected, you will be offered treatment for HIV infection based on your medical needs according to your country's treatment guidelines. If those guidelines are unavailable, the guidelines of the World Health Organization (WHO) will be followed. **If national or local ARV treatment programs are not in place, IPM will pay for ARV treatment until such resources are available.**

Subject ID Number **Subject Initials**

WHAT IF I BECOME PREGNANT DURING THE STUDY?

Since we do not know whether the vaginal ring has any effect on pregnancy, or whether it can harm the fetus, pregnant women cannot join this study. If you are a woman able to become pregnant, you must have been taking oral contraceptive pills for at least 3 months or been on a long-acting birth control method like injectable Depo-Provera for 6 months prior to joining the study. You must agree to continue using birth control (which is not a vaginal barrier method like a diaphragm or cervical cap) during this study and you will have a pregnancy test during screening and 12 and 24 weeks after enrollment. You will also have pregnancy testing during the study any time you miss a menstrual period.

If you find out you are pregnant or you are confirmed to be pregnant during a clinic visit and you are in the Vaginal Ring regimen, you will immediately remove the ring and will no longer use it. If applicable, bring the ring back to the clinic for proper disposal. You will go through all your regularly scheduled procedures at the study visit according to the study regimen you are currently on, e.g., Vaginal Ring regimen or Observational Safety regimen. After this visit, you will return to clinic according to your study schedule for safety follow-up until completion of the last study visit 24 weeks after enrollment. You will receive referrals to prenatal clinics or other appropriate facilities.

WHAT HAPPENS IF I WANT TO WITHDRAW FROM THE STUDY EARLY?

If you choose not to continue in the study before it ends, you will be asked to return for one last study visit. You do not have to return for this visit. If you do return for this visit, study procedures performed at the last visit for the specific regimen you are in as described above will be performed.

WHAT ARE THE RISKS/DISCOMFORTS OF THIS STUDY?

Some of the risks and/or discomforts that you should consider before agreeing to take part in this study are described as follows. If you have any problems during the study, you should contact the study staff right away.

It is possible that you are allergic to the material (silicone rubber) used to make the vaginal ring. It is possible that you may experience local irritation or vaginal complaints as a result of using the ring. You may feel discomfort during the pelvic exam and/or colposcopy, and there is a very small risk of injury to the lining inside your vagina from the speculum. You may become embarrassed, worried, or anxious during the pelvic or physical exams, or when discussing your health, sexual behaviors, or HIV infection; or answering questions about use of the ring before, during or after sex. You may become worried or anxious while waiting for results of your tests for HIV and other infections. If you have a positive HIV test and need to have blood drawn to confirm your result, you may feel discomfort when the blood is drawn and may feel dizzy or faint and may later have a bruise or swelling (and rarely, an infection) where the needle goes in your arm.You might experience problems with your partner(s) if you have a genital infection and

Subject ID Number

Subject Initials

need to tell your partner(s). You might experience problems with your partner(s) related to your using (or attempting to use) the ring. You have been told your joining in this study may be associated with study-related discrimination. Discrimination may arise if you choose to tell family, friends, co-workers or others about you joining the study. You may suffer discrimination if others think you are at risk for HIV-1 infection or HIV-1 infected because you joined in the study. Our staff can advise you on dealing with such problems. We will make every effort to protect your privacy and confidentiality during the study and help you deal with any uncomfortable feelings or questions you may have

WHAT ARE THE BENEFITS OF THIS STUDY?
This study may be of no direct benefit to you. However, you or others may benefit in the future from information learned from this study about how vaginal rings may be used with topical microbicides to prevent HIV infection. You will have your health evaluated, receive medical exams and a lab test to check for genital infections and offered treatment or referred outside of the study for medical care free of charge.

WHAT IF THERE IS NEW INFORMATION ABOUT THIS STUDY?
During the course of the study, you will be told about any important new findings that may be beneficial or harmful to you and that might influence your willingness to continue joining in the study.

WHY WOULD I BE REMOVED FROM THE STUDY EARLY?
The study doctor may end your participation in the study early (even if you want to continue) if: you are not able to attend the study visits or to complete the required study procedures or comply with required study procedures; or the study is cancelled by your site's Institutional Review Board (IRB)/Institutional Ethics Committee (IEC), or a government authority; or the Sponsor decides to stop the trial for any reason.

DO I HAVE OPTIONS OTHER THAN JOINING THIS STUDY?
You can choose not to join this study at all.

IS THERE ANY COST FOR ME TO BE IN THIS STUDY?
You do not have to pay anything for the vaginal ring, for the study-related clinic visits, physical and pelvic exams, or laboratory tests. These are all free of charge.

DO I RECEIVE ANYTHING FOR MY TIME & TRAVEL?
You will be given **X amount** for your time and travel expenses.

WHO WILL SEE MY PERSONAL & MEDICAL INFORMATION?
We will do everything we can to protect your privacy. You will be assigned a code number which will be used on all information collected about you (instead of your name) on this study.

Subject ID Number

Subject Initials

A master list with your name and code will be kept under lock and key at the clinic where you are enrolled. Your name will never be used in any publication or presentation about this study. However people or groups that may review your records include your country's regulatory agency and other national regulatory agencies, e.g., the U.S. Food and Drug Administration (FDA), Ethics Committees, study monitors, the manufacturing company or the Sponsor of the study (IPM).

WHAT HAPPENS IF I AM INJURED?

If you become ill or injured as a result of using the vaginal ring or for any other procedure that is part of the study, you will receive medical treatment free of charge. The study staff also will tell you where you can get additional treatment, if needed. We will pay your reasonable medical costs for treatment of any illness or injury that is associated with your joining in the study.
If you suffer from an injury that is caused directly by your participation in the study (while inserting the vaginal ring or having any of the study procedures, or having any other medical procedure because of a side effect of using the vaginal ring), you may be able to receive some compensation. The compensation will only be given if the injury is serious and the effects of it will last for a long time.

WHAT IF I HAVE QUESTIONS OR PROBLEMS?

If you have any questions, you can contact a member of the clinic staff or Dr.______________ Tel___________________. If at any time you have any questions regarding your rights as a subject in a research study, you may contact___________________.

SIGNATURE TO JOIN THE IPM 011 RING STUDY:

I have read this consent form (or had it explained to me), and all of my questions have been answered to my satisfaction. I know that I can refuse to join this study, or if I agree to join I can drop out of the study at any time without losing any benefits or services to which I am entitled. After signing below, I will receive a copy of this consent form. My signature (or thumbprint or mark) below confirms that I freely agree to **JOIN** this study.

___________________________________	___________________________________
Volunteer's Name (print)	Volunteer's Signature/Thumbprint/Mark & Date
___________________________________	___________________________________
Name of Staff Conducting Consent Session (print)	Staff Signature & Date
___________________________________	___________________________________
Investigator or Co-investigator (print)	Investigator or Co-investigator Signature & Date
___________________________________	_____________________________
Witness' Name (print) (AS APPRO PRIATE)	Witness's Signature & Date

Subject ID Number

Subject Initials

APPENDIX I: SAMPLE CONSENT FORM TEMPLATE FEMALE FOCUS GROUP

A SAFETY AND ACCEPTABILITY STUDY OF A VAGINAL RING MICROBICIDE DELIVERY METHOD FOR THE PREVENTION OF HIV INFECTION IN WOMEN

You are being invited to join in a female focus group as part of the research study find out women's and men's opinions about a vaginal ring. Vaginal rings have previously been used to deliver medicines to prevent pregnancy and to treat menopause. Research is being conducted to see if the ring can be used to deliver a vaginal microbicide to prevent HIV infection in women. Vaginal microbicides are medicines being developed to prevent HIV infection. In order to join, you must have completed the Vaginal Ring regimen in the main study IPM 011, a safety and acceptability study of a vaginal ring. The ring used in the IPM 011 study contained no medicine at all. This consent form will give you information to help you decide if you would like to join in the female focus group part of the IPM 011 study. The study is being conducted by ***insert clinic name*** and is sponsored by the International Partnership for Microbicides, U.S.

Before you decide if you want to join this study or not, we want to explain the study, its risks, its potential benefits, and what you will be asked to do. You may ask questions as we discuss the study, so that you understand what the study is about. It is important you know the following:

- Your participation is entirely voluntary.
- You may decide not to take part or to withdraw from the study at any time without losing the benefits of your routine medical care.
- If you take part in the focus group discussion, what you discuss during the group discussion will be heard by other focus group participants and will therefore not be confidential. However, you and other focus group participants will be asked to keep everything discussed during the focus group confidential from others who did not participate in the focus group discussion.
- If you take part in the focus group discussion, the group discussion will be taped and notes taken to help analyze the findings from the discussion.

If you agree to join this study, we will ask you to sign and date this consent form. We will give you a copy of this form to keep.

<u>WHAT IS THE STUDY FOR?</u>

This study is being done to find out women's and men's opinions about a vaginal ring intended to be used as a vaginal microbicide delivery method in the future for the prevention of HIV infection in women.

Subject ID Number

Subject Initials

WHAT WILL BE DISCUSSED IN THIS FOCUS GROUP?

In the group discussion, you will be asked to discuss your experiences and opinions regarding the use of the vaginal ring, including the following topics:

- Your experiences when using the ring
- Your partners' experiences when you used the ring
- Your experience of participating in the study
- Your interest in using the ring again
- Anything that bothered you about the ring
- Any suggestions for making use of the ring more acceptable to you

HOW MANY PEOPLE WILL TAKE PART IN THE STUDY?

About 20 women who have completed the Vaginal Ring regimen in IPM 011 at the clinic will join the study and take part in the focus group. The group will be composed of women only.

HOW LONG WILL I BE IN THE STUDY?

Your participation will be a one time visit that will last about 1 ½ to 2 hours.

WHAT DO I HAVE TO DO IF I AM IN THE STUDY?

If you are interested in participating in this part of the IPM 011 study, you will need to sign this consent form. If you are in the focus group, you will come to the clinic and participate in the group with other women who have completed the Vaginal Ring regimen in IPM 011. There will be a moderator (a person in charge of asking questions and leading the group) who will ask questions to find out the opinions of the women and male partner's experience with the vaginal ring. The discussions will be taped, notes will be taken, and a report of the taped discussion will be written at a later time.

WHAT ARE THE RISKS OF THE STUDY?

You may become embarrassed, worried, or anxious when discussing sexual behaviors and HIV. Your participation will take place in a private setting and we will do everything we can to protect your privacy.

ARE THERE BENEFITS TO TAKING PART IN THIS STUDY?

This study may be of no direct benefit to you. However, you or others may benefit in the future from information learned in this study to prevent HIV infection.

WHAT ARE MY OTHER OPTIONS?

Your other options are to not participate in the study.

Subject ID Number **Subject Initials**

HOW DO I FIND OUT ABOUT NEW FINDINGS ABOUT THE STUDY?

You will be told of any new information learned during the course of the study that might cause you to change your mind about staying in the study. At the end of the study, you will be told when the study results may be available and how to learn about them.

WHAT ABOUT CONFIDENTIALITY?

We will do everything we can to protect your privacy. However, absolute confidentiality cannot be guaranteed. Your personal information may be disclosed if required by law. Any publication of this study will not use your name or identify you personally.

People who may review your records include:

- Applicable Country Medical Control Council
- U.S. Food and Drug Administration (FDA);
- European Medicines Agency (EMEA);
- Institutional Review Boards/Ethics Committees;
- Regulatory authorities;
- Study staff
- Study monitors;
- Sponsor of the study

WHAT HAPPENS IF I AM INJURED?

If you become ill or injured as a result of joining in the female focus group or as a result of using the vaginal ring or for any other procedure that is part of the study, the study staff will give you immediate necessary treatment, free of charge. The study staff also will tell you where you can get additional treatment, if needed. We will pay your reasonable medical expenses for treatment of any such illness or injury.

If you suffer from an injury that is caused directly by you joining in the study (while inserting the vaginal ring or having any of the study procedures, or having any other medical procedure because of a side effect of using the vaginal ring), you may be able to receive some compensation. The compensation will only be given if the injury is serious and the effects of it will last for a long time.

WHAT ARE THE COSTS OF THE STUDY?

There is no cost to you for taking part in the study. You will be reimbursed **X amount** for your time and travel.

Subject ID Number **Subject Initials**

WHAT ARE MY RIGHTS AS A RESEARCH VOLUNTEER?
Taking part in this study is completely voluntary. You may choose not to take part in this study or leave this study at any time.

After the study has been completed, if you want the results of this study, let the study staff know, and when they are publicly reported, you will be told where to find information from this or other studies that may affect your health, welfare, or willingness to stay in this study.

WHAT DO I DO IF I HAVE QUESTIONS OR PROBLEMS?
For questions about this study or a research-related injury, contact:
- [insert name of the investigator or other study staff]
- [inset telephone number of above]

For questions about your rights as a research volunteer, contact:
- [insert name or title of person on the Institutional Review Board (IRB) or other organization appropriate for the site]
- [insert telephone number of above]

SIGNATURE TO JOIN THE IPM 011 FEMALE FOCUS GROUP

I have read this consent form (or had it explained to me), and all of my questions have been answered to my satisfaction. I know that I can refuse to join in this study, or if I agree to join I can drop out of the study at any time without losing any benefits or services to which I am entitled. After signing below, I will receive a copy of this consent form. My signature (or thumbprint or mark) below confirms that I freely agree to **JOIN** this study.

______________________________	______________________________
Volunteer's Name (print)	Volunteer's Signature/Thumbprint/Mark & Date
______________________________	______________________________
Name of Staff Conducting Consent Session (print)	Staff Signature & Date
______________________________	______________________________
Investigator or Co-investigator (print)	Investigator or Co-investigator Signature & Date
______________________________	______________________________
Witness' Name (print) (AS APPRO PRIATE)	Witness's Signature & Date

APPENDIX J: SAMPLE CONSENT FORM TEMPLATE MALE INDIVIDUAL INTERVIEW

A SAFETY AND ACCEPTABILITY STUDY OF A VAGINAL RING MICROBICIDE DELIVERY METHOD FOR THE PREVENTION OF HIV INFECTION IN WOMEN

You are being invited to participate in an individual interview as part of a research study find out women's and men's opinions about a vaginal ring. Vaginal rings have previously been used to deliver medicines to prevent pregnancy and to treat menopause. Research is being conducted to see if the ring can be used to deliver a vaginal microbicide to prevent HIV infection in women. Vaginal microbicides are medicines being developed to prevent HIV infection. In order to join, you must be a sexual partner of a woman who completed the Vaginal Ring regimen in the main study IPM 011, a safety and acceptability study of a vaginal ring. This consent form will give you information to help you decide if you would like to participate in an individual interview which is part of the IPM 011 study. The study is being conducted by ***insert clinic name*** and is sponsored by the International Partnership for Microbicides, U.S.
Before you decide if you want to join this study or not, we want to explain the study, its risks, its potential benefits, and what you will be asked to do. You may ask questions as we discuss the study, so that you understand what the study is about. It is important you know the following:

- Your participation is entirely voluntary.
- You may decide not to take part or to withdraw from the study at any time without losing the benefits of your routine medical care.
- If you take part in the individual interview, the interview will be taped and notes taken to help analyze the findings from the discussion.

If you agree to join this study, we will ask you to sign and date this consent form. We will give you a copy of this form to keep.

WHAT IS THE STUDY FOR?
This study is being done to find out women's and men's opinions about a vaginal ring intended to be used as a vaginal microbicide delivery method in the future for the prevention of HIV infection in women.

WHAT WILL BE DISCUSSED IN THIS INDIVIDUAL INTERVIEW?
In the individual interview, you will be asked to discuss your experiences and opinions regarding the use of the vaginal ring, including the following topics:

- Your experiences when your woman partner used the ring
- Your experiences when your woman partner participated in this study
- Your interest in having your woman partner use the ring again
- Anything that bothered you about your woman partner's use of the ring
- Any suggestions for making your partner's use of the ring more acceptable to you

HOW MANY PEOPLE WILL TAKE PART IN THE STUDY?
About 20 men who are sexual partners of women who have completed the Vaginal Ring regimen in IPM 011 at the clinic will join the study and have an individual interview.

HOW LONG WILL I BE IN THE STUDY?
Your participation will be a one time visit that will last about 1 to 1 ½ hours.

WHAT DO I HAVE TO DO IF I AM IN THE STUDY?
If you are interested in participating in this part of the IPM 011 study, you will need to sign this consent form. If you agree to participate in the individual interview, you will come to the clinic and be interviewed to find out your opinions and experience with your sexual partner's use of the vaginal ring. The interview will be taped, notes will be taken, and a report of the taped interview will be written at a later time.

WHAT ARE THE RISKS OF THE STUDY?
You may become embarrassed, worried, or anxious when discussing sexual behaviors and HIV. Your participation will take place in a private setting and we will do everything we can to protect your privacy.

ARE THERE BENEFITS TO TAKING PART IN THIS STUDY?
This study may be of no direct benefit to you. However, you or others may benefit in the future from information learned in this study to prevent HIV infection.

WHAT ARE MY OTHER OPTIONS?

Your other options are to not participate in the study.

HOW DO I FIND OUT ABOUT NEW FINDINGS ABOUT THE STUDY?
You will be told of any new information learned during the course of the study that might cause you to change your mind about staying in the study. At the end of the study, you will be told when the study results may be available and how to learn about them.

WHAT ABOUT CONFIDENTIALITY?
We will do everything we can to protect your privacy. However, absolute confidentiality cannot be guaranteed. Your personal information may be disclosed if required by law. Any publication of this study will not use your name or identify you personally.

People who may review your records include:
- Applicable Country Medical Control Council
- U.S. Food and Drug Administration (FDA);
- European Medicines Agency (EMEA);
- Institutional Review Boards/Ethics Committees;
- Regulatory authorities;
- Study staff

- Study monitors;
- Sponsor of the study

WHAT HAPPENS IF I AM INJURED?
If you become ill or injured as a result of participating in the individual interview, the study staff will give you immediate necessary treatment, free of charge. The study staff also will tell you where you can get additional treatment, if needed. We will pay your reasonable medical expenses for treatment of any such illness or injury.

We will pay your partner compensation for illness or injury resulting from the use of the products provided in the study, medical treatment for an adverse reaction to those products, or any other procedure that is part of the study. If your partner suffers from an injury that is caused directly by her joining in the study (while inserting the vaginal ring or having any of the study procedures, or having any other medical procedure because of a side effect of using the vaginal ring), she may be able to receive some compensation. The compensation will only be given if the injury is serious and the effects of it will last for a long time.

WHAT ARE THE COSTS OF THE STUDY?
There is no cost to you for taking part in the study. You will be reimbursed **X amount** for your time and travel.

WHAT ARE MY RIGHTS AS A RESEARCH VOLUNTEER?
Taking part in this study is completely voluntary. You may choose not to take part in this study or leave this study at any time.

After the study has been completed, if you want the results of this study, let the study staff know, and when they are publicly reported, you will be told where to find information from this or other studies that may affect your health, welfare, or willingness to stay in this study.

WHAT DO I DO IF I HAVE QUESTIONS OR PROBLEMS?
For questions about this study or a research-related injury, contact:
- [insert name of the investigator or other study staff]
- [inset telephone number of above]

For questions about your rights as a research volunteer, contact:
- [insert name or title of person on the Institutional Review Board (IRB) or other organization appropriate for the site]
- [insert telephone number of above]

SIGNATURE TO JOIN THE IPM 011 MALE INDIVIDUAL INTERVIEW

I have read this consent form (or had it explained to me), and all of my questions have been answered to my satisfaction. I know that I can refuse to join in this study, or if I agree to join I can drop out of the study at any time without losing any benefits or services to which I am entitled. After signing below, I will receive a copy of this consent form. My signature (or thumbprint or mark) below confirms that I freely agree to **JOIN** this study.

Volunteer's Name (print)	Volunteer's Signature/Thumbprint/Mark & Date
Name of Staff Conducting Consent Session (print)	Staff Signature & Date
Investigator or Co-investigator (print)	Investigator or Co-investigator Signature & Date
Witness' Name (print) (AS APPRO PRIATE)	Witness's Signature & Date
