# Supplementary material for: Safety of a silicone elastomer vaginal ring as potential microbicide delivery method in African women: A Phase 1 randomized trial
Source: PLoS One. 2018 May 29;13(5):e0196904. doi: 10.1371/journal.pone.0196904 (PMC5973569; doi:10.1371/journal.pone.0196904)
Supplement: S1 Table — (PDF) [file pone.0196904.s003.pdf]

S1 Table. Data points for Baseline Demographics.

Demographic or Baseline Characteristic	Site 2 (N=50) n (%)		Site 3 (N=50) n (%)		Site 4 (N=50) n (%)		Site 5 (N=20) n (%)		All Sites (N=170) n (%)	
	Group		Group		Group		Group		Group	
	A	B	A	B	A	B	A	B	A	B
Race/ethnicity										
Black	25(100%)	25(100%)	27(100%)	23(100%)	22(81%)	19(83%)	9(100%)	10(91%)	83(94%)	77(94%)
Coloured	-	-	-	-	-	-	-	1(9%)	-	1(1%)
Indian	-	-	-	-	5(19%)	4(17%)	-	-	5(6%)	4(5%)
Age*	29(18-34)	29(23-34)	25(19-34)	25(19-35)	27(20-35)	27(22-34)	23(18-33)	26(18-32)	27(18-35)	27(18-35)
Marital status										
Not married	9(36%)	6(24%)	23(85%)	18(78%)	23(85%)	16(70%)	9(100%)	9(82%)	64(73%)	49(60%)
Married	14(56%)	18(72%)	3(11%)	3(13%)	4(15%)	7(30%)	-	2(18%)	21(24%)	30(37%)
Separated	-	-	1(4%)	-	-	-	-	-	1(1%)	-
Divorced	1(4%)	1(4%)	-	1(4%)	-	-	-	-	1(1%)	2(2%)
Widowed	1(4%)	-	-	1(4%)	-	-	-	-	1(1%)	1(1%)
Has main sex partner										
Yes	25(100%)	25(100%)	27(100%)	23(100%)	27(100%)	23(100%)	9(100%)	11(100%)	88(100%)	82(100%)
Number of sex partners in past 3 months										
1	25(100%)	24(96%)	26(96%)	23(100%)	27(100%)	22(96%)	8(89%)	11(100%)	86(98%)	80(98%)
2+	-	1(4%)	1(4%)	-	-	1(4%)	1(11%)	-	2(2%)	2(2%)

* Median values (and range) are reported for these variables.

** Among those reporting sex in the past 7 days.

Demographic or Baseline Characteristic	Site 2 (N=50) n (%)		Site 3 (N=50) n (%)		Site 4 (N=50) n (%)		Site 5 (N=20) n (%)		All Sites (N=170) n (%)	
	Group		Group		Group		Group		Group	
	A	B	A	B	A	B	A	B	A	B
Perceived risk of getting HIV compared to others										
High	5(20%)	6(24%)	2(7%)	4(17%)	-	-	2(22%)	1(9%)	9(10%)	11(14%)
About the same	6(24%)	5(20%)	3(11%)	4(17%)	1(4%)	2(9%)	3(33%)	-	13(15%)	11(14%)
Low	14(56%)	14(56%)	22(81%)	15(65%)	25(96%)	20(91%)	4(44%)	10(91%)	65(75%)	59(73%)
Has ever used male condom										
Yes	14(56%)	11(44%)	26(96%)	23(100%)	24(89%)	21(91%)	9(100%)	11(100%)	73(83%)	66(80%)
No	11(44%)	14(56%)	1(4%)	-	3(11%)	2(9%)	-	-	15(17%)	16(20%)
Number of sex acts with main partner in past 7 days*	1(0-3)	1(0-4)	3(0-7)	2(0-14)	2(0-8)	2(0-6)	2(0-4)	2(0-4)	2(0-8)	1(0-14)
Condom use in past 7 days with main partner**	**(N=37)**		**(N=41)**		**(N=45)**		**(N=18)**		**(N=141)**	
None	12(75%)	18(86%)	3(14%)	-	6(25%)	1(5%)	1(13%)	-	22(31%)	19(27%)
Some	-	-	-	1(5%)	3(13%)	3(14%)	1(13%)	-	4(6%)	4(6%)
Every time had sex	4(25%)	3(14%)	19(86%)	18(95%)	15(63%)	17(81%)	6(75%)	10(100%)	44(63%)	48(68%)

* Median values (and range) are reported for these variables.

** Among those reporting sex in the past 7 days.
