# Supplementary material for: Safety of a silicone elastomer vaginal ring as potential microbicide delivery method in African women: A Phase 1 randomized trial
Source: PLoS One. 2018 May 29;13(5):e0196904. doi: 10.1371/journal.pone.0196904 (PMC5973569; doi:10.1371/journal.pone.0196904)
Supplement: S2 Table — (PDF) [file pone.0196904.s004.pdf]

S2 Table. Prevalence of STIs and Bacterial Vaginosis – ITT Population.

		Observation					Intervention					
	Exams	Site 2 n(%)	Site 3 n(%)	Site 4 n(%)	Site 5 n(%)	Total n(%)	Site 2 n(%)	Site 3 n(%)	Site 4 n(%)	Site 5 n(%)	Total n(%)	P-value
Both Groups												
	HIV	0	0	1 (1)	0	1 (1)	0	0	2 (1)	0	2 (1)	
	Bacterial Vaginosis	4 (2)	8 (5)	10 (6)	6 (4)	28 (17)	0	7 (4)	11 (7)	2 (1)	20 (13)	0.178
	Trichomonas	3 (2)	6 (4)	3 (2)	0	12 (7)	0	4 (3)	5 (3)	0	9 (6)	0.170
	Gonorrhea	0	1 (1)	2 (1)	0	3 (2)	3 (2)	2 (1)	2 (1)	0	7 (4)	0.356
	Chlamydia	3 (2)	6 (4)	3 (2)	3 (2)	15 (9)	0	5 (3)	2 (1)	1 (1)	8 (5)	0.497
	Pregnancy	0	1 (1)	0	0	1 (1)	0	0	0	0	0	
Group A												
	HIV	0	0	1 (1)	0	1 (1)	0	0	1 (1)	0	1 (1)	
	Bacterial Vaginosis	0	2 (3)	0	1 (1)	3 (4)	0	6 (7)	11 (13)	2 (2)	19 (22)	0.165
	Trichomonas	1 (1)	0	1 (1)	0	2 (3)	0	4 (5)	5 (6)	0	9 (10)	0.067
	Gonorrhea	0	1 (1)	0	0	1 (1)	2 (2)	1 (1)	2 (2)	0	5 (6)	0.301
	Chlamydia	1 (1)	4 (5)	1 (1)	2 (3)	8 (10)	0	2 (2)	2 (2)	1 (1)	5 (6)	0.630
	Pregnancy	0	1 (1)	0	0	1 (1)	0	0	0	0	0	
Group B												
	HIV	0	0	0	0	0	0	0	1 (1)	0	1 (1)	
	Bacterial Vaginosis	4 (5)	6 (7)	10 (12)	5 (6)	25 (30)	0	1 (1)	0	0	1 (1)	0.420
	Trichomonas	2 (2)	6 (7)	2 (2)	0	10 (12)	0	0	0	0	0	
	Gonorrhea	0	0	2 (2)	0	2 (2)	1 (1)	1 (1)	0	0	2 (3)	0.135
	Chlamydia	2 (2)	2 (2)	2 (2)	1 (1)	7 (9)	0	3 (4)	0	0	3 (4)	0.232
	Pregnancy	0	0	0	0	0	0	0	0	0	0	

S2 Table. Prevalence of STIs and Bacterial Vaginosis – PP 1 Population.

		Observation						Intervention						
	Exams	Site 2 n(%)	Site 3 n(%)	Site 4 n(%)	Site 5 n(%)	Total n(%)		Site 2 n(%)	Site 3 n(%)	Site 4 n(%)	Site 5 n(%)	Total n(%)		P-value
Both Groups														
	HIV	0	0	1 (1)	0	1 (1)		0	0	2 (1)	0	2 (1)		
	Bacterial Vaginosis	4 (3)	7 (5)	8 (6)	4 (3)	23 (16)		0	7 (5)	11 (7)	2 (1)	20 (13)		0.175
	Trichomonas	3 (2)	5 (3)	3 (2)	0	11 (8)		0	3 (2)	5 (3)	0	8 (5)		0.164
	Gonorrhea	0	1 (1)	2 (1)	0	3 (2)		3 (2)	2 (1)	2 (1)	0	7 (5)		0.356
	Chlamydia	2 (1)	6 (4)	3 (2)	3 (2)	14 (10)		0	5 (3)	2 (1)	1 (1)	8 (5)		0.618
	Pregnancy	0	1 (1)	0	0	1 (1)		0	0	0	0	0		
Group A														
	HIV	0	0	1 (1)	0	1 (1)		0	0	1 (1)	0	1 (1)		
	Bacterial Vaginosis	0	2 (3)	0	1 (1)	3 (4)		0	6 (7)	11 (13)	2 (2)	19 (23)		0.165
	Trichomonas	1 (1)	0	1 (1)	0	2 (3)		0	3 (4)	5 (6)	0	8 (10)		0.091
	Gonorrhea	0	1 (1)	0	0	1 (1)		2 (2)	1 (1)	2 (2)	0	5 (6)		0.301
	Chlamydia	1 (1)	4 (5)	1 (1)	2 (3)	8 (11)		0	2 (2)	2 (2)	1 (1)	5 (6)		0.630
	Pregnancy	0	1 (1)	0	0	1 (1)		0	0	0	0	0		
Group B														
	HIV	0	0	0	0	0		0	0	1 (1)	0	1 (1)		
	Bacterial Vaginosis	4 (6)	5 (7)	8 (12)	3 (4)	20 (29)		0	1 (1)	0	0	1 (1)		0.453
	Trichomonas	2 (3)	5 (7)	2 (3)	0	9 (13)		0	0	0	0	0		
	Gonorrhea	0	0	2 (3)	0	2 (3)		1 (1)	1 (1)	0	0	2 (3)		0.135
	Chlamydia	1 (1)	2 (3)	2 (3)	1 (1)	6 (9)		0	3 (4)	0	0	3 (4)		0.308
	Pregnancy	0	0	0	0	0		0	0	0	0	0		

S2 Table. Prevalence of STIs and Bacterial Vaginosis – PP 1 Population.

		Observation						Intervention						
	Exams	Site 2 n(%)	Site 3 n(%)	Site 4 n(%)	Site 5 n(%)	Total n(%)		Site 2 n(%)	Site 3 n(%)	Site 4 n(%)	Site 5 n(%)	Total n(%)		P-value
Both Groups														
	HIV	0	0	0	0	0		0	0	2 (1)	0	2 (1)		
	Bacterial Vaginosis	4 (3)	7 (5)	8 (5)	4 (3)	23 (15)		0	5 (3)	11 (8)	2 (1)	18 (13)		0.176
	Trichomonas	3 (2)	5 (3)	3 (2)	0	11 (7)		0	2 (1)	5 (3)	0	7 (5)		0.129
	Gonorrhea	0	1 (1)	2 (1)	0	3 (2)		3 (2)	2 (1)	1 (1)	0	6 (4)		0.223
	Chlamydia	2 (1)	6 (4)	3 (2)	3 (2)	14 (9)		0	4 (3)	1 (1)	1 (1)	6 (4)		0.699
	Pregnancy	0	1 (1)	0	0	1 (1)		0	0	0	0	0		
Group A														
	HIV	0	0	1 (1)	0	1 (1)		0	0	1 (1)	0	1 (1)		
	Bacterial Vaginosis	0	2 (3)	0	1 (1)	3 (4)		0	6 (7)	11 (13)	2 (2)	19 (22)		0.165
	Trichomonas	1 (1)	0	1 (1)	0	2 (3)		0	4 (5)	5 (6)	0	9 (10)		0.067
	Gonorrhea	0	1 (1)	0	0	1 (1)		2 (2)	1 (1)	2 (2)	0	5 (6)		0.301
	Chlamydia	1 (1)	4 (5)	1 (1)	2 (3)	8 (10)		0	2 (2)	2 (2)	1 (1)	5 (6)		0.630
	Pregnancy	0	1 (1)	0	0	1 (1)		0	0	0	0	0		
Group B														
	HIV	0	0	0	0	0		0	0	1 (1)	0	1 (1)		
	Bacterial Vaginosis	4 (5)	6 (7)	10 (12)	5 (6)	25 (30)		0	1 (1)	0	0	1 (1)		0.420
	Trichomonas	2 (2)	6 (7)	2 (2)	0	10 (12)		0	0	0	0	0		
	Gonorrhea	0	0	2 (2)	0	2 (2)		1 (1)	1 (1)	0	0	2 (3)		0.135
	Chlamydia	2 (2)	2 (2)	2 (2)	1 (1)	7 (9)		0	3 (4)	0	0	3 (4)		0.232
	Pregnancy	0	0	0	0	0		0	0	0	0	0		
