# Supplementary material for: Safety of a silicone elastomer vaginal ring as potential microbicide delivery method in African women: A Phase 1 randomized trial
Source: PLoS One. 2018 May 29;13(5):e0196904. doi: 10.1371/journal.pone.0196904 (PMC5973569; doi:10.1371/journal.pone.0196904)
Supplement: S3 Table — (PDF) [file pone.0196904.s005.pdf]

S3 Table. Summary Table for Adverse Event Occurrence Rates by Research Center and per Treatment Phase

MedDRA Preferred Term	Reported Severity (DAIDS Grade)	Center 02		Center 03	
		Ring Intervention Phase N=48	Observation Phase N=49	Ring Intervention Phase N=48	Observation Phase N=49
Participant with Any Adverse Event		28 (58.3%)	27 (55.1%)	35 (72.9%)	40 (81.6%)
ABDOMINAL MASS	Grade 1	0 (0.0%)	0 (0.0%)	1 (2.1%)	0 (0.0%)
ABDOMINAL PAIN	Grade 1,2,3	0 (0.0%)	0 (0.0%)	0 (0.0%)	3 (6.1%)
ABDOMINAL PAIN LOWER	Grade 1,2	2 (4.2%)	2 (4.1%)	1 (2.1%)	1 (2.0%)
ABDOMINAL PAIN UPPER	Grade 2	0 (0.0%)	0 (0.0%)	0 (0.0%)	0 (0.0%)
ABDOMINAL TENDERNESS	Grade 1	0 (0.0%)	0 (0.0%)	0 (0.0%)	0 (0.0%)
ABSCESS LIMB	Grade 2	0 (0.0%)	0 (0.0%)	2 (4.2%)	0 (0.0%)
ADNEXA UTERI PAIN	Grade 1	0 (0.0%)	0 (0.0%)	1 (2.1%)	1 (2.0%)
ANXIETY	Grade 2	0 (0.0%)	0 (0.0%)	1 (2.1%)	0 (0.0%)
APPENDICITIS	Grade 3	1 (2.1%)	0 (0.0%)	0 (0.0%)	0 (0.0%)
ARTHRALGIA	Grade 2	0 (0.0%)	1 (2.0%)	0 (0.0%)	0 (0.0%)
ASCARIASIS	Grade 2	0 (0.0%)	1 (2.0%)	0 (0.0%)	0 (0.0%)
ASTHMA	Grade 1	0 (0.0%)	0 (0.0%)	0 (0.0%)	0 (0.0%)
BACK PAIN	Grade 1	0 (0.0%)	0 (0.0%)	1 (2.1%)	0 (0.0%)
BREAST PAIN	Grade 1	0 (0.0%)	0 (0.0%)	1 (2.1%)	0 (0.0%)
BRONCHIAL HYPERREACTIVITY	Grade 1	0 (0.0%)	0 (0.0%)	0 (0.0%)	1 (2.0%)
BRONCHITIS	Grade 2	5 (10.4%)	4 (8.2%)	0 (0.0%)	0 (0.0%)
CERVICAL DISCHARGE	Grade 1	0 (0.0%)	0 (0.0%)	0 (0.0%)	0 (0.0%)
CERVICITIS	Grade 1,2	0 (0.0%)	0 (0.0%)	0 (0.0%)	0 (0.0%)
CERVIX ERYTHEMA	Grade 1	0 (0.0%)	0 (0.0%)	0 (0.0%)	0 (0.0%)
CERVIX HAEMORRHAGE UTERINE	Grade 1	0 (0.0%)	0 (0.0%)	1 (2.1%)	0 (0.0%)
CHEST PAIN	Grade 1	0 (0.0%)	0 (0.0%)	0 (0.0%)	1 (2.0%)

Centre 02: Moshi, Tanzania; Centre 03: Johannesburg, South Africa; Centre 04: Durban, South Africa
Centre 05: DTHF, Cape Town, South Africa

MedDRA Preferred Term	Reported Severity (DAIDS Grade)	Centre 02 Ring Intervention Phase N=48	Centre 02 Observation Phase N=49	Center 02 Ring Intervention Phase N=48	Center 02 Observation Phase N=49
COITAL BLEEDING	Grade 1	0 (0.0%)	0 (0.0%)	1 (2.1%)	0 (0.0%)
CONSTIPATION	Grade 1	0 (0.0%)	0 (0.0%)	0 (0.0%)	1 (2.0%)
CONTUSION	Grade 2	0 (0.0%)	0 (0.0%)	1 (2.1%)	0 (0.0%)
CONVERSION DISORDER	Grade 2	0 (0.0%)	0 (0.0%)	0 (0.0%)	0 (0.0%)
COUGH	Grade 1,2	2 (4.2%)	0 (0.0%)	1 (2.1%)	0 (0.0%)
CYSTITIS	Grade 1,2,3	0 (0.0%)	0 (0.0%)	2 (4.2%)	2 (4.1%)
DEAFNESS UNILATERAL	Grade 2	0 (0.0%)	0 (0.0%)	1 (2.1%)	0 (0.0%)
DEPRESSION	Grade 2	0 (0.0%)	0 (0.0%)	1 (2.1%)	0 (0.0%)
DERMATITIS ALLERGIC	Grade 1,2	0 (0.0%)	0 (0.0%)	1 (2.1%)	1 (2.0%)
DIARRHOEA	Grade 1,2	3 (6.3%)	1 (2.0%)	0 (0.0%)	1 (2.0%)
DIZZINESS	Grade 1	1 (2.1%)	0 (0.0%)	2 (4.2%)	1 (2.0%)
DYSENTERY	Grade 2	1 (2.1%)	0 (0.0%)	0 (0.0%)	0 (0.0%)
DYSMENORRHOEA	Grade 1,2	0 (0.0%)	0 (0.0%)	5 (10.4%)	5 (10.2%)
DYSPAREUNIA	Grade 1	0 (0.0%)	0 (0.0%)	1 (2.1%)	0 (0.0%)
DYSURIA	Grade 1	0 (0.0%)	0 (0.0%)	0 (0.0%)	0 (0.0%)
EAR PAIN	Grade 1	0 (0.0%)	0 (0.0%)	0 (0.0%)	1 (2.0%)
EAR PRURITUS	Grade 2	0 (0.0%)	0 (0.0%)	0 (0.0%)	0 (0.0%)
ECZEMA	Grade 1,2	0 (0.0%)	0 (0.0%)	1 (2.1%)	0 (0.0%)
EPISTAXIS	Grade 1	0 (0.0%)	0 (0.0%)	2 (4.2%)	0 (0.0%)
FATIGUE	Grade 1,2	1 (2.1%)	0 (0.0%)	0 (0.0%)	1 (2.0%)
FIBROADENOMA OF BREAST	Grade 1	0 (0.0%)	0 (0.0%)	1 (2.1%)	0 (0.0%)
FOLLICULITIS	Grade 1	0 (0.0%)	0 (0.0%)	1 (2.1%)	2 (4.1%)
FOOT FRACTURE	Grade 2	0 (0.0%)	0 (0.0%)	0 (0.0%)	0 (0.0%)

Centre 02: Moshi, Tanzania; Centre 03: Johannesburg, South Africa; Centre 04: Durban, South Africa
Centre 05: DTHF, Cape Town, South Africa

MedDRA Preferred Term	Reported Severity (DAIDS Grade)	Center 02		Center 03	
		Ring Intervention Phase N=48	Observation Phase N=49	Ring Intervention Phase N=48	Observation Phase N=49
GASTRITIS	Grade 1,2	0 (0.0%)	1 (2.0%)	0 (0.0%)	0 (0.0%)
GASTROENTERITIS	Grade 2	0 (0.0%)	0 (0.0%)	1 (2.1%)	0 (0.0%)
GASTROENTERITIS SHIGELLA	Grade 2	0 (0.0%)	2 (4.1%)	0 (0.0%)	0 (0.0%)
GENITAL BURNING SENSATION	Grade 1	0 (0.0%)	0 (0.0%)	1 (2.1%)	0 (0.0%)
GENITAL DISCOMFORT	Grade 1	0 (0.0%)	0 (0.0%)	1 (2.1%)	0 (0.0%)
GENITAL EROSION	Grade 1	0 (0.0%)	0 (0.0%)	2 (4.2%)	1 (2.0%)
GENITAL ERYTHEMA	Grade 1	0 (0.0%)	0 (0.0%)	1 (2.1%)	0 (0.0%)
GENITAL HERPES	Grade 1,2	0 (0.0%)	0 (0.0%)	2 (4.2%)	3 (6.1%)
GENITAL PAIN	Grade 1	0 (0.0%)	0 (0.0%)	1 (2.1%)	0 (0.0%)
GINGIVAL ULCERATION	Grade 1	0 (0.0%)	0 (0.0%)	1 (2.1%)	0 (0.0%)
GONORRHOEA	Grade 1,2	1 (2.1%)	0 (0.0%)	1 (2.1%)	2 (4.1%)
GYNAECOLOGICAL CHLAMYDIA INFECTION	Grade 1,2	0 (0.0%)	1 (2.0%)	4 (8.3%)	5 (10.2%)
HAEMORRHOIDS	Grade 1,2	0 (0.0%)	0 (0.0%)	1 (2.1%)	1 (2.0%)
HEAD INJURY	Grade 2	0 (0.0%)	0 (0.0%)	0 (0.0%)	1 (2.0%)
HEADACHE	Grade 1,2	4 (8.3%)	1 (2.0%)	6 (12.5%)	9 (18.4%)
HEAT RASH	Grade 2	0 (0.0%)	0 (0.0%)	0 (0.0%)	0 (0.0%)
INFLUENZA LIKE ILLNESS	Grade 1,2	3 (6.3%)	3 (6.1%)	8 (16.7%)	11 (22.4%)
INJURY	Grade 2	0 (0.0%)	0 (0.0%)	1 (2.1%)	0 (0.0%)
JOINT SPRAIN	Grade 2	0 (0.0%)	1 (2.0%)	1 (2.1%)	0 (0.0%)
LACERATION	Grade 1	0 (0.0%)	0 (0.0%)	0 (0.0%)	1 (2.0%)
LARYNGITIS	Grade 2	4 (8.3%)	3 (6.1%)	0 (0.0%)	0 (0.0%)
LARYNGOTRACHEITIS	Grade 2	1 (2.1%)	0 (0.0%)	0 (0.0%)	0 (0.0%)
LOWER RESPIRATORY TRACT INFECTION	Grade 2	0 (0.0%)	0 (0.0%)	1 (2.1%)	0 (0.0%)

Centre 02: Moshi, Tanzania; Centre 03: Johannesburg, South Africa; Centre 04: Durban, South Africa
Centre 05: DTHF, Cape Town, South Africa

MedDRA Preferred Term	Reported Severity (DAIDS Grade)	Center 02		Center 03	
		Ring Intervention Phase N=48	Observation Phase N=49	Ring Intervention Phase N=48	Observation Phase N=49
LYMPHADENOPATHY	Grade 1	0 (0.0%)	0 (0.0%)	1 (2.1%)	0 (0.0%)
MALAISE	Grade 1	2 (4.2%)	0 (0.0%)	0 (0.0%)	0 (0.0%)
MALARIA	Grade 2	4 (8.3%)	2 (4.1%)	0 (0.0%)	0 (0.0%)
MENORRHAGIA	Grade 1,2	1 (2.1%)	1 (2.0%)	1 (2.1%)	2 (4.1%)
METRORRHAGIA	Grade 1,2	3 (6.3%)	5 (10.2%)	3 (6.3%)	3 (6.1%)
MOUTH ULCERATION	Grade 2	0 (0.0%)	0 (0.0%)	0 (0.0%)	0 (0.0%)
MUSCLE STRAIN	Grade 1	0 (0.0%)	0 (0.0%)	1 (2.1%)	1 (2.0%)
NAIL TINEA	Grade 2	0 (0.0%)	0 (0.0%)	0 (0.0%)	0 (0.0%)
NASOPHARYNGITIS	Grade 1	0 (0.0%)	0 (0.0%)	0 (0.0%)	1 (2.0%)
NAUSEA	Grade 1	0 (0.0%)	1 (2.0%)	0 (0.0%)	1 (2.0%)
NECK PAIN	Grade 2	2 (4.2%)	0 (0.0%)	0 (0.0%)	0 (0.0%)
PAIN	Grade 1	0 (0.0%)	0 (0.0%)	0 (0.0%)	0 (0.0%)
PALPITATIONS	Grade 1	1 (2.1%)	0 (0.0%)	0 (0.0%)	0 (0.0%)
PEPTIC ULCER	Grade 2	0 (0.0%)	0 (0.0%)	0 (0.0%)	0 (0.0%)
PERIORBITAL ABSCESS	Grade 1	0 (0.0%)	0 (0.0%)	0 (0.0%)	0 (0.0%)
PHARYNGITIS	Grade 1	0 (0.0%)	0 (0.0%)	1 (2.1%)	1 (2.0%)
PHARYNGOLARYNGEAL PAIN	Grade 1	0 (0.0%)	0 (0.0%)	0 (0.0%)	1 (2.0%)
PRURITUS GENITAL	Grade 1	2 (4.2%)	0 (0.0%)	1 (2.1%)	0 (0.0%)
PYREXIA	Grade 1	0 (0.0%)	1 (2.0%)	0 (0.0%)	0 (0.0%)
RASH	Grade 2	0 (0.0%)	1 (2.0%)	0 (0.0%)	0 (0.0%)
RASH GENERALISED	Grade 1	0 (0.0%)	0 (0.0%)	0 (0.0%)	0 (0.0%)
RASH PRURITIC	Grade 2	2 (4.2%)	0 (0.0%)	0 (0.0%)	0 (0.0%)
RASH PUSTULAR	Grade 1	0 (0.0%)	0 (0.0%)	0 (0.0%)	0 (0.0%)

Centre 02: Moshi, Tanzania; Centre 03: Johannesburg, South Africa; Centre 04: Durban, South Africa
Centre 05: DTHF, Cape Town, South Africa

MedDRA Preferred Term	Reported Severity (DAIDS Grade)	Center 02 Ring Intervention Phase N=48	Center 02 Observation Phase N=49	Center 03 Ring Intervention Phase N=48	Center 03 Observation Phase N=49
RASH VESICULAR	Grade 1	0 (0.0%)	0 (0.0%)	0 (0.0%)	1 (2.0%)
SINUS HEADACHE	Grade 1	0 (0.0%)	0 (0.0%)	0 (0.0%)	1 (2.0%)
SINUSITIS	Grade 1,2	1 (2.1%)	1 (2.0%)	2 (4.2%)	1 (2.0%)
SKIN LACERATION	Grade 2	1 (2.1%)	0 (0.0%)	0 (0.0%)	0 (0.0%)
SOFT TISSUE INFECTION	Grade 1	0 (0.0%)	0 (0.0%)	0 (0.0%)	0 (0.0%)
SOFT TISSUE INJURY	Grade 1	0 (0.0%)	0 (0.0%)	0 (0.0%)	0 (0.0%)
STRESS	Grade 2	0 (0.0%)	0 (0.0%)	1 (2.1%)	0 (0.0%)
SUBCUTANEOUS ABSCESS	Grade 2	0 (0.0%)	0 (0.0%)	0 (0.0%)	1 (2.0%)
TINEA INFECTION	Grade 2	0 (0.0%)	0 (0.0%)	0 (0.0%)	0 (0.0%)
TONSILLITIS	Grade 2	3 (6.3%)	2 (4.1%)	2 (4.2%)	0 (0.0%)
TOOTH INJURY	Grade 2	0 (0.0%)	0 (0.0%)	1 (2.1%)	0 (0.0%)
TOOTHACHE	Grade 1,2	0 (0.0%)	0 (0.0%)	2 (4.2%)	0 (0.0%)
UPPER RESPIRATORY TRACT INFECTION	Grade 1,2	0 (0.0%)	2 (4.1%)	0 (0.0%)	2 (4.1%)
URINARY TRACT INFECTION	Grade 1,2	3 (6.3%)	0 (0.0%)	1 (2.1%)	0 (0.0%)
UTERINE PAIN	Grade 1	0 (0.0%)	0 (0.0%)	1 (2.1%)	1 (2.0%)
UTERINE SPASM	Grade 1,2	0 (0.0%)	0 (0.0%)	0 (0.0%)	2 (4.1%)
VAGINAL CANDIDIASIS	Grade 1,2	0 (0.0%)	2 (4.1%)	6 (12.5%)	3 (6.1%)
VAGINAL DISCHARGE	Grade 1	1 (2.1%)	0 (0.0%)	2 (4.2%)	0 (0.0%)
VAGINAL ERYTHEMA	Grade 1	0 (0.0%)	0 (0.0%)	0 (0.0%)	1 (2.0%)
VAGINAL INFECTION	Grade 2	0 (0.0%)	0 (0.0%)	0 (0.0%)	0 (0.0%)
VAGINAL LACERATION	Grade 2	0 (0.0%)	0 (0.0%)	0 (0.0%)	0 (0.0%)
VAGINAL ODOUR	Grade 1	0 (0.0%)	0 (0.0%)	3 (6.3%)	1 (2.0%)
VAGINAL PAIN	Grade 1	0 (0.0%)	0 (0.0%)	0 (0.0%)	0 (0.0%)

Centre 02: Moshi, Tanzania; Centre 03: Johannesburg, South Africa; Centre 04: Durban, South Africa
Centre 05: DTHF, Cape Town, South Africa

MedDRA Preferred Term	Reported Severity (DAIDS Grade)	Center 02		Center 03	
		Ring Intervention Phase N=48	Observation Phase N=49	Ring Intervention Phase N=48	Observation Phase N=49
VAGINITIS BACTERIAL	Grade 1,2	0 (0.0%)	0 (0.0%)	7 (14.6%)	6 (12.2%)
VIRAL RHINITIS	Grade 1	0 (0.0%)	0 (0.0%)	0 (0.0%)	1 (2.0%)
VULVAL ERYTHEMA	Grade 1	1 (2.1%)	0 (0.0%)	0 (0.0%)	0 (0.0%)
VULVAR EROSION	Grade 1	0 (0.0%)	1 (2.0%)	0 (0.0%)	0 (0.0%)
VULVOVAGINAL DISCOMFORT	Grade 1	0 (0.0%)	0 (0.0%)	0 (0.0%)	0 (0.0%)
VULVOVAGINAL DRYNESS	Grade 1	0 (0.0%)	0 (0.0%)	0 (0.0%)	1 (2.0%)
VULVOVAGINAL PRURITUS	Grade 1	0 (0.0%)	0 (0.0%)	1 (2.1%)	2 (4.1%)
VULVOVAGINITIS TRICHOMONAL	Grade 1,2	0 (0.0%)	1 (2.0%)	0 (0.0%)	0 (0.0%)

Centre 02: Moshi, Tanzania; Centre 03: Johannesburg, South Africa; Centre 04: Durban, South Africa
Centre 05: DTHF, Cape Town, South Africa

MedDRA Preferred Term	Reported Severity (DAIDS Grade)	Center 02		Center 03	
		Ring Intervention Phase N=45	Observation Phase N=45	Ring Intervention Phase N=17	Observation Phase N=18
Participant with Any Adverse Event		24 (53.3%)	27 (60.0%)	13 (76.5%)	13 (72.2%)
ABDOMINAL MASS	Grade 1	0 (0.0%)	0 (0.0%)	0 (0.0%)	0 (0.0%)
ABDOMINAL PAIN	Grade 1,2,3	1 (2.2%)	0 (0.0%)	0 (0.0%)	0 (0.0%)
ABDOMINAL PAIN LOWER	Grade 1,2	1 (2.2%)	0 (0.0%)	1 (5.9%)	0 (0.0%)
ABDOMINAL PAIN UPPER	Grade 2	0 (0.0%)	0 (0.0%)	0 (0.0%)	0 (0.0%)
ABDOMINAL TENDERNESS	Grade 1	0 (0.0%)	0 (0.0%)	1 (5.9%)	0 (0.0%)
ABSCESS LIMB	Grade 2	0 (0.0%)	0 (0.0%)	0 (0.0%)	0 (0.0%)
ADNEXA UTERI PAIN	Grade 1	0 (0.0%)	0 (0.0%)	0 (0.0%)	0 (0.0%)
ANXIETY	Grade 2	0 (0.0%)	0 (0.0%)	0 (0.0%)	0 (0.0%)
APPENDICITIS	Grade 3	0 (0.0%)	0 (0.0%)	0 (0.0%)	0 (0.0%)
ARTHRALGIA	Grade 2	0 (0.0%)	0 (0.0%)	0 (0.0%)	0 (0.0%)
ASCARIASIS	Grade 2	0 (0.0%)	0 (0.0%)	0 (0.0%)	0 (0.0%)
ASTHMA	Grade 1	1 (2.2%)	1 (2.2%)	0 (0.0%)	0 (0.0%)
BACK PAIN	Grade 1	1 (2.2%)	1 (2.2%)	0 (0.0%)	0 (0.0%)
BREAST PAIN	Grade 1	0 (0.0%)	0 (0.0%)	0 (0.0%)	0 (0.0%)
BRONCHIAL HYPERREACTIVITY	Grade 1	0 (0.0%)	0 (0.0%)	0 (0.0%)	0 (0.0%)
BRONCHITIS	Grade 2	0 (0.0%)	0 (0.0%)	0 (0.0%)	0 (0.0%)
CERVICAL DISCHARGE	Grade 1	0 (0.0%)	0 (0.0%)	1 (5.9%)	0 (0.0%)
CERVICITIS	Grade 1,2	0 (0.0%)	0 (0.0%)	0 (0.0%)	1 (5.6%)
CERVIX ERYTHEMA	Grade 1	0 (0.0%)	0 (0.0%)	2 (11.8%)	1 (5.6%)
CERVIX HAEMORRHAGE UTERINE	Grade 1	0 (0.0%)	0 (0.0%)	0 (0.0%)	0 (0.0%)
CHEST PAIN	Grade 1	0 (0.0%)	0 (0.0%)	0 (0.0%)	0 (0.0%)

Centre 02: Moshi, Tanzania; Centre 03: Johannesburg, South Africa; Centre 04: Durban, South Africa
Centre 05: DTHF, Cape Town, South Africa

MedDRA Preferred Term	Reported Severity (DAIDS Grade)	Center 02 Ring Intervention Phase N=45	Center 02 Observation Phase N=45	Center 03 Ring Intervention Phase N=17	Center 03 Observation Phase N=18
COITAL BLEEDING	Grade 1	0 (0.0%)	0 (0.0%)	0 (0.0%)	0 (0.0%)
CONSTIPATION	Grade 1	0 (0.0%)	0 (0.0%)	0 (0.0%)	0 (0.0%)
CONTUSION	Grade 2	0 (0.0%)	0 (0.0%)	0 (0.0%)	0 (0.0%)
CONVERSION DISORDER	Grade 2	0 (0.0%)	0 (0.0%)	0 (0.0%)	0 (0.0%)
COUGH	Grade 1,2	1 (2.2%)	0 (0.0%)	0 (0.0%)	0 (0.0%)
CYSTITIS	Grade 1,2,3	0 (0.0%)	0 (0.0%)	0 (0.0%)	0 (0.0%)
DEAFNESS UNILATERAL	Grade 2	0 (0.0%)	0 (0.0%)	0 (0.0%)	0 (0.0%)
DEPRESSION	Grade 2	0 (0.0%)	0 (0.0%)	0 (0.0%)	0 (0.0%)
DERMATITIS ALLERGIC	Grade 1,2	0 (0.0%)	0 (0.0%)	0 (0.0%)	0 (0.0%)
DIARRHOEA	Grade 1,2	1 (2.2%)	0 (0.0%)	0 (0.0%)	0 (0.0%)
DIZZINESS	Grade 1	0 (0.0%)	0 (0.0%)	0 (0.0%)	0 (0.0%)
DYSENTERY	Grade 2	0 (0.0%)	0 (0.0%)	0 (0.0%)	0 (0.0%)
DYSMENORRHOEA	Grade 1,2	0 (0.0%)	0 (0.0%)	0 (0.0%)	0 (0.0%)
DYSPAREUNIA	Grade 1	0 (0.0%)	1 (2.2%)	0 (0.0%)	0 (0.0%)
DYSURIA	Grade 1	1 (2.2%)	0 (0.0%)	0 (0.0%)	0 (0.0%)
EAR PAIN	Grade 1	0 (0.0%)	0 (0.0%)	0 (0.0%)	0 (0.0%)
EAR PRURITUS	Grade 2	0 (0.0%)	0 (0.0%)	1 (5.9%)	0 (0.0%)
ECZEMA	Grade 1,2	0 (0.0%)	1 (2.2%)	0 (0.0%)	0 (0.0%)
EPISTAXIS	Grade 1	0 (0.0%)	0 (0.0%)	0 (0.0%)	0 (0.0%)
FATIGUE	Grade 1,2	0 (0.0%)	0 (0.0%)	0 (0.0%)	0 (0.0%)
FIBROADENOMA OF BREAST	Grade 1	0 (0.0%)	0 (0.0%)	0 (0.0%)	0 (0.0%)
FOLLICULITIS	Grade 1	0 (0.0%)	0 (0.0%)	0 (0.0%)	1 (5.6%)
FOOT FRACTURE	Grade 2	0 (0.0%)	0 (0.0%)	0 (0.0%)	0 (0.0%)

Centre 02: Moshi, Tanzania; Centre 03: Johannesburg, South Africa; Centre 04: Durban, South Africa
Centre 05: DTHF, Cape Town, South Africa

MedDRA Preferred Term	Reported Severity (DAIDS Grade)	Center 02		Center 03	
		Ring Intervention Phase N=45	Observation Phase N=45	Ring Intervention Phase N=17	Observation Phase N=18
GASTRITIS	Grade 1,2	0 (0.0%)	0 (0.0%)	1 (5.9%)	0 (0.0%)
GASTROENTERITIS	Grade 2	0 (0.0%)	0 (0.0%)	0 (0.0%)	0 (0.0%)
GASTROENTERITIS SHIGELLA	Grade 2	0 (0.0%)	0 (0.0%)	0 (0.0%)	0 (0.0%)
GENITAL BURNING SENSATION	Grade 1	0 (0.0%)	0 (0.0%)	0 (0.0%)	0 (0.0%)
GENITAL DISCOMFORT	Grade 1	0 (0.0%)	0 (0.0%)	0 (0.0%)	0 (0.0%)
GENITAL EROSION	Grade 1	0 (0.0%)	0 (0.0%)	0 (0.0%)	0 (0.0%)
GENITAL ERYTHEMA	Grade 1	0 (0.0%)	0 (0.0%)	0 (0.0%)	0 (0.0%)
GENITAL HERPES	Grade 1,2	0 (0.0%)	0 (0.0%)	0 (0.0%)	0 (0.0%)
GENITAL PAIN	Grade 1	0 (0.0%)	0 (0.0%)	0 (0.0%)	0 (0.0%)
GINGIVAL ULCERATION	Grade 1	0 (0.0%)	0 (0.0%)	0 (0.0%)	0 (0.0%)
GONORRHOEA	Grade 1,2	1 (2.2%)	1 (2.2%)	0 (0.0%)	0 (0.0%)
GYNAECOLOGICAL CHLAMYDIA INFECTION	Grade 1,2	2 (4.4%)	1 (2.2%)	0 (0.0%)	2 (11.1%)
HAEMORRHOIDS	Grade 1,2	0 (0.0%)	0 (0.0%)	0 (0.0%)	0 (0.0%)
HEAD INJURY	Grade 2	0 (0.0%)	0 (0.0%)	0 (0.0%)	0 (0.0%)
HEADACHE	Grade 1,2	3 (6.7%)	0 (0.0%)	0 (0.0%)	0 (0.0%)
HEAT RASH	Grade 2	0 (0.0%)	0 (0.0%)	0 (0.0%)	0 (0.0%)
INFLUENZA LIKE ILLNESS	Grade 1,2	0 (0.0%)	0 (0.0%)	1 (5.9%)	2 (11.1%)
INJURY	Grade 2	0 (0.0%)	0 (0.0%)	0 (0.0%)	0 (0.0%)
JOINT SPRAIN	Grade 2	0 (0.0%)	0 (0.0%)	0 (0.0%)	0 (0.0%)
LACERATION	Grade 1	0 (0.0%)	0 (0.0%)	0 (0.0%)	0 (0.0%)
LARYNGITIS	Grade 2	0 (0.0%)	0 (0.0%)	0 (0.0%)	0 (0.0%)
LARYNGOTRACHEITIS	Grade 2	0 (0.0%)	0 (0.0%)	0 (0.0%)	0 (0.0%)
LOWER RESPIRATORY TRACT INFECTION	Grade 2	0 (0.0%)	0 (0.0%)	0 (0.0%)	0 (0.0%)

Centre 02: Moshi, Tanzania; Centre 03: Johannesburg, South Africa; Centre 04: Durban, South Africa
Centre 05: DTHF, Cape Town, South Africa

MedDRA Preferred Term	Reported Severity (DAIDS Grade)	Center 02 Ring Intervention Phase N=45	Center 02 Observation Phase N=45	Center 03 Ring Intervention Phase N=17	Center 03 Observation Phase N=18
LYMPHADENOPATHY	Grade 1	0 (0.0%)	0 (0.0%)	0 (0.0%)	0 (0.0%)
MALAISE	Grade 1	0 (0.0%)	0 (0.0%)	0 (0.0%)	0 (0.0%)
MALARIA	Grade 2	0 (0.0%)	0 (0.0%)	0 (0.0%)	0 (0.0%)
MENORRHAGIA	Grade 1,2	0 (0.0%)	0 (0.0%)	0 (0.0%)	0 (0.0%)
METRORRHAGIA	Grade 1,2	3 (6.7%)	9 (20.0%)	0 (0.0%)	0 (0.0%)
MOUTH ULCERATION	Grade 2	0 (0.0%)	0 (0.0%)	0 (0.0%)	0 (0.0%)
MUSCLE STRAIN	Grade 1	0 (0.0%)	0 (0.0%)	0 (0.0%)	0 (0.0%)
NAIL TINEA	Grade 2	0 (0.0%)	0 (0.0%)	0 (0.0%)	0 (0.0%)
NASOPHARYNGITIS	Grade 1	0 (0.0%)	0 (0.0%)	0 (0.0%)	0 (0.0%)
NAUSEA	Grade 1	0 (0.0%)	0 (0.0%)	0 (0.0%)	0 (0.0%)
NECK PAIN	Grade 2	0 (0.0%)	0 (0.0%)	0 (0.0%)	0 (0.0%)
PAIN	Grade 1	1 (2.2%)	0 (0.0%)	0 (0.0%)	0 (0.0%)
PALPITATIONS	Grade 1	0 (0.0%)	0 (0.0%)	0 (0.0%)	0 (0.0%)
PEPTIC ULCER	Grade 2	0 (0.0%)	0 (0.0%)	0 (0.0%)	0 (0.0%)
PERIORBITAL ABSCESS	Grade 1	0 (0.0%)	1 (2.2%)	0 (0.0%)	0 (0.0%)
PHARYNGITIS	Grade 1	0 (0.0%)	0 (0.0%)	0 (0.0%)	0 (0.0%)
PHARYNGOLARYNGEAL PAIN	Grade 1	0 (0.0%)	0 (0.0%)	0 (0.0%)	0 (0.0%)
PRURITUS GENITAL	Grade 1	0 (0.0%)	0 (0.0%)	0 (0.0%)	0 (0.0%)
PYREXIA	Grade 1	0 (0.0%)	0 (0.0%)	0 (0.0%)	0 (0.0%)
RASH	Grade 2	0 (0.0%)	0 (0.0%)	0 (0.0%)	0 (0.0%)
RASH GENERALISED	Grade 1	1 (2.2%)	0 (0.0%)	0 (0.0%)	0 (0.0%)
RASH PRURITIC	Grade 2	0 (0.0%)	0 (0.0%)	0 (0.0%)	0 (0.0%)
RASH PUSTULAR	Grade 1	0 (0.0%)	0 (0.0%)	0 (0.0%)	1 (5.6%)

Centre 02: Moshi, Tanzania; Centre 03: Johannesburg, South Africa; Centre 04: Durban, South Africa
Centre 05: DTHF, Cape Town, South Africa

MedDRA Preferred Term	Reported Severity (DAIDS Grade)	Centre 04		Center 02	
		Ring Intervention Phase N=45	Observation Phase N=45	Ring Intervention Phase N=17	Observation Phase N=18
RASH VESICULAR	Grade 1	0 (0.0%)	0 (0.0%)	0 (0.0%)	0 (0.0%)
SINUS HEADACHE	Grade 1	0 (0.0%)	0 (0.0%)	0 (0.0%)	0 (0.0%)
SINUSITIS	Grade 1,2	0 (0.0%)	0 (0.0%)	0 (0.0%)	0 (0.0%)
SKIN LACERATION	Grade 2	0 (0.0%)	0 (0.0%)	0 (0.0%)	0 (0.0%)
SOFT TISSUE INFECTION	Grade 1	0 (0.0%)	0 (0.0%)	0 (0.0%)	1 (5.6%)
SOFT TISSUE INJURY	Grade 1	0 (0.0%)	1 (2.2%)	0 (0.0%)	0 (0.0%)
STRESS	Grade 2	0 (0.0%)	0 (0.0%)	0 (0.0%)	0 (0.0%)
SUBCUTANEOUS ABSCESS	Grade 2	0 (0.0%)	0 (0.0%)	1 (5.9%)	0 (0.0%)
TINEA INFECTION	Grade 2	0 (0.0%)	0 (0.0%)	0 (0.0%)	0 (0.0%)
TONSILLITIS	Grade 2	0 (0.0%)	0 (0.0%)	1 (5.9%)	0 (0.0%)
TOOTH INJURY	Grade 2	0 (0.0%)	0 (0.0%)	0 (0.0%)	0 (0.0%)
TOOTHACHE	Grade 1,2	0 (0.0%)	0 (0.0%)	0 (0.0%)	0 (0.0%)
UPPER RESPIRATORY TRACT INFECTION	Grade 1,2	4 (8.9%)	1 (2.2%)	1 (5.9%)	5 (27.8%)
URINARY TRACT INFECTION	Grade 1,2	3 (6.7%)	3 (6.7%)	0 (0.0%)	0 (0.0%)
UTERINE PAIN	Grade 1	0 (0.0%)	0 (0.0%)	0 (0.0%)	0 (0.0%)
UTERINE SPASM	Grade 1,2	0 (0.0%)	0 (0.0%)	0 (0.0%)	0 (0.0%)
VAGINAL CANDIDIASIS	Grade 1,2	8 (17.8%)	10 (22.2%)	4 (23.5%)	2 (11.1%)
VAGINAL DISCHARGE	Grade 1	2 (4.4%)	3 (6.7%)	2 (11.8%)	0 (0.0%)
VAGINAL ERYTHEMA	Grade 1	0 (0.0%)	0 (0.0%)	0 (0.0%)	0 (0.0%)
VAGINAL INFECTION	Grade 2	0 (0.0%)	0 (0.0%)	0 (0.0%)	0 (0.0%)
VAGINAL LACERATION	Grade 2	0 (0.0%)	0 (0.0%)	1 (5.9%)	0 (0.0%)
VAGINAL ODOUR	Grade 1	0 (0.0%)	0 (0.0%)	0 (0.0%)	0 (0.0%)
VAGINAL PAIN	Grade 1	1 (2.2%)	0 (0.0%)	0 (0.0%)	0 (0.0%)

Centre 02: Moshi, Tanzania; Centre 03: Johannesburg, South Africa; Centre 04: Durban, South Africa
Centre 05: DTHF, Cape Town, South Africa

MedDRA Preferred Term	Reported Severity (DAIDS Grade)	Center 02		Center 03	
		Ring Intervention Phase N=45	Observation Phase N=45	Ring Intervention Phase N=17	Observation Phase N=18
VAGINITIS BACTERIAL	Grade 1,2	6 (13.3%)	5 (11.1%)	2 (11.8%)	2 (11.1%)
VIRAL RHINITIS	Grade 1	0 (0.0%)	0 (0.0%)	0 (0.0%)	0 (0.0%)
VULVAL ERYTHEMA	Grade 1	0 (0.0%)	0 (0.0%)	0 (0.0%)	0 (0.0%)
VULVAR EROSION	Grade 1	0 (0.0%)	0 (0.0%)	0 (0.0%)	0 (0.0%)
VULVOVAGINAL DISCOMFORT	Grade 1	0 (0.0%)	0 (0.0%)	2 (11.8%)	1 (5.6%)
VULVOVAGINAL DRYNESS	Grade 1	0 (0.0%)	0 (0.0%)	0 (0.0%)	0 (0.0%)
VULVOVAGINAL PRURITUS	Grade 1	1 (2.2%)	0 (0.0%)	0 (0.0%)	0 (0.0%)
VULVOVAGINITIS TRICHOMONAL	Grade 1,2	1 (2.2%)	2 (4.4%)	0 (0.0%)	0 (0.0%)

Centre 02: Moshi, Tanzania; Centre 03: Johannesburg, South Africa; Centre 04: Durban, South Africa
Centre 05: DTHF, Cape Town, South Africa

MedDRA Preferred Term	Reported Severity (DAIDS Grade)	Overall	
		Ring Intervention Phase N=158	Observation Phase N=161
Participant with Any Adverse Event		100 (63.3%)	107 (66.5%)
ABDOMINAL MASS	Grade 1	1 (0.6%)	0 (0.0%)
ABDOMINAL PAIN	Grade 1,2,3	1 (0.6%)	3 (1.9%)
ABDOMINAL PAIN LOWER	Grade 1,2	5 (3.2%)	3 (1.9%)
ABDOMINAL PAIN UPPER	Grade 2	0 (0.0%)	0 (0.0%)
ABDOMINAL TENDERNESS	Grade 1	1 (0.6%)	0 (0.0%)
ABSCESS LIMB	Grade 2	2 (1.3%)	0 (0.0%)
ADNEXA UTERI PAIN	Grade 1	1 (0.6%)	1 (0.6%)
ANXIETY	Grade 2	1 (0.6%)	0 (0.0%)
APPENDICITIS	Grade 3	1 (0.6%)	0 (0.0%)
ARTHRALGIA	Grade 2	0 (0.0%)	1 (0.6%)
ASCARIASIS	Grade 2	0 (0.0%)	1 (0.6%)
ASTHMA	Grade 1	1 (0.6%)	1 (0.6%)
BACK PAIN	Grade 1	2 (1.3%)	1 (0.6%)
BREAST PAIN	Grade 1	1 (0.6%)	0 (0.0%)
BRONCHIAL HYPERREACTIVITY	Grade 1	0 (0.0%)	1 (0.6%)
BRONCHITIS	Grade 2	5 (3.2%)	4 (2.5%)
CERVICAL DISCHARGE	Grade 1	1 (0.6%)	0 (0.0%)
CERVICITIS	Grade 1,2	0 (0.0%)	1 (0.6%)
CERVIX ERYTHEMA	Grade 1	2 (1.3%)	1 (0.6%)
CERVIX HAEMORRHAGE UTERINE	Grade 1	1 (0.6%)	0 (0.0%)
CHEST PAIN	Grade 1	0 (0.0%)	1 (0.6%)

Centre 02: Moshi, Tanzania; Centre 03: Johannesburg, South Africa; Centre 04: Durban, South Africa
Centre 05: DTHF, Cape Town, South Africa

MedDRA Preferred Term	Reported Severity (DAIDS Grade)	Overall	
		Ring Intervention Phase N=158	Observation Phase N=161
COITAL BLEEDING	Grade 1	1 (0.6%)	0 (0.0%)
CONSTIPATION	Grade 1	0 (0.0%)	1 (0.6%)
CONTUSION	Grade 2	1 (0.6%)	0 (0.0%)
CONVERSION DISORDER	Grade 2	0 (0.0%)	0 (0.0%)
COUGH	Grade 1,2	4 (2.5%)	0 (0.0%)
CYSTITIS	Grade 1,2,3	2 (1.3%)	2 (1.2%)
DEAFNESS UNILATERAL	Grade 2	1 (0.6%)	0 (0.0%)
DEPRESSION	Grade 2	1 (0.6%)	0 (0.0%)
DERMATITIS ALLERGIC	Grade 1,2	1 (0.6%)	1 (0.6%)
DIARRHOEA	Grade 1,2	4 (2.5%)	2 (1.2%)
DIZZINESS	Grade 1	3 (1.9%)	1 (0.6%)
DYSENTERY	Grade 2	1 (0.6%)	0 (0.0%)
DYSMENORRHOEA	Grade 1,2	5 (3.2%)	5 (3.1%)
DYSPAREUNIA	Grade 1	1 (0.6%)	1 (0.6%)
DYSURIA	Grade 1	1 (0.6%)	0 (0.0%)
EAR PAIN	Grade 1	0 (0.0%)	1 (0.6%)
EAR PRURITUS	Grade 2	1 (0.6%)	0 (0.0%)
ECZEMA	Grade 1,2	1 (0.6%)	1 (0.6%)
EPISTAXIS	Grade 1	2 (1.3%)	0 (0.0%)
FATIGUE	Grade 1,2	1 (0.6%)	1 (0.6%)
FIBROADENOMA OF BREAST	Grade 1	1 (0.6%)	0 (0.0%)
FOLLICULITIS	Grade 1	1 (0.6%)	3 (1.9%)
FOOT FRACTURE	Grade 2	0 (0.0%)	0 (0.0%)

Centre 02: Moshi, Tanzania; Centre 03: Johannesburg, South Africa; Centre 04: Durban, South Africa
Centre 05: DTHF, Cape Town, South Africa

MedDRA Preferred Term	Reported Severity (DAIDS Grade)	Overall	
		Ring Intervention Phase N=158	Observation Phase N=161
GASTRITIS	Grade 1,2	1 (0.6%)	1 (0.6%)
GASTROENTERITIS	Grade 2	1 (0.6%)	0 (0.0%)
GASTROENTERITIS SHIGELLA	Grade 2	0 (0.0%)	2 (1.2%)
GENITAL BURNING SENSATION	Grade 1	1 (0.6%)	0 (0.0%)
GENITAL DISCOMFORT	Grade 1	1 (0.6%)	0 (0.0%)
GENITAL EROSION	Grade 1	2 (1.3%)	1 (0.6%)
GENITAL ERYTHEMA	Grade 1	1 (0.6%)	0 (0.0%)
GENITAL HERPES	Grade 1,2	2 (1.3%)	3 (1.9%)
GENITAL PAIN	Grade 1	1 (0.6%)	0 (0.0%)
GINGIVAL ULCERATION	Grade 1	1 (0.6%)	0 (0.0%)
GONORRHOEA	Grade 1,2	3 (1.9%)	3 (1.9%)
GYNAECOLOGICAL CHLAMYDIA INFECTION	Grade 1,2	6 (3.8%)	9 (5.6%)
HAEMORRHOIDS	Grade 1,2	1 (0.6%)	1 (0.6%)
HEAD INJURY	Grade 2	0 (0.0%)	1 (0.6%)
HEADACHE	Grade 1,2	13 (8.2%)	10 (6.2%)
HEAT RASH	Grade 2	0 (0.0%)	0 (0.0%)
INFLUENZA LIKE ILLNESS	Grade 1,2	12 (7.6%)	16 (9.9%)
INJURY	Grade 2	1 (0.6%)	0 (0.0%)
JOINT SPRAIN	Grade 2	1 (0.6%)	1 (0.6%)
LACERATION	Grade 1	0 (0.0%)	1 (0.6%)
LARYNGITIS	Grade 2	4 (2.5%)	3 (1.9%)
LARYNGOTRACHEITIS	Grade 2	1 (0.6%)	0 (0.0%)
LOWER RESPIRATORY TRACT INFECTION	Grade 2	1 (0.6%)	0 (0.0%)

Centre 02: Moshi, Tanzania; Centre 03: Johannesburg, South Africa; Centre 04: Durban, South Africa
Centre 05: DTHF, Cape Town, South Africa

MedDRA Preferred Term	Reported Severity (DAIDS Grade)	Overall	
		Ring Intervention Phase N=158	Observation Phase N=161
LYMPHADENOPATHY	Grade 1	1 (0.6%)	0 (0.0%)
MALAISE	Grade 1	2 (1.3%)	0 (0.0%)
MALARIA	Grade 2	4 (2.5%)	2 (1.2%)
MENORRHAGIA	Grade 1,2	2 (1.3%)	3 (1.9%)
METRORRHAGIA	Grade 1,2	9 (5.7%)	17 (10.6%)
MOUTH ULCERATION	Grade 2	0 (0.0%)	0 (0.0%)
MUSCLE STRAIN	Grade 1	1 (0.6%)	1 (0.6%)
NAIL TINEA	Grade 2	0 (0.0%)	0 (0.0%)
NASOPHARYNGITIS	Grade 1	0 (0.0%)	1 (0.6%)
NAUSEA	Grade 1	0 (0.0%)	2 (1.2%)
NECK PAIN	Grade 2	2 (1.3%)	0 (0.0%)
PAIN	Grade 1	1 (0.6%)	0 (0.0%)
PALPITATIONS	Grade 1	1 (0.6%)	0 (0.0%)
PEPTIC ULCER	Grade 2	0 (0.0%)	0 (0.0%)
PERIORBITAL ABSCESS	Grade 1	0 (0.0%)	1 (0.6%)
PHARYNGITIS	Grade 1	1 (0.6%)	1 (0.6%)
PHARYNGOLARYNGEAL PAIN	Grade 1	0 (0.0%)	1 (0.6%)
PRURITUS GENITAL	Grade 1	3 (1.9%)	0 (0.0%)
PYREXIA	Grade 1	0 (0.0%)	1 (0.6%)
RASH	Grade 2	0 (0.0%)	1 (0.6%)
RASH GENERALISED	Grade 1	1 (0.6%)	0 (0.0%)
RASH PRURITIC	Grade 2	2 (1.3%)	0 (0.0%)
RASH PUSTULAR	Grade 1	0 (0.0%)	1 (0.6%)

Centre 02: Moshi, Tanzania; Centre 03: Johannesburg, South Africa; Centre 04: Durban, South Africa
Centre 05: DTHF, Cape Town, South Africa

MedDRA Preferred Term	Reported Severity (DAIDS Grade)	Overall	
		Ring Intervention Phase N=158	Observation Phase N=161
RASH VESICULAR	Grade 1	0 (0.0%)	1 (0.6%)
SINUS HEADACHE	Grade 1	0 (0.0%)	1 (0.6%)
SINUSITIS	Grade 1,2	3 (1.9%)	2 (1.2%)
SKIN LACERATION	Grade 2	1 (0.6%)	0 (0.0%)
SOFT TISSUE INFECTION	Grade 1	0 (0.0%)	1 (0.6%)
SOFT TISSUE INJURY	Grade 1	0 (0.0%)	1 (0.6%)
STRESS	Grade 2	1 (0.6%)	0 (0.0%)
SUBCUTANEOUS ABSCESS	Grade 2	1 (0.6%)	1 (0.6%)
TINEA INFECTION	Grade 2	0 (0.0%)	0 (0.0%)
TONSILLITIS	Grade 2	6 (3.8%)	2 (1.2%)
TOOTH INJURY	Grade 2	1 (0.6%)	0 (0.0%)
TOOTHACHE	Grade 1,2	2 (1.3%)	0 (0.0%)
UPPER RESPIRATORY TRACT INFECTION	Grade 1,2	5 (3.2%)	10 (6.2%)
URINARY TRACT INFECTION	Grade 1,2	7 (4.4%)	3 (1.9%)
UTERINE PAIN	Grade 1	1 (0.6%)	1 (0.6%)
UTERINE SPASM	Grade 1,2	0 (0.0%)	2 (1.2%)
VAGINAL CANDIDIASIS	Grade 1,2	18 (11.4%)	17 (10.6%)
VAGINAL DISCHARGE	Grade 1	7 (4.4%)	3 (1.9%)
VAGINAL ERYTHEMA	Grade 1	0 (0.0%)	1 (0.6%)
VAGINAL INFECTION	Grade 2	0 (0.0%)	0 (0.0%)
VAGINAL LACERATION	Grade 2	1 (0.6%)	0 (0.0%)
VAGINAL ODOUR	Grade 1	3 (1.9%)	1 (0.6%)
VAGINAL PAIN	Grade 1	1 (0.6%)	0 (0.0%)

Centre 02: Moshi, Tanzania; Centre 03: Johannesburg, South Africa; Centre 04: Durban, South Africa
Centre 05: DTHF, Cape Town, South Africa

MedDRA Preferred Term	Reported Severity (DAIDS Grade)	Overall	
		Ring Intervention Phase N=158	Observation Phas N=161
VAGINITIS BACTERIAL	Grade 1,2	15 (9.5%)	13 (8.1%)
VIRAL RHINITIS	Grade 1	0 (0.0%)	1 (0.6%)
VULVAL ERYTHEMA	Grade 1	1 (0.6%)	0 (0.0%)
VULVAR EROSION	Grade 1	0 (0.0%)	1 (0.6%)
VULVOVAGINAL DISCOMFORT	Grade 1	2 (1.3%)	1 (0.6%)
VULVOVAGINAL DRYNESS	Grade 1	0 (0.0%)	1 (0.6%)
VULVOVAGINAL PRURITUS	Grade 1	2 (1.3%)	2 (1.2%)
VULVOVAGINITIS TRICHOMONAL	Grade 1,2	1 (0.6%)	3 (1.9%)

Centre 02: Moshi, Tanzania; Centre 03: Johannesburg, South Africa; Centre 04: Durban, South Africa
Centre 05: DTHF, Cape Town, South Africa
