# Supplementary material for: Safety of a silicone elastomer vaginal ring as potential microbicide delivery method in African women: A Phase 1 randomized trial
Source: PLoS One. 2018 May 29;13(5):e0196904. doi: 10.1371/journal.pone.0196904 (PMC5973569; doi:10.1371/journal.pone.0196904)
Supplement: S4 Table — (PDF) [file pone.0196904.s006.pdf]

**S4 Table. Summary for Adverse Event Occurrence Rates by Sequence Assignment**

| MedDRA Preferred Term | Reported Severity (DAIDS Grade) | Group A N=87 | Group B N=82 | Total N=169 |
|---|---|---|---|---|
| Participant with Any Adverse Event | | 73 (83.9%) | 65 (79.3%) | 138 (81.7%) |
| ABDOMINAL MASS | Grade 1 | 1 (1.1%) | 0 (0.0%) | 1 (0.6%) |
| ABDOMINAL PAIN | Grade 1,2,3 | 4 (4.6%) | 0 (0.0%) | 4 (2.4%) |
| ABDOMINAL PAIN LOWER | Grade 1,2 | 6 (6.9%) | 2 (2.4%) | 8 (4.7%) |
| ABDOMINAL PAIN UPPER | Grade 2 | 0 (0.0%) | 0 (0.0%) | 0 (0.0%) |
| ABDOMINAL TENDERNESS | Grade 1 | 1 (1.1%) | 0 (0.0%) | 1 (0.6%) |
| ABSCESS LIMB | Grade 2 | 2 (2.3%) | 0 (0.0%) | 2 (1.2%) |
| ADNEXA UTERI PAIN | Grade 1 | 0 (0.0%) | 2 (2.4%) | 2 (1.2%) |
| ANXIETY | Grade 2 | 1 (1.1%) | 0 (0.0%) | 1 (0.6%) |
| APPENDICITIS | Grade 3 | 1 (1.1%) | 0 (0.0%) | 1 (0.6%) |
| ARTHRALGIA | Grade 2 | 0 (0.0%) | 1 (1.2%) | 1 (0.6%) |
| ASCARIASIS | Grade 2 | 1 (1.1%) | 0 (0.0%) | 1 (0.6%) |
| ASTHMA | Grade 1 | 1 (1.1%) | 1 (1.2%) | 2 (1.2%) |
| BACK PAIN | Grade 1 | 2 (2.3%) | 1 (1.2%) | 3 (1.8%) |
| BREAST PAIN | Grade 1 | 1 (1.1%) | 0 (0.0%) | 1 (0.6%) |
| BRONCHIAL HYPERREACTIVITY | Grade 1 | 0 (0.0%) | 1 (1.2%) | 1 (0.6%) |
| BRONCHITIS | Grade 2 | 5 (5.7%) | 3 (3.7%) | 8 (4.7%) |
| CERVICAL DISCHARGE | Grade 1 | 0 (0.0%) | 1 (1.2%) | 1 (0.6%) |
| CERVICITIS | Grade 1,2 | 0 (0.0%) | 1 (1.2%) | 1 (0.6%) |
| CERVIX ERYTHEMA | Grade 1 | 2 (2.3%) | 1 (1.2%) | 3 (1.8%) |
| CERVIX HAEMORRHAGE UTERINE | Grade 1 | 1 (1.1%) | 0 (0.0%) | 1 (0.6%) |
| CHEST PAIN | Grade 1 | 1 (1.1%) | 0 (0.0%) | 1 (0.6%) |
| COITAL BLEEDING | Grade 1 | 1 (1.1%) | 0 (0.0%) | 1 (0.6%) |
| CONSTIPATION | Grade 1 | 0 (0.0%) | 1 (1.2%) | 1 (0.6%) |

Centre 02: Moshi, Tanzania; Centre 03: Johannesburg, South Africa; Centre 04: Durban, South Africa
Centre 05: DTHF, Cape Town, South Africa

| MedDRA Preferred Term | Reported Severity (DAIDS Grade) | Group A N=87 | Group B N=82 | Total N=169 |
|---|---|---|---|---|
| CONTUSION | Grade 2 | 1 (1.1%) | 0 (0.0%) | 1 (0.6%) |
| CONVERSION DISORDER | Grade 2 | 0 (0.0%) | 0 (0.0%) | 0 (0.0%) |
| COUGH | Grade 1,2 | 4 (4.6%) | 0 (0.0%) | 4 (2.4%) |
| CYSTITIS | Grade 1,2,3 | 3 (3.4%) | 1 (1.2%) | 4 (2.4%) |
| DEAFNESS UNILATERAL | Grade 2 | 1 (1.1%) | 0 (0.0%) | 1 (0.6%) |
| DEPRESSION | Grade 2 | 1 (1.1%) | 0 (0.0%) | 1 (0.6%) |
| DERMATITIS ALLERGIC | Grade 1,2 | 1 (1.1%) | 1 (1.2%) | 2 (1.2%) |
| DIARRHOEA | Grade 1,2 | 2 (2.3%) | 4 (4.9%) | 6 (3.6%) |
| DIZZINESS | Grade 1 | 2 (2.3%) | 1 (1.2%) | 3 (1.8%) |
| DYSENTERY | Grade 2 | 1 (1.1%) | 0 (0.0%) | 1 (0.6%) |
| DYSMENORRHOEA | Grade 1,2 | 5 (5.7%) | 3 (3.7%) | 8 (4.7%) |
| DYSPAREUNIA | Grade 1 | 1 (1.1%) | 1 (1.2%) | 2 (1.2%) |
| DYSURIA | Grade 1 | 0 (0.0%) | 1 (1.2%) | 1 (0.6%) |
| EAR PAIN | Grade 1 | 1 (1.1%) | 0 (0.0%) | 1 (0.6%) |
| EAR PRURITUS | Grade 2 | 0 (0.0%) | 1 (1.2%) | 1 (0.6%) |
| ECZEMA | Grade 1,2 | 2 (2.3%) | 0 (0.0%) | 2 (1.2%) |
| EPISTAXIS | Grade 1 | 1 (1.1%) | 1 (1.2%) | 2 (1.2%) |
| FATIGUE | Grade 1,2 | 1 (1.1%) | 1 (1.2%) | 2 (1.2%) |
| FIBROADENOMA OF BREAST | Grade 1 | 1 (1.1%) | 0 (0.0%) | 1 (0.6%) |
| FOLLICULITIS | Grade 1 | 2 (2.3%) | 1 (1.2%) | 3 (1.8%) |
| FOOT FRACTURE | Grade 2 | 0 (0.0%) | 0 (0.0%) | 0 (0.0%) |
| GASTRITIS | Grade 1,2 | 0 (0.0%) | 2 (2.4%) | 2 (1.2%) |
| GASTROENTERITIS | Grade 2 | 1 (1.1%) | 0 (0.0%) | 1 (0.6%) |
| GASTROENTERITIS SHIGELLA | Grade 2 | 1 (1.1%) | 1 (1.2%) | 2 (1.2%) |

Centre 02: Moshi, Tanzania; Centre 03: Johannesburg, South Africa; Centre 04: Durban, South Africa
Centre 05: DTHF, Cape Town, South Africa

| MedDRA Preferred Term | Reported Severity (DAIDS Grade) | Group A N=87 | Group B N=82 | Total N=169 |
|---|---|---|---|---|
| GENITAL BURNING SENSATION | Grade 1 | 1 (1.1%) | 0 (0.0%) | 1 (0.6%) |
| GENITAL DISCOMFORT | Grade 1 | 0 (0.0%) | 1 (1.2%) | 1 (0.6%) |
| GENITAL EROSION | Grade 1 | 2 (2.3%) | 1 (1.2%) | 3 (1.8%) |
| GENITAL ERYTHEMA | Grade 1 | 0 (0.0%) | 1 (1.2%) | 1 (0.6%) |
| GENITAL HERPES | Grade 1,2 | 4 (4.6%) | 0 (0.0%) | 4 (2.4%) |
| GENITAL PAIN | Grade 1 | 0 (0.0%) | 1 (1.2%) | 1 (0.6%) |
| GINGIVAL ULCERATION | Grade 1 | 0 (0.0%) | 1 (1.2%) | 1 (0.6%) |
| GONORRHOEA | Grade 1,2 | 3 (3.4%) | 3 (3.7%) | 6 (3.6%) |
| GYNAECOLOGICAL CHLAMYDIA INFECTION | Grade 1,2 | 10 (11.5%) | 5 (6.1%) | 15 (8.9%) |
| HAEMORRHOIDS | Grade 1,2 | 0 (0.0%) | 1 (1.2%) | 1 (0.6%) |
| HEAD INJURY | Grade 2 | 1 (1.1%) | 0 (0.0%) | 1 (0.6%) |
| HEADACHE | Grade 1,2 | 15 (17.2%) | 6 (7.3%) | 21 (12.4%) |
| HEAT RASH | Grade 2 | 0 (0.0%) | 0 (0.0%) | 0 (0.0%) |
| INFLUENZA LIKE ILLNESS | Grade 1,2 | 11 (12.6%) | 15 (18.3%) | 26 (15.4%) |
| INJURY | Grade 2 | 1 (1.1%) | 0 (0.0%) | 1 (0.6%) |
| JOINT SPRAIN | Grade 2 | 1 (1.1%) | 1 (1.2%) | 2 (1.2%) |
| LACERATION | Grade 1 | 1 (1.1%) | 0 (0.0%) | 1 (0.6%) |
| LARYNGITIS | Grade 2 | 3 (3.4%) | 3 (3.7%) | 6 (3.6%) |
| LARYNGOTRACHEITIS | Grade 2 | 1 (1.1%) | 0 (0.0%) | 1 (0.6%) |
| LOWER RESPIRATORY TRACT INFECTION | Grade 2 | 0 (0.0%) | 1 (1.2%) | 1 (0.6%) |
| LYMPHADENOPATHY | Grade 1 | 0 (0.0%) | 1 (1.2%) | 1 (0.6%) |
| MALAISE | Grade 1 | 0 (0.0%) | 2 (2.4%) | 2 (1.2%) |
| MALARIA | Grade 2 | 2 (2.3%) | 3 (3.7%) | 5 (3.0%) |
| MENORRHAGIA | Grade 1,2 | 3 (3.4%) | 1 (1.2%) | 4 (2.4%) |
| METRORRHAGIA | Grade 1,2 | 8 (9.2%) | 15 (18.3%) | 23 (13.6%) |

Centre 02: Moshi, Tanzania; Centre 03: Johannesburg, South Africa; Centre 04: Durban, South Africa
Centre 05: DTHF, Cape Town, South Africa

| MedDRA Preferred Term | Reported Severity (DAIDS Grade) | Group A N=87 | Group B N=82 | Total N=169 |
|---|---|---|---|---|
| MOUTH ULCERATION | Grade 2 | 0 (0.0%) | 0 (0.0%) | 0 (0.0%) |
| MUSCLE STRAIN | Grade 1 | 1 (1.1%) | 1 (1.2%) | 2 (1.2%) |
| NAIL TINEA | Grade 2 | 0 (0.0%) | 0 (0.0%) | 0 (0.0%) |
| NASOPHARYNGITIS | Grade 1 | 0 (0.0%) | 1 (1.2%) | 1 (0.6%) |
| NAUSEA | Grade 1 | 2 (2.3%) | 0 (0.0%) | 2 (1.2%) |
| NECK PAIN | Grade 2 | 2 (2.3%) | 0 (0.0%) | 2 (1.2%) |
| PAIN | Grade 1 | 1 (1.1%) | 0 (0.0%) | 1 (0.6%) |
| PALPITATIONS | Grade 1 | 0 (0.0%) | 1 (1.2%) | 1 (0.6%) |
| PEPTIC ULCER | Grade 2 | 0 (0.0%) | 0 (0.0%) | 0 (0.0%) |
| PERIORBITAL ABSCESS | Grade 1 | 0 (0.0%) | 1 (1.2%) | 1 (0.6%) |
| PHARYNGITIS | Grade 1 | 1 (1.1%) | 1 (1.2%) | 2 (1.2%) |
| PHARYNGOLARYNGEAL PAIN | Grade 1 | 0 (0.0%) | 1 (1.2%) | 1 (0.6%) |
| PRURITUS GENITAL | Grade 1 | 0 (0.0%) | 3 (3.7%) | 3 (1.8%) |
| PYREXIA | Grade 1 | 0 (0.0%) | 1 (1.2%) | 1 (0.6%) |
| RASH | Grade 2 | 1 (1.1%) | 0 (0.0%) | 1 (0.6%) |
| RASH GENERALISED | Grade 1 | 1 (1.1%) | 0 (0.0%) | 1 (0.6%) |
| RASH PRURITIC | Grade 2 | 1 (1.1%) | 1 (1.2%) | 2 (1.2%) |
| RASH PUSTULAR | Grade 1 | 0 (0.0%) | 1 (1.2%) | 1 (0.6%) |
| RASH VESICULAR | Grade 1 | 1 (1.1%) | 0 (0.0%) | 1 (0.6%) |
| SINUS HEADACHE | Grade 1 | 1 (1.1%) | 0 (0.0%) | 1 (0.6%) |
| SINUSITIS | Grade 1,2 | 1 (1.1%) | 3 (3.7%) | 4 (2.4%) |
| SKIN LACERATION | Grade 2 | 1 (1.1%) | 0 (0.0%) | 1 (0.6%) |
| SOFT TISSUE INFECTION | Grade 1 | 0 (0.0%) | 1 (1.2%) | 1 (0.6%) |
| SOFT TISSUE INJURY | Grade 1 | 0 (0.0%) | 1 (1.2%) | 1 (0.6%) |
| STRESS | Grade 2 | 1 (1.1%) | 0 (0.0%) | 1 (0.6%) |

Centre 02: Moshi, Tanzania; Centre 03: Johannesburg, South Africa; Centre 04: Durban, South Africa
Centre 05: DTHF, Cape Town, South Africa

| MedDRA Preferred Term | Reported Severity (DAIDS Grade) | Group A N=87 | Group B N=82 | Total N=169 |
|---|---|---|---|---|
| SUBCUTANEOUS ABSCESS | Grade 2 | 1 (1.1%) | 1 (1.2%) | 2 (1.2%) |
| TINEA INFECTION | Grade 2 | 0 (0.0%) | 0 (0.0%) | 0 (0.0%) |
| TONSILLITIS | Grade 2 | 6 (6.9%) | 2 (2.4%) | 8 (4.7%) |
| TOOTH INJURY | Grade 2 | 1 (1.1%) | 0 (0.0%) | 1 (0.6%) |
| TOOTHACHE | Grade 1,2 | 2 (2.3%) | 0 (0.0%) | 2 (1.2%) |
| UPPER RESPIRATORY TRACT INFECTION | Grade 1,2 | 5 (5.7%) | 10 (12.2%) | 15 (8.9%) |
| URINARY TRACT INFECTION | Grade 1,2 | 5 (5.7%) | 3 (3.7%) | 8 (4.7%) |
| UTERINE PAIN | Grade 1 | 1 (1.1%) | 1 (1.2%) | 2 (1.2%) |
| UTERINE SPASM | Grade 1,2 | 2 (2.3%) | 0 (0.0%) | 2 (1.2%) |
| VAGINAL CANDIDIASIS | Grade 1,2 | 18 (20.7%) | 14 (17.1%) | 32 (18.9%) |
| VAGINAL DISCHARGE | Grade 1 | 4 (4.6%) | 6 (7.3%) | 10 (5.9%) |
| VAGINAL ERYTHEMA | Grade 1 | 1 (1.1%) | 0 (0.0%) | 1 (0.6%) |
| VAGINAL INFECTION | Grade 2 | 0 (0.0%) | 0 (0.0%) | 0 (0.0%) |
| VAGINAL LACERATION | Grade 2 | 0 (0.0%) | 1 (1.2%) | 1 (0.6%) |
| VAGINAL ODOUR | Grade 1 | 4 (4.6%) | 0 (0.0%) | 4 (2.4%) |
| VAGINAL PAIN | Grade 1 | 0 (0.0%) | 1 (1.2%) | 1 (0.6%) |
| VAGINITIS BACTERIAL | Grade 1,2 | 14 (16.1%) | 9 (11.0%) | 23 (13.6%) |
| VIRAL RHINITIS | Grade 1 | 0 (0.0%) | 1 (1.2%) | 1 (0.6%) |
| VULVAL ERYTHEMA | Grade 1 | 1 (1.1%) | 0 (0.0%) | 1 (0.6%) |
| VULVAR EROSION | Grade 1 | 0 (0.0%) | 1 (1.2%) | 1 (0.6%) |
| VULVOVAGINAL DISCOMFORT | Grade 1 | 0 (0.0%) | 3 (3.7%) | 3 (1.8%) |
| VULVOVAGINAL DRYNESS | Grade 1 | 0 (0.0%) | 1 (1.2%) | 1 (0.6%) |
| VULVOVAGINAL PRURITUS | Grade 1 | 2 (2.3%) | 2 (2.4%) | 4 (2.4%) |
| VULVOVAGINITIS TRICHOMONAL | Grade 1,2 | 4 (4.6%) | 0 (0.0%) | 4 (2.4%) |

Centre 02: Moshi, Tanzania; Centre 03: Johannesburg, South Africa; Centre 04: Durban, South Africa
Centre 05: DTHF, Cape Town, South Africa
