# Supplementary material for: Safety of a silicone elastomer vaginal ring as potential microbicide delivery method in African women: A Phase 1 randomized trial
Source: PLoS One. 2018 May 29;13(5):e0196904. doi: 10.1371/journal.pone.0196904 (PMC5973569; doi:10.1371/journal.pone.0196904)
Supplement: S5 Table — (PDF) [file pone.0196904.s007.pdf]

S5 Table. Summary of Adverse Event Occurrence Rates by Trial Phase.

MedDRA Preferred Term	Reported Severity (DAIDS Grade)	Ring Intervention Phase N=158	Observation Phase N=161
Participant with Any Adverse Event		100 (63.3%)	107 (66.5%)
ABDOMINAL MASS	Grade 1	1 (0.6%)	0 (0.0%)
ABDOMINAL PAIN	Grade 1,2,3	1 (0.6%)	3 (1.9%)
ABDOMINAL PAIN LOWER	Grade 1,2	5 (3.2%)	3 (1.9%)
ABDOMINAL PAIN UPPER	Grade 2	0 (0.0%)	0 (0.0%)
ABDOMINAL TENDERNESS	Grade 1	1 (0.6%)	0 (0.0%)
ABSCESS LIMB	Grade 2	2 (1.3%)	0 (0.0%)
ADNEXA UTERI PAIN	Grade 1	1 (0.6%)	1 (0.6%)
ANXIETY	Grade 2	1 (0.6%)	0 (0.0%)
APPENDICITIS	Grade 3	1 (0.6%)	0 (0.0%)
ARTHRALGIA	Grade 2	0 (0.0%)	1 (0.6%)
ASCARIASIS	Grade 2	0 (0.0%)	1 (0.6%)
ASTHMA	Grade 1	1 (0.6%)	1 (0.6%)
BACK PAIN	Grade 1	2 (1.3%)	1 (0.6%)
BREAST PAIN	Grade 1	1 (0.6%)	0 (0.0%)
BRONCHIAL HYPERREACTIVITY	Grade 1	0 (0.0%)	1 (0.6%)
BRONCHITIS	Grade 2	5 (3.2%)	4 (2.5%)
CERVICAL DISCHARGE	Grade 1	1 (0.6%)	0 (0.0%)
CERVICITIS	Grade 1,2	0 (0.0%)	1 (0.6%)
CERVIX ERYTHEMA	Grade 1	2 (1.3%)	1 (0.6%)
CERVIX HAEMORRHAGE UTERINE	Grade 1	1 (0.6%)	0 (0.0%)
CHEST PAIN	Grade 1	0 (0.0%)	1 (0.6%)

Centre 02: Moshi, Tanzania; Centre 03: Johannesburg, South Africa; Centre 04: Durban, South Africa
Centre 05: DTHF, Cape Town, South Africa

MedDRA Preferred Term	Reported Severity (DAIDS Grade)	Ring Intervention Phase N=158	Observation Phase N=161
COITAL BLEEDING	Grade 1	1 (0.6%)	0 (0.0%)
CONSTIPATION	Grade 1	0 (0.0%)	1 (0.6%)
CONTUSION	Grade 2	1 (0.6%)	0 (0.0%)
CONVERSION DISORDER	Grade 2	0 (0.0%)	0 (0.0%)
COUGH	Grade 1,2	4 (2.5%)	0 (0.0%)
CYSTITIS	Grade 1,2,3	2 (1.3%)	2 (1.2%)
DEAFNESS UNILATERAL	Grade 2	1 (0.6%)	0 (0.0%)
DEPRESSION	Grade 2	1 (0.6%)	0 (0.0%)
DERMATITIS ALLERGIC	Grade 1,2	1 (0.6%)	1 (0.6%)
DIARRHOEA	Grade 1,2	4 (2.5%)	2 (1.2%)
DIZZINESS	Grade 1	3 (1.9%)	1 (0.6%)
DYSENTERY	Grade 2	1 (0.6%)	0 (0.0%)
DYSMENORRHOEA	Grade 1,2	5 (3.2%)	5 (3.1%)
DYSPAREUNIA	Grade 1	1 (0.6%)	1 (0.6%)
DYSURIA	Grade 1	1 (0.6%)	0 (0.0%)
EAR PAIN	Grade 1	0 (0.0%)	1 (0.6%)
EAR PRURITUS	Grade 2	1 (0.6%)	0 (0.0%)
ECZEMA	Grade 1,2	1 (0.6%)	1 (0.6%)
EPISTAXIS	Grade 1	2 (1.3%)	0 (0.0%)
FATIGUE	Grade 1,2	1 (0.6%)	1 (0.6%)
FIBROADENOMA OF BREAST	Grade 1	1 (0.6%)	0 (0.0%)
FOLLICULITIS	Grade 1	1 (0.6%)	3 (1.9%)
FOOT FRACTURE	Grade 2	0 (0.0%)	0 (0.0%)

Centre 02: Moshi, Tanzania; Centre 03: Johannesburg, South Africa; Centre 04: Durban, South Africa
Centre 05: DTHF, Cape Town, South Africa

MedDRA Preferred Term	Reported Severity (DAIDS Grade)	Ring Intervention Phase N=158	Observation Phase N=161
GASTRITIS	Grade 1,2	1 (0.6%)	1 (0.6%)
GASTROENTERITIS	Grade 2	1 (0.6%)	0 (0.0%)
GASTROENTERITIS SHIGELLA	Grade 2	0 (0.0%)	2 (1.2%)
GENITAL BURNING SENSATION	Grade 1	1 (0.6%)	0 (0.0%)
GENITAL DISCOMFORT	Grade 1	1 (0.6%)	0 (0.0%)
GENITAL EROSION	Grade 1	2 (1.3%)	1 (0.6%)
GENITAL ERYTHEMA	Grade 1	1 (0.6%)	0 (0.0%)
GENITAL HERPES	Grade 1,2	2 (1.3%)	3 (1.9%)
GENITAL PAIN	Grade 1	1 (0.6%)	0 (0.0%)
GINGIVAL ULCERATION	Grade 1	1 (0.6%)	0 (0.0%)
GONORRHOEA	Grade 1,2	3 (1.9%)	3 (1.9%)
GYNAECOLOGICAL CHLAMYDIA INFECTION	Grade 1,2	6 (3.8%)	9 (5.6%)
HAEMORRHOIDS	Grade 1,2	1 (0.6%)	1 (0.6%)
HEAD INJURY	Grade 2	0 (0.0%)	1 (0.6%)
HEADACHE	Grade 1,2	13 (8.2%)	10 (6.2%)
HEAT RASH	Grade 2	0 (0.0%)	0 (0.0%)
INFLUENZA LIKE ILLNESS	Grade 1,2	12 (7.6%)	16 (9.9%)
INJURY	Grade 2	1 (0.6%)	0 (0.0%)
JOINT SPRAIN	Grade 2	1 (0.6%)	1 (0.6%)
LACERATION	Grade 1	0 (0.0%)	1 (0.6%)
LARYNGITIS	Grade 2	4 (2.5%)	3 (1.9%)
LARYNGOTRACHEITIS	Grade 2	1 (0.6%)	0 (0.0%)
LOWER RESPIRATORY TRACT INFECTION	Grade 2	1 (0.6%)	0 (0.0%)
LYMPHADENOPATHY	Grade 1	1 (0.6%)	0 (0.0%)

Centre 02: Moshi, Tanzania; Centre 03: Johannesburg, South Africa; Centre 04: Durban, South Africa
Centre 05: DTHF, Cape Town, South Africa

MedDRA Preferred Term	Reported Severity (DAIDS Grade)	Ring Intervention Phase N=158	Observation Phase N=161
MALAISE	Grade 1	2 (1.3%)	0 (0.0%)
MALARIA	Grade 2	4 (2.5%)	2 (1.2%)
MENORRHAGIA	Grade 1,2	2 (1.3%)	3 (1.9%)
METRORRHAGIA	Grade 1,2	9 (5.7%)	17 (10.6%)
MOUTH ULCERATION	Grade 2	0 (0.0%)	0 (0.0%)
MUSCLE STRAIN	Grade 1	1 (0.6%)	1 (0.6%)
NAIL TINEA	Grade 2	0 (0.0%)	0 (0.0%)
NASOPHARYNGITIS	Grade 1	0 (0.0%)	1 (0.6%)
NAUSEA	Grade 1	0 (0.0%)	2 (1.2%)
NECK PAIN	Grade 2	2 (1.3%)	0 (0.0%)
PAIN	Grade 1	1 (0.6%)	0 (0.0%)
PALPITATIONS	Grade 1	1 (0.6%)	0 (0.0%)
PEPTIC ULCER	Grade 2	0 (0.0%)	0 (0.0%)
PERIORBITAL ABSCESS	Grade 1	0 (0.0%)	1 (0.6%)
PHARYNGITIS	Grade 1	1 (0.6%)	1 (0.6%)
PHARYNGOLARYNGEAL PAIN	Grade 1	0 (0.0%)	1 (0.6%)
PRURITUS GENITAL	Grade 1	3 (1.9%)	0 (0.0%)
PYREXIA	Grade 1	0 (0.0%)	1 (0.6%)
RASH	Grade 2	0 (0.0%)	1 (0.6%)
RASH GENERALISED	Grade 1	1 (0.6%)	0 (0.0%)
RASH PRURITIC	Grade 2	2 (1.3%)	0 (0.0%)
RASH PUSTULAR	Grade 1	0 (0.0%)	1 (0.6%)
RASH VESICULAR	Grade 1	0 (0.0%)	1 (0.6%)
SINUS HEADACHE	Grade 1	0 (0.0%)	1 (0.6%)

Centre 02: Moshi, Tanzania; Centre 03: Johannesburg, South Africa; Centre 04: Durban, South Africa
Centre 05: DTHF, Cape Town, South Africa

MedDRA Preferred Term	Reported Severity (DAIDS Grade)	Ring Intervention Phase N=158	Observation Phase N=161
SINUSITIS	Grade 1,2	3 (1.9%)	2 (1.2%)
SKIN LACERATION	Grade 2	1 (0.6%)	0 (0.0%)
SOFT TISSUE INFECTION	Grade 1	0 (0.0%)	1 (0.6%)
SOFT TISSUE INJURY	Grade 1	0 (0.0%)	1 (0.6%)
STRESS	Grade 2	1 (0.6%)	0 (0.0%)
SUBCUTANEOUS ABSCESS	Grade 2	1 (0.6%)	1 (0.6%)
TINEA INFECTION	Grade 2	0 (0.0%)	0 (0.0%)
TONSILLITIS	Grade 2	6 (3.8%)	2 (1.2%)
TOOTH INJURY	Grade 2	1 (0.6%)	0 (0.0%)
TOOTHACHE	Grade 1,2	2 (1.3%)	0 (0.0%)
UPPER RESPIRATORY TRACT INFECTION	Grade 1,2	5 (3.2%)	10 (6.2%)
URINARY TRACT INFECTION	Grade 1,2	7 (4.4%)	3 (1.9%)
UTERINE PAIN	Grade 1	1 (0.6%)	1 (0.6%)
UTERINE SPASM	Grade 1,2	0 (0.0%)	2 (1.2%)
VAGINAL CANDIDIASIS	Grade 1,2	18 (11.4%)	17 (10.6%)
VAGINAL DISCHARGE	Grade 1	7 (4.4%)	3 (1.9%)
VAGINAL ERYTHEMA	Grade 1	0 (0.0%)	1 (0.6%)
VAGINAL INFECTION	Grade 2	0 (0.0%)	0 (0.0%)
VAGINAL LACERATION	Grade 2	1 (0.6%)	0 (0.0%)
VAGINAL ODOUR	Grade 1	3 (1.9%)	1 (0.6%)
VAGINAL PAIN	Grade 1	1 (0.6%)	0 (0.0%)
VAGINITIS BACTERIAL	Grade 1,2	15 (9.5%)	13 (8.1%)
VIRAL RHINITIS	Grade 1	0 (0.0%)	1 (0.6%)

Centre 02: Moshi, Tanzania; Centre 03: Johannesburg, South Africa; Centre 04: Durban, South Africa
Centre 05: DTHF, Cape Town, South Africa

MedDRA Preferred Term	Reported Severity (DAIDS Grade)	Ring Intervention Phase N=158	Observation Phase N=161
VULVAL ERYTHEMA	Grade 1	1 (0.6%)	0 (0.0%)
VULVAR EROSION	Grade 1	0 (0.0%)	1 (0.6%)
VULVOVAGINAL DISCOMFORT	Grade 1	2 (1.3%)	1 (0.6%)
VULVOVAGINAL DRYNESS	Grade 1	0 (0.0%)	1 (0.6%)
VULVOVAGINAL PRURITUS	Grade 1	2 (1.3%)	2 (1.2%)
VULVOVAGINITIS TRICHOMONAL	Grade 1,2	1 (0.6%)	3 (1.9%)

Centre 02: Moshi, Tanzania; Centre 03: Johannesburg, South Africa; Centre 04: Durban, South Africa
Centre 05: DTHF, Cape Town, South Africa
